# Circadian light therapy and light dose for depressed young people: a systematic review and meta-analysis

**DOI:** 10.3389/fpubh.2023.1257093

**Published:** 2024-01-08

**Authors:** Ranpeng Chen, Yonghong Yan, Xiang Cheng

**Affiliations:** ^1^School of Architecture and Urban Planning, Chongqing University, Chongqing, China; ^2^Key Laboratory of New Technology for Construction of Cities in Mountain Area, Chongqing University, Chongqing, China

**Keywords:** light therapy, circadian light, circadian stimulus, youth, meta-analysis, dose response

## Abstract

**Background:**

Empirical evidence has shown that light therapy (LT) can reduce depression symptoms by stimulating circadian rhythms. However, there is skepticism and inconclusive results, along with confusion regarding dosing. The purpose of this study is to quantify light as a stimulus for the circadian system and create a dose-response relationship that can help reduce maladies among adolescents and young adults (AYAs). This will provide a reference for light exposure and neural response, which are crucial in the neuropsychological mechanism of light intervention. The study also aims to provide guidance for clinical application.

**Methods:**

The latest quantitative model of CL_A_ (circadian light) and CS_t,f_ (circadian stimulus) was adopted to quantify light dose for circadian phototransduction in youth depression-related light therapy. Articles published up to 2023 through Web of Science, Cochrane Library, Medline (OVID), CINAHL, APA PsycINFO, Embase, and Scholars were retrieved. A meta-analysis of 31 articles (1,031 subjects) was performed using Stata17.0, CMA3.0 (comprehensive meta-analysis version 3.0) software, and Python 3.9 platform for light therapy efficacy comparison and dose-response quantification.

**Results:**

Under various circadian stimulus conditions (0.1 < CS_t,f_ < 0.7) of light therapy (LT), malady reductions among AYAs were observed (pooled SMD = −1.59, 95%CI = −1.86 to −1.32; *z* = −11.654, *p* = 0.000; *I*^2^ = 92.8%), with temporal pattern (*p* = 0.044) and co-medication (*p* = 0.000) suggested as main heterogeneity sources. For the efficacy advantage of LT with a higher circadian stimulus that is assumed to be influenced by visualization, co-medication, disease severity, and time pattern, sets of meta-analysis among random-controlled trials (RCTs) found evidence for significant efficacy of circadian-active bright light therapy (BLT) over circadian-inactive dim red light (SMD = −0.65, 95% CI = −0.96 to −0.34; *z* = −4.101, *p* = 0.000; *I*^2^ = 84.9%) or circadian-active dimmer white light (SMD = −0.37, 95% CI = −0.68 to −0.06; *z* = −2.318, *p* = 0.02; *I*^2^ = 33.8%), whereas green-blue, circadian-active BLT showed no significant superiority over circadian-inactive red/amber light controls (SMD = −0.21, 95% CI = −0.45 to 0.04; *z* = −2.318, *p* = 0.099; *I*^2^ = 0%). Overall, circadian-active BLT showed a greater likelihood of clinical response than dim light controls, with increased superiority observed with co-medication. For pre-to-post-treatment amelioration and corresponding dose-response relationship, cumulative duration was found more influential than other categorical (co-medication, severity, study design) or continuous (CS_t,f_) variables. Dose-response fitting indicated that the therapeutic effect would reach saturation among co-medicated patients at 32–42 days (900–1,000 min) and 58–59 days (1,100–1,500 min) among non-medicated AYAs. When exerting high circadian stimulus of light therapy (0.6 < CS_t,f_ < 0.7), there was a significantly greater effect size in 1,000–1,500 min of accumulative duration than <1,000 or >1,500 min of duration, indicating a threshold for practical guidance.

**Limitations:**

The results have been based on limited samples and influenced by a small sample effect. The placebo effect could not be ignored.

**Conclusions:**

Although the superiority of LT with higher circadian stimulus over dimmer light controls remains unproven, greater response potentials of circadian-active BLT have been noticed among AYAs, taking co-medication, disease severity, time pattern, and visual characteristics into consideration. The dose-response relationship with quantified circadian stimulus and temporal pattern had been elaborated under various conditions to support clinical depression treatment and LT device application in the post-pandemic era.

## 1 Introduction

The circadian stimulus of light therapy (1), also known as bright light therapy (BLT), has been found to have positive clinical outcomes in reducing symptoms of depression. Since the dysfunction of the circadian system has been strongly linked to psychological disorders, light therapy has been particularly successful in treating such conditions (2). However, skeptics contend that light therapy's efficacy may be little better than a placebo under great varieties of light administration protocols (e.g., white light or monochromatic light, various intensities) (3), as well as malady conditions. The skepticism surrounding the efficacy of light therapy for treating a wide range of maladies is rooted in the uncertainty of light dosing (2), whether in monotherapy studies or combination cases (4). Therefore, quantifying the amount of light can help establish a reliable and predictable relationship between light therapy and reductions in relevant disorders. This study aims to verify the efficacy of light therapy by quantifying the circadian stimulus and duration time as vital parameters of light dose for the circadian system among adolescents and young adults (AYAs).

### 1.1 Youth depression and circadian dysrhythmias

There is substantial evidence that links circadian misalignment with depression among young people (5, 6), e.g., major depressive disorder (7), bipolar disorder (8), unipolar depressive disorders (9), delayed sleep phase (DSP), attention deficit hyperactive disorder (ADHD) (10), and “circadian” depression clinical phenotype (11). Besides classical diagnostic depressive subtypes, other primary (e.g., post-natal, peri-menopausal, late-onset) and secondary (e.g., post-infective, comorbid pain syndromes) depressive subtypes may also be linked with underlying circadian dysfunction (12, 13). Circadian responses to bright light therapy are mainly based on the circadian phototransduction mechanism, which is considered beneficial for the treatment of SAD (seasonal affective disorder) (14, 15), NSD (non-seasonal affective disorder) (16), BD (bipolar disorder) (17), MDD (major depressive disorder) (18), ADHD (attention deficit hyperactivity disorder) (19), Parkinson (20), Alzheimer's disease (21), antepartum depression (22), and CRSWDs (circadian rhythm sleep-wake disorders) (23)-related circadian rhythm disturbances, depression, and sleeping problems. Furthermore, there is empirical evidence that depressed youth have been prescribed not only light therapy but also chronotherapy (which is a combination of light therapy and sleep deprivation—wake therapy) for greater circadian adjustment and anti-depressive effect stabilization in seasonal affective disorders and non-seasonal unipolar and bipolar depression (24). Though overall treatment effectiveness may be inconclusive (25), the dose effect and dose response of light therapy have been hypothesized and explored in numerous protocols, e.g., dose equivalence assumption on a duration × light intensity basis (4, 26). Additionally, the temporal pattern has also been found to be related to dose-dependent efficacy and has been studied (27); for instance, depression severity has been found (22) or presumed (28) altering along with varied duration or total treatment period (29–31). Overall, the lack of an accepted standard definition of adequate dosing for experimental light treatment, along with controversial presumptions and results, has made it challenging to assess the effectiveness of such treatment.

### 1.2 Circadian phototransduction and current quantifying model

Human circadian phototransduction, which is closely related to the non-visual effects of light, should be distinguished from visual effects. Multiple neural channels emanate from the retina, each with different spectral sensitivities that convert optical radiation into neural signals. Yet, the quantitative photopic luminous efficiency function V(λ) defined by CIE (Commission Internationale de l'Eclairage) and the photometric illuminance (lux) (for quantifying light therapy devices) is not relevant to all of these neural channels (32). Circadian phototransduction is a non-visual effect of light primarily based on photoreceptor-like intrinsically photosensitive retinal ganglion cells (ipRGCs) that send light information to the biological clock in the hypothalamic suprachiasmatic nucleus (SCN), which then synchronizes biological rhythms and projects signals to (including but not limited to) the ventrolateral preoptic (VLPO) nucleus, extended amygdala (33), pineal body, etc. The synthesis of melatonin in the pineal gland and core body temperature rhythmically rise and fall over a 24-h period and are used experimentally as markers of the endogenous rhythm (34); mood oscillates in parallel to core body temperature and is strongly influenced by the circadian process (35). Besides, other chronobiological effects of light are based on SCN-related physiological mechanisms (36). The human retina has five photoreceptors that contribute to the circadian system phototransduction (37). These include rods, cones, and ipRGCs, along with their respective photopigments, retinal opsin proteins such as melanopsin, rhodopsin, S-, M-, and L-cone opsin. Among these photoreceptors, ipRGCs, and melanopsin are mostly involved in the process, and they are more sensitive to “blue” wavelength at around 480 nm (38). S-cone-opic also plays a role in the circadian system phototransduction (39, 40). Therefore, the relative potency of light sources for light therapy would be more influenced by the amount of short-wavelength light mediated by the melanopsin system than the illuminance (lux) at eye level (41). Short-wavelength enriched light with lower intensity may have a stronger effect on the circadian system than visually stronger light.

The circadian light (CL_A_) model, which has been undergoing retinal neurophysiology and psychophysics experiments (42, 43), can be explained as a spectral weighting function suitable for SCN-oriented circadian responses. The CL_A_ model can quantify a functional relationship between optical radiation incident on the retina and the spectral, temporal, and absolute responses of the SCN. The circadian stimulus (CS) was developed as the operating range of the circadian system from threshold to saturation, taking nocturnal melatonin suppression as the outcome measure (42). These models have photoreceptors (cones, rods, ipRGCs) and retina amacrines and their neuroanatomical and neurophysiological interactions (43) and have shown a response magnitude characteristic of different amounts of spectrally weighted optical radiation of the single circadian phototransduction circuit (42). Moreover, the latest models of circadian stimulus (CS) and circadian light (CL_A_) have reduced discrepancy in response to “warm” and “cold” light sources, optimized duration, and light exposure distribution; therefore, in this study, they were adopted to quantify circadian phototransduction.

It has also been proven that besides the SCN-dependent circadian phototransduction circuit, separate non-circadian (34) or SCN-independent pathways exist with light effects on mood that may be direct, immediate, and sustained. For instance, the simultaneous inhibition of the sleep-inducing ventrolateral preoptic (VLPO) and the activation of the monoaminergic (44), thalamic, and hypothalamic regions (45) are involved in the control of mood and alertness. Likewise, the orexinergic and monoamine-dependent pathways impact mood. Moreover, there are also emotional processing pathways (light-sensitive circuits) in the cortical system, frontal cortex, and limbic system that ameliorate depressive symptoms (46). For example, the ipRGCs-vLGN/IGL (thalamic region)-LHb (lateral habenula) pathway may be the crucial sluice through which light ameliorates depression-like behaviors (47). However, for this study, we did not adopt indicators besides CS because light dose-related experiments are limited or have merely been verified in animal models (48), and there are huge differences in the efficacious light dose between humans and other species (49).

### 1.3 Scoping “circadian” treatment for young people

Depressive disorders are among the most prominent health problems among young people. Extensive research efforts have reported unsatisfactory outcomes among AYAs who have undergone prescribed medication side-effects, treatment resistance, breakthrough depressive symptoms, much lower response rates under combined medication and psychotherapy compared with adults (50), or deficient evidence for pharmaxgical treatments on comorbidity of depression (51), or potential recurrence risk (52). However, light therapy has shown potential for AYAs suffering from these issues. Additionally, depression and sleep disorders exhibit high comorbidity among youth (53); all these have urged light as a zeitgeber for synchronization, as well as a non-invasive treatment for depression. Current evidence has shown BLT is likely well-tolerated in adolescents but pointed out that the highly variable selection of light dosing presents a challenge in comparing treatment response and tolerability (54).

It is noteworthy that age exerts influence on light therapy in both visual and non-visual ways. Older people normally receive decreased retinal illumination due to reduced pupil size, increased ocular lens absorption (55), and other substantial changes in visual organs. Correspondingly, young people obtain higher lens transmittance, especially for short-wavelength light that peaks non-visual sensitivity, which is shifted to longer wavelengths in older people (56). CIE has also outlined the sensitivity variation of light-sensitive photoreceptors (38); compared to 32-year-old reference observers, populations aged 22 and 42 expressed weighted, fluctuated sensitivity, which may influence synchronizing input and melatonin suppression (57). Phase and amplitude of circadian functions are also related to age, such as alterations in SCN-related molecular and neuronal factors (58) and output levels [e.g., VLPO and pineal gland (59)]. Therefore, this study focuses on the current circadian aspect of light therapy in the depression treatment of the young population.

In this study, the therapeutic effect of circadian light therapy among AYAs will be quantitatively explored between circadian stimulus (defined by certain spectrum, illuminance, exposure duration time, and lighting distribution factor) and reductions in relevant maladies, using mainly the SMD values of clinical depression measurement scales as outcomes.

## 2 Materials and methods

### 2.1 Literature research

**Figure 1:**
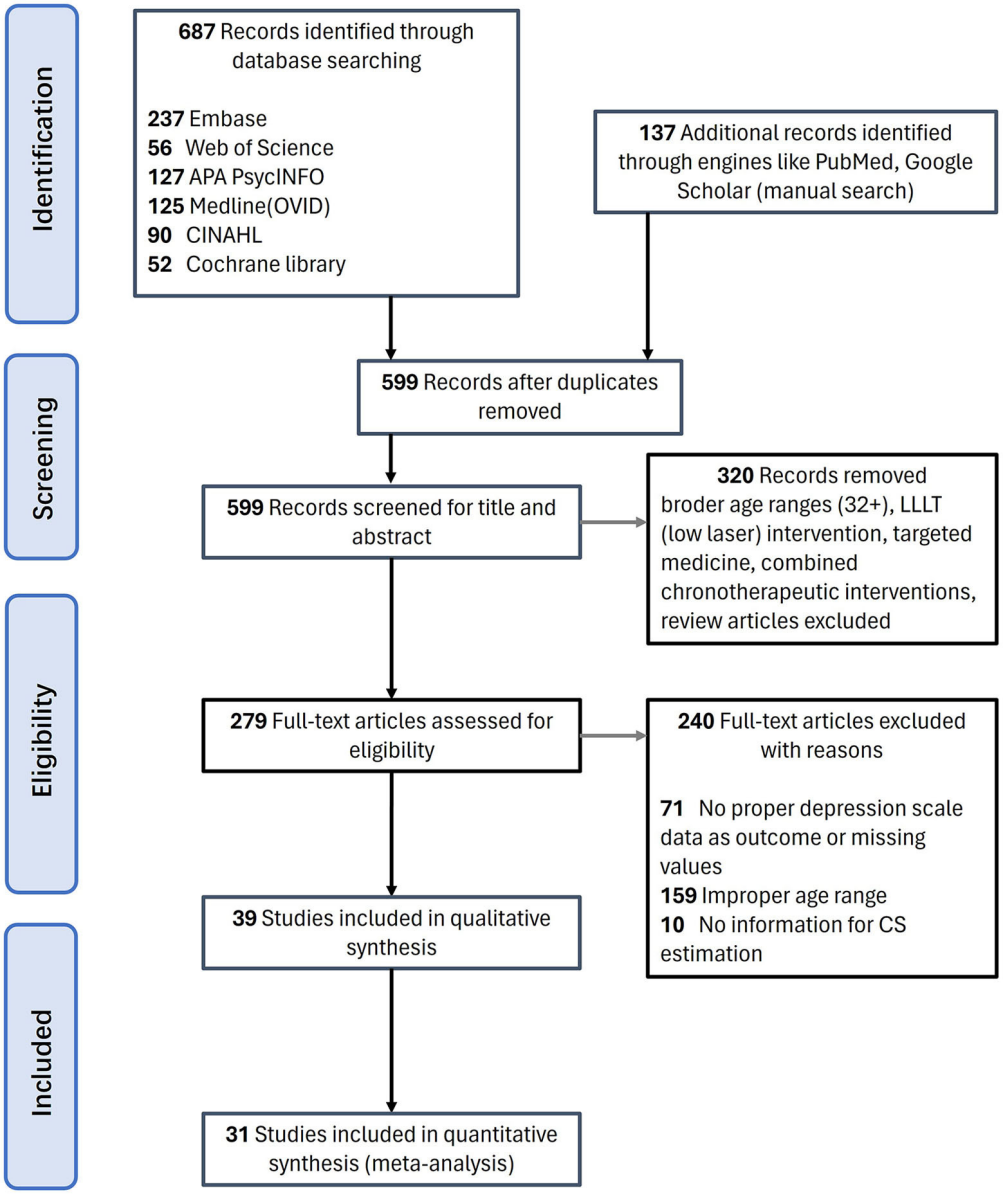
Literature search results following PRISMA flow.

This systematic review study was registered with PROSPERO under code number CRD42022375211 and was conducted following Preferred Reporting Items for Systematic Reviews and Meta-Analysis (PRISMA) guidelines (60). Articles published up to 2023 were searched through Web of Science, Embase, CINAHL, Cochrane Library, Medline (OVID), APA PsycINFO databases, as well as websites of the National Library of Medicine, ClinicalTrials.gov and Scholars. Multiple databases were also searched simultaneously using PubMed. Advanced searches with MeSH (medical subject heading) terms were included when available. The search strategy contained the following terms: (light therapy OR light treatment OR bright light therapy OR BLT OR chronotherapy OR phototherapy OR wake and light therapy) AND (youth OR adolescent OR students OR young adults OR teens OR teenagers) AND (depression OR depressive OR mood disorder). The detailed strategies can be checked in Supplementary Table S1. Full text was required but not restricted to English. The preliminary screening results were 90 in CINAHL, 52 in Cochrane Library, 237 in Embase, 125 in Medline (OVID), 127 in APA PsycINFO, and 56 in Web of Science. Additional studies that had not been captured by the original database search were retrieved mainly through Google Scholar. After removing duplication, 599 articles were left (Figure 1).

### 2.2 Inclusion criteria

Studies that met the following criteria were included: (A) The subjects are youth or young adults who averagely aged < 32 years—the age considered responding consistently to light stimulus in circadian phototransduction progress as addressed; (B) Must have bright light therapy as primary independent intervention, no sleep deprivation combined, since potential therapeutic effect may be disturbed by sleep deprivation; (C) Only difference between experiment and control groups should be BLT treatment, that when combined with antidepressants, it must be equally administered in both intervention and control to rule out the effect of the adjunct treatment; (D) Details of BLT included (e.g., specific device, light sources, illumination, correlated color temperature, continuous duration) for CS_t,f_ calculation; (E) Outcomes reported in standardized depression scales, e.g., Hamilton Depression Scale HAMD, HIGH-SAD, Hospital Anxiety and Depression Scale (HADS), Beck Depression Inventory BDI-II, Montgomery Asberg Depression Rating Scale (MADRS), Center for Epidemiologic Studies Depression Scale (CES-D) or other scales with similar structures proved reliable and validate among worldwide AYA (61).

Studies that met the following criteria were excluded: (1) Broader age groups (average age >32); (2) LLLT (low-level laser therapy) treatment studies; (3) Abstracts, case reports, case series, or literature reviews excluded.

### 2.3 Data extraction

Among the 599 articles excluding duplication (Figure 1), the research team reviewed the titles and abstracts of all downloaded literature for initial screening. The articles were further filtered based on the relevancy of contents, and 279 were left. After thorough screening for relevancy and eligibility through a full-text review, we finalized 31 pieces of literature that met the criteria. Then, two dependent reviewers, R.P. and X.C., extracted the main information shown in Table 1: (A) Subject information, including age and gender; (B) Experimental design. BLT period, duration (weeks, times, exposure minutes); (C) Sample size of experiment group, control group; (D) Light therapy devices, spectra, illumination at eye level, lighting details, and distribution, which are parameters needed for CS_t,f_ calculation; (E) Pre-to-post-treatment quantitative outcomes of measurement scales (detailed information can be checked in Supplementary Table S2).

**Table 1:** Characteristics of the included trials (*n* = 31).

**Subgroup/ Study/ Country**	**Diagnosis**	**Duration**	**Accumulative duration day-minutes**	**Treatment condition; wavelength; intensity**	**Control condition; wavelength; intensity**	**CS_t,f_**	**Mean age (years ±SD)**	**Sample size (*n*)**	**Sex: male/female (*n*/*n*)**	**Mood measure**	**Antidepressant medication**
**Random-controlled trials**											
**Diagnosed with depression and medicated**											
Bogen et al. (87), Germany	MDD (BDI-II), 6 in each group with SAD pattern	2 weeks, daily morning 45 min	10 days; 450 min	white light fluorescent box (Davita), 10,000 lux	wake therapy + LT	Tx: CS_t0.75_ = 0.665	Tx: 15.75 ± 1.017; Control: 16.16 ± 1.275	Tx: *n* = 37; Control: *n* = 25	Tx: 2/35; Control: 3/22	BDI-II	Partial co-medication
Youngstedt et al. (90), USA	Anxiety (DSM-IV), MDD, PTSD, GAD balanced	4 weeks, daily morning 45min	7 days; 315 min 14 days; 630 min 21 days; 945 min 28 day; 1260 min	Blue-enriched white light LED; 460 nm (Litebook); 3,000 lux	Inactivated negative ion generator (INIG)	Tx: CS_t0.75_ = 0.652	Tx: 22.0 ± 1.0; Control: 21.4 ± 0.6	Tx: *n* = 17; Control: *n* = 16	Tx: 5/12; Control: 3/13	BDI	Partial co-medication
Goel et al. (81), USA (81)	29.7% depressed	3 days, daily evening 30 min	3 days; 90 min	white light fluorescent lamps, 10,000 lux (3,000 K)	Negative ion generator, sound	Tx: CS_t0.5_ = 0.635	Overall: 19.4 ± 1.7	BLT: *n* = 29; sound stimulus: *n* = 30; High-density air flow rate: *n* = 29; low-density air flow rate: *n* = 30	Overall: 49/69	POMS	Partial co-medication
Richardson and Gradisar (76), Australia^*^	30 self-reported MDD	3 weeks, daily morning 30–60 min, 3 times per week	3 days; 150 min 6 days; 300 min 9 days; 450 min	Green light LED (Re-timer); 507 nm; 112 lux	Amber light LED (Re-timer); 643 nm; 112 lux	Tx: CS_t0.78_ = 0.242; Control: CS_t0.8_ < 0.1	Tx: 16.07 ± 2.4; Control: 15.6 ± 2.2	Tx: *n* = 30; Control: *n* = 30	Tx: 11/19; Control: 11/19	SMFQ	Partial co-medication
Flory et al. (97), USA^*a^	MDD with seasonal pattern (SIGH-SAD-SR, DSM-IV)	12 days, daily morning 30 min	12 days; 360 min	White light fluorescent boxes, 10,000 lux (4,100 K)	Red light fluorescent box; 300 lux	Tx: CS_t0.5_ = 0.642–0.663; Control-DRL: CS_t0.5_ = 0.129	Overall: 20.8 ± 5.69	BLT: *n* = 19; DRL: *n* = 16; high-density negative ions (HDNI): *n* = 18; low-density negative ions (LDNI): *n* = 20	All females	SIGH-SAD-SR, HAM-D, BDI-II	All co-medication
LaRosa et al. (68), USA	Cancer-related fatigue	8 weeks, 30 min within 1 h of waking	8 weeks; 1,680 min	Blue-enriched white light LED; 460 nm (Litebook); 3,000–10,000 lux	red Light LED; 680 nm (The Litebook;50 lux	Tx: CS_t0.5_ = 0.64 (0.549–0.679); Control: CS_t0.5_ < 0.1	Overall: 15.96 ± 2.41	Tx: *n* = 21^c^; Control: *n* = 23	Tx: 8/13; Control: 11/12	CDI-II	co-medication
Spezzano (88), USA^*^	SAD	3 weeks, daily morning 30 min	7 days; 210 min 14 days; 420 min 21 days; 630 min	White light fluorescent box (SunBox) 10,000 lux	Inactivated ion generator	Tx: CS_t0.5_ = 0.658	Tx: 19.75 ± 1.4; Control: 19.7 ± 1.2	Tx: *n* = 20; Control: *n* = 20	Tx: 9/11; Control: 7/13	SIGH-SAD, BDI-II	co-medication
Blouin et al. (71), Canada^*^	Bulimia nervosa (DSM-III-R), 13 MDD, 10 SAD (SPAQ)	1 week, daily 2 h in the early evening, 17:00–19:00 p.m.	7 days; 840 min	White light fluorescent box (Duratest); 2,500 lux	White light fluorescent box (Duratest); 500 lux	Tx-2 500 lux: CS_t2.0_ = 0.669; Control- 500 lux: CS_t2.0_ = 0.552	Overall: 27.9 ± 8.0	Tx: *n* = 9; Control: *n* = 9	Tx: 0/9; Control: 0/9	SIGH-SAD, BDI,	Partial co-medication
Braun et al. (70), USA	Bulimia nervosa (SCID), some MDD (SCID)	3 weeks, daily morning 90 min	21 days; 1,890 min	White light fluorescent box (Apollo), 10,000 lux	Red fluorescent light; 50 lux	Tx: CS_t1.5_ = 0.690; Control: CS_t1.5_ < 0.01	Tx: 30.50 ± 7.3; Control: 30.50 ± 8.6	Tx: *n* = 15; Control: *n* = 16	Tx:0/15; Control: 0/16	SIGH-SAD, BDI,	Co-medication
Bais et al. (73), the Netherlands^*b^	MDD (SCID, DSM-5)	6 weeks, daily morning 30 min	7 days; 210 min 14 days; 420 min 21 days; 630 min 28 days; 840 min 35 days; 1050 min 42 days; 1260 min	White light LED (EnergyUp HF3419/01, Philip); 9,000 lux (5,000 K)	Red LED light; 100 lux (2,700 K)	Tx: CS_t0.5_ = 0.657; Control: CSt_0.5_ < 0.1	Tx: 31.9 ± 4.4; Control: 31.9 ± 5.3	Tx: *n* = 33; Control: *n* = 34	Tx: 0/33; Control: 0/34	SIGH-SAD, HAMD-17, EPDS	Partial co-medication
**Randomized controlled trials**											
**Depressed but non-medicated**											
Bogen et al. (87), Germany^*^	MDD (ICD-10), ≥20 points (BDI-II)	2 weeks, 45 min of morning BLT, 5 times a week	2 weeks; 450 min	White light fluorescent box (Davita), 10,000 lux	White light LED box (Davita Luxor), 100–150 lux	Tx: CS_t0.75_ = 0.665; Control: CS_t0.75_ < 0.1	Tx: 15.4 ± 1.6; Control: 15.3 ± 1.5	Tx: *n* = 30; Control: *n* = 27	Tx: 11/19; Control: 4/23	BDI-II	No co-medication
Jiang et al. (31), China^*^	Non-seasonal subthreshold depression (HAMD-24).	8 weeks, daily morning 30 min	28 days; 840 min 56 day; 1,680 mi	white cold LED, 5,000 lux (5,000K)	A:500 lux white cold LED B: Waiting list	Tx- 5,000 lux: CS_t0.5_ = 0.639 (0.622- 0.648); Tx−500 lux: CS_t0.5_ = 0.277(0.272–0.30)	Tx-5,000 lux: 21.18 ± 2.31; Tx-500 lux: 21.49 ± 2.35; Control: 21.38 ± 2.22	Tx- 5,000 lux: *n* = 51; Tx−500 lux: *n* = 51; Control: *n* = 42	Tx- 5,000 lux: 16/35; Tx−500 lux :13/38; Control: 15/27	BDI-II, HAMD-24, SAI	No
Janas-Kozik et al. (29), Polland^*^	Anorexia nervosa (AN-R) (DSM-IV), ≥17 points (21-item HDRS)	6 weeks, 30 min daily morning	7 days; 210 min 14 days; 420 min 21 days; 630 min 28 days; 840 min 35 days; 1,050 min 42 days; 1,260 min	White light fluorescent box; 10,000 lux	no LT + CBT	Tx: CS_t0.5_ = 0.60–0.69	Tx: 17.8 ± 1.34; Control: 17.0 ± 1.34	Tx: *n* = 12; Control: *n* = 12	Tx:0/12; Control: 0/12	HDRS	No co-medication
Donmezt al. (30), Turkey^*^	MDD (DSM-5 criteria), ≥12 points (EPDS)	3 weeks, daily morning 45 min	7 days; 315 min 14 days; 630 min 21 days; 945 min	White light LED lamp (Beurer), 10,000 lux (2,500 lux at eye, 5,000 K)	White light LED lamp (Beurer), 500 lux (125 lux at the eye)	Tx: CS_t0.75_ = 0.59; Control: CS_t0.75_≈0.12	Tx: 29.73 ± 6.57; Control: 28.0 ± 3.8	Tx: *n* = 15; Control: *n* = 15	Tx:0/15; Control: 0/15	MADRS, HAM-D, EPDS	No
Epperson et al. (22), USA	MDD (DSM-IV), 1 with seasonal pattern (DSM-IV)	5 weeks, daily 60 min,	35 days; 2,100 min	White light fluorescent box (HealthLight, SphereOne); 7,000 lux	White light fluorescent box; 500 lux	Tx: CS_t1.0_ = 0.678–0.682; Control: CS_t1.0_ = 0.497	Overall: 32.10 ± 3.9	Tx: *n* = 4; Control: *n* = 5	Tx: 0/4; Control: 0/5	SIGH-SAD	No
Grandner (82), USA^*^	some with minimal to mild depression	12 days, 150 min prior to usual wake time (30 min intensity 0–100%)	Tx: 10.7 days-1,605; Control: 11.3 days-1,695	Green light LED; 500 nm; 10,000 lux	red light LED; 0.5 lux	Tx: CS_t2.5_ = 0.692–0.694; Control: CS_t2.5_ < 0.1	Tx: 23.13; Control: 22.13	Tx: *n* = 15; Control: *n* = 15	Tx: 15/0; Control: 15/0	QIDS-SR	No
**Quasi-experimental trials**											
**Depressed and medicated**											
Swanson et al. (74), USA	MDD (DSM-V), ≥20 points (SIGH-SAD)	5 weeks, daily morning 60 min	35 day-1,050–2,100	Green light LED (Re-timer); 507 nm; 506 lux	—	Tx: CS_t0.5_ = 0.451; CS_t1.0_ = 0.557	Tx: 32.30 ± 3.27	Tx: *n* = 10	0/10	SIGH-SAD	Partial co-medication
Kirschbaum-Lesch et al. (64), Germany	MDD (BDI-II)	4 weeks, weekdays daily morning 30 min	10 day-600 20 day−1,200	Blue-enriched white light LED (Luminette^®^); 468 nm; 10,000 lux	—	Tx: CS_t0.5_ = 0.686	Tx: 15.74 ± 1.14	Tx: *n* = 39	7/32	BDI-II	Partial co-medication
House et al. (62), USA	MDD, SAD	4 weeks, 15mins for 1st week, weekday morning 30 min next 3 weeks	20 day-525	White light fluorescent box (NorthStar); 10,000 lux (4,100 K)	—	CS_t0.5_ = 0.667–0.668	Tx: 19–21	Tx: *n* = 79	18/61	BDI-II	Partial co-medication
Papatheodorou and Kutcher (83), Canada	Bipolar disorder (DSM-III-R)	1 week, 7:00–9:00 a.m. +19:00–21:00 p.m., daily 45–60 min	7 days-650	Cool-white fluorescent box; 10,000 lux	—	Tx: CS_t0.75_ = 0.679 (0.663–0.683); CS_t1.0_ = 0.685 (0.682–0.692)	Tx:19.4 ± 2	Tx: *n* = 7	2/5	BDI-II	All co-medication
Nixon et al. (92), Canada	At least mild symptoms on QIDS-SR16 or QIDS-A17	4 weeks, daily morning 45–60 min	10 day-735 (630–840) 21 days-(620–1,240 min)	Green light LED (Re-timer); 507 nm; 112 lux	—	Tx: CS_t0.78_ = 0.242; CS_t1.0_ = 0.287	Tx: 21.2 ± 1.0	Tx: *n* = 24^d^	4/20	BDI-II	Partial co-medication
Kopp et al. (67), USA	80% at least mild MDD	1 week, daily morning 30 min	7 days-210	White light fluorescent box Sun Touch Plus Light ©; 10,000 lux (17,000 K)	—	Tx: CS_t0.5_ = 0.669–0.684	Tx: 27.7 ± 8.5	Tx: *n* = 30	17/13	QIDS-C	Partial co-medication
Ricketts et al. (69), USA	85.7% TD (tic disorder), 57.1% MDD	2 weeks, daily morning 60 min	14 day-840	Re-Timer Light Therapy Glasses, 112 lux, peak 507 nm	Healthy controls	Tx: CS_t0.75_ = 0.235	Tx: 27.86 ± 5.20; Control: 31.75 ± 8.49	Tx: *n* = 14; Control: *n* = 20	Tx:10/4	DASS	Partial co-medication
Bromundt et al. (72), Switzerland	BPD (DSM-IV), 7 (50%) SAD, 2 (14%) subsyndromal form	3 weeks, daily morning 30–40 min	18 day-630	white light fluorescent lamp (Daylight^®^); 8,000 lux	healthy females + oLT	Tx: CS_t0.5_ = 0.647–0.669	Tx: 30.1 ± 6.0; Control: 25.7 ± 4.8	Tx: *n* = 14; Control: *n* = 10	Tx: 0/14; Control: 0/10	SIGH-ADS-SR, BDI-II	Partial co-medication
**Non-depressed and non-medicated**											
Lee et al. (79), USA	healthy first-time mothers	3 weeks, daily morning 30 min	18 day-550	Blue–green light LED; 500 nm; 3,000–8,000 lux	Dim red light	Tx: CS_t0.5_ = 0.663–0.688	Tx: 24.4 ± 5.4; Control:29.1 ± 6.7	Tx: *n* = 16; Control: *n* = 14	Tx: 0/16; Control: 0/14	EPDS	None
Sasseville et al. (80), Canada	healthy	30 min at 3:00 am	1 day-30	Blue-enriched white light LED (Litebook^®^); 1,420 lux	Amber light LED (blue-blocking), 580 nm; 1,150 lux	Tx: CS_t0.5_ = 0.556; Control: CS_t0.5_ < 0.1	Tx: 24.5 ± 1.5 (21–26 years); Control: 27.4 ± 1.8 (25–30 years)	Tx: *n* = 10; Control: *n* = 10	Tx: 5/5; Control: 4/6	VAS	None
Raikes et al. (78), USA	Mild traumatic brain injuries, no Axis I disorders (DSM-IV)	6 weeks, daily morning 30 min	42 day-1,260	blue light LED (Philips goLITE BLU); 469–480 nm; 214 lux	amber light LED box; 578 nm, 188 lux	Tx: CS_t0.5_ = 0.580–0.585; Control: CS_t0.5_ < 0.1–0.135	Tx: 25.53 ± 8.65; Control: 26.63 ± 7.62	Tx: *n* = 17; Control: *n* = 18	Tx: 5/12; Control: 8/10	BDI-II	None
Huang et al. (63), China	Insomnia (ISI score > 14)	10 days, daily >30 min, evening shift exposure 19:30–20:30 p.m., night shift exposure 23:00–24:00 p.m.	10 days-300	White light LED (Apollo briteLITE 6); 5,000–6,000 lux	A sham lightbox of much lower intensity or dim red light; also wore dark sunglasses	Tx: CS_t0.5_ = 0.651 (0.638–0.656)	Tx: 30.2 ± 4.5; Control: 30.3 ± 4.7	Tx: *n* = 46; Control: *n* = 46	Tx: 0/46; Control: 0/46	HADS, HADS-D,	Partial co-medication
Li et al. (75), China	DSWPD	1 week, daily morning 60 min	7 days-420	Green-blue light LED (PEGASI^®^); 470 nm; 20–1,200 lux	—	Tx: CS_t1.0_ = 0.302–0.507	Tx: 29.73 ± 8.98; Control: 34.9 ± 10.80	Tx: *n* = 15; Control: *n* = 15	Tx: 4/11	HAMD-24	None
van Kol (77), the Netherlands	Burnout	2 weeks, weekdays day morning 20–30 min	10 day-300	White light LED (EnergyUp HF3419/01, Philips); 984–1,088 lux (4,590 K)	Red light LED (Philips-7001831PH); 205 lux	Tx: CS_t0.5_ = 0.401–0.420; Control: CS_t0.5_ < 0.1	Overall: 28.28 ± 14.10	Tx: *n* = 29;	Tx:10/19	BDI-II-NL (Dutch),	None
Danielsson et al. (65), USA	DSPD	2 weeks, daily morning 30–45 min	14 day-420	White light fluorescent lamp (Brite LITE 6, Philips)	LT+CBT	Tx: CS_t0.5_ = 0.675 (0.656–0.673); CSt_0.75_ = 0.684 (0.671–0.683)	Tx: 22 ± 3; Control: 22 ± 2	Tx: *n* = 19; Control: *n* = 17	Tx:9/10; Control:10/7	HADS-D	Partial co-medication

^*^Studies included in between-group comparison with identical baseline or response details.

^a^11 remained on prescribed medications other than psychotropic drugs, eight remained on a psychotropic medication regimen of either an SSRI (six subjects) or a norepinephrine/dopamine reuptake inhibitor (two subjects).

^b^11 started an SSRI, 1 woman in the postpartum period (both sertraline), one with an antipsychotic (quetiapine), and one with a benzodiazepine (temazepam) postpartum. The escitalopram dose increased in the postpartum period of one participant.

^c^Although 26 patients in the BWL group and 25 patients in the DRL group were randomized, data were available only for 21 in the BWL group and 23 patients in the DRL group.

^d^31 participants were recruited, but only 24 completed at least 2 weeks of the intervention and were included in the analyses: adherence data was missing for 1 participant. During the 4 weeks (28 days) of intervention, 12 participants (50%) reported using the light therapy glasses in the morning for 30–60 min between 22 and 29 days, six participants (25%) reported using them between 14 and 21 days, and 5 participants (21%) reported using on <14 days.

MDD, Major Depressive Disorder; OCD, Obsessive–Compulsive Disorder; PTSD, Post-traumatic Stress Disorder; SP, Social Phobia; ADA, Alcohol/Drug abuse; GAD, Generalized Anxiety Disorder; DSPD, delayed sleep phase disorder; DSWPD, Delayed Sleep-Wake Phase Disorder; CF, Cystic fibrosis; SCID, Structured Clinical Interview for DSM-IV Axis I Disorders; CGI, Clinical Global Impression of Severity scale; STAI, State–Trait Anxiety Inventory (Form Y2); QIDS, The Quick Inventory of Depressive Symptomatology self-report; LOS, length of stay; TD, Tourette's disorder; LT, light therapy; BWL, bright white light; CBT, cognitive– behavioral psychotherapy; EPDS, Edinburgh Postnatal Depression Scale; DSM, Diagnostic and Statistical Manual of Mental Disorders; HDRS, 21-item Hamilton Depression Rating Scale; HADS, Hospital Anxiety and Depression Scale; ISI, Insomnia Severity Index; HADS-A, anxiety subscale of the Hospital Anxiety and Depression Scale; HADS-D, depression subscale of the Hospital Anxiety and Depression Scale; SIGH-SAD, Structured Interview Guide for the Hamilton Depression Rating Scale-Seasonal Affective Disorder; SIGH-SAD-SR, Structured Interview Guide for the Hamilton Depression Rating Scale–Seasonal Affective Disorder Version–Self Rating; SDQ, The Strength and Difficulties Questionnaire (SDQ- emotional problems, hyperactivity, behavioral problems with peers and ability to socialize in 25 items); SAI, state anxiety inventory; HAMD-24, Hamilton Depression Rating Scale; HAM-A, Hamilton Anxiety Scale; ICD-10, International Statistical Classification of Diseases and Related Health Problems; SPAQ, Seasonal Pattern Assessment Questionnaire; BMI, body mass index; BDI-II, Beck Depression Inventory Second Edition; MAVS, mood visual analog scale; ESS, Epworth Sleepiness Scale; DASS, Depression Anxiety Stress Scale; KSS, Karolinska Sleepiness Scale; POMS, The Profile of Mood States Questionnaire; PSQI, Pittsburgh Sleep Quality Index; MBI, the Maslach Burnout Inventory; UBOS, Utrechtse Burnout Schaal; FAS, Fatigue Assessment Scale; BO-NKS, Burnout- Neurasthenia Complaints Scale; RPCSQ, the Rivermead Post-concussion Symptoms Questionnaire-The Functional; YBC-EDS, Yale-Brown-Cornell Eating Disorder Scale; LFS, Lee's Fatigue scale; CDI-II, Children's Depression Inventory, 2nd Edition; BSL-95, Borderline Symptom List; VAS, visual analog scales; SSS, Stanford Sleepiness Scale; NIMH-DIS-R, National Institute of Mental Health Diagnostic Interview Schedule-Revised; SMFQ, Short Mood and Feelings Questionnaire; SSRI, selective serotonin reuptake inhibitor; Tx, treatment condition; LED, light emitting diode.

### 2.4 Data analysis

Although the literature included patients with various pathologic features and was not limited to randomized controlled trials (RCT), the continuous outcomes variables of clinical depression measurement were recorded and analyzed. The mean (standard deviation) was derived as the main outcome at the starting point before intervention and the endpoint after intervention. The changes in scores were converted into the starting point and endpoint values. Heterogeneity among included studies was analyzed using χ2 tests (α = 0.05), and the magnitude was quantified in conjunction with Cochran's Q statistics and *I*^2^. The primary outcome was an improvement in depressive symptoms on a clinician-rated depression rating scale, including differences in endpoint scores on the scale between active and control conditions with intent-to-treat samples, analyzed using standardized mean differences (SMD). If the secondary outcomes were available (clinical response defined a priori as a 50% reduction on a clinician-rated depression rating scale, assumed more reported by RCTs), we calculated risk ratios and odds ratios for the categorical data. Correspondingly, the included studies were preliminarily categorized with additional consideration of co-medication × study design (Table 1). Besides, as light dose quantification had barely been reported in previous BLT-oriented reviews, the dose-response analysis was drawn from neighboring methodologies.

## 3 Results

### 3.1 Study characteristics

The selected 31 articles were mainly small-sample clinical trials (<30 subjects). Only four studies involved larger samples (31, 62–64). Twenty-two studies were randomized controlled trials (RCTs), three of which adopted intervention beyond light therapy: e,g., CBT (cognitive–behavioral psychotherapy) (29), LT + CBT (65), and wake therapy + LT (66) as control to testify whether BLT acted as an effective adjunctive intervention. Nine were non-randomized controlled experiments (quasi-experimental studies) that may have been launched because lighting properties (colors, levels) are intuitively recognized by the human eye (1); therefore, true blindness for light treatment studies is difficult to achieve.

Depression was mainly screened by the Diagnostic and Statistical Manual of Mental Disorders (DSM) criteria; similarly maladies, where depression was a frequent co-morbid condition. One article studied depressed adolescents with anorexia nervosa (29), one study focused on depression in co-morbidity with cystic fibrosis (67), one for cancer-related fatigue (CRF)/depression (68), one for Tourette's disorder/depression (69), two for bulimia nervosa/winter binge/depression (70, 71), one for borderline personality disorder (BPD) (72), four for perinatal/postpartum depression (22, 30, 73, 74), one for non-seasonal subthreshold depression (31). Besides, non-specific depressed non-depressed were also included, especially those accompanied by sleep disturbances. Three studies targeted people with Delayed Sleep-Wake Phase Disorder (DSWPD) (65, 75, 76). One article studied burnout (77), one was on insomnia/shift work disorder (63), and one focused on mild traumatic brain injury (mTBIs) that excluded Axis I mental disorders (78). One study was on healthy first-time mothers with low-birth-weight (LBW) infants (79). One study aimed to investigate the alerting effect of BLT; thus, only healthy people were included (80). The majority of participants in two studies were mainly healthy college students (81, 82), but depressed individuals constituted a certain proportion and were balanced among groups. In a few co-medication studies, BLT was accompanied by antidepressants or other treatments, but it could be guaranteed that BLT was the only variable.

In terms of intervention time, one study (83) carried out both morning and night BLT, two studies carried out BLT in the early evening (71, 81) and two experimented in late night (63, 80), and in the rest, BLT were all morning interventions. In terms of a consecutive duration time, 16 studies were carried out daily for consecutive 30–45 min of exposure, seven studies for 45–60 min, four studies for 50–60 min, one study for 90 min, one for daily 120 min, and one for 150 min. In terms of BLT devices, all but seven studies used light boxes or lamps, where light visors (six studies) and light masks (one study) were employed. As for active bright white light, treatment illuminance varied between 3,000 and 10,000 lux, and only a few adopted 2,500 lux (71). The glasses mainly emitted blue or green light at a much lower intensity, and the intervention CS_t,f_ (circadian stimulus) ranged from <0.1–0.7 (Supplementary Table S2). A CS = 0.3 in the original metric of CS for at least 1 h in the morning has been shown to improve sleep and reduce depression empirically (84, 85), while CS < 0.3 was not expected to considerably suppress nocturnal melatonin. However, these conclusions may be less convincing for depression amelioration since this value was merely hypothesized for group comparisons. The analogic CS_t_ metric also indicated no strict definition for high or low CS_t_ threshold (32). As the CS_t,f_ metric represents the instantaneous luminous stimulus for the circadian system (42), the vast majority of included studies considered responding to applied light intervention from circadian ways.

### 3.2 Risk-of-bias assessment

For randomized controlled trials (RCTs), the quality of evidence was evaluated with the revised Cochrane Risk of Bias Tool (86) (Supplementary Figure 1). One study adopted a randomizer box (87), six studies adopted computer software (31, 65, 68, 73, 76, 88), two studies adopted a random-number table (30, 81), one study adopted a random digit table (63), and one study adopted numbered, sequential, sealed envelopes (82). The remaining RCTs that indicated randomization sequence generation were also considered to be low risk in selection bias. Most studies owned moderate risk in allocation concealment since it was not reported (89). As for performance bias, seven studies showed moderate risk when participants were possibly non-blinded while specific examiners were blinded (29, 31, 65, 87, 90) or vice versa (22, 82). For attrition bias assessment, statistical approaches like LOCF (last observation carried forward) (87), BOCF (baseline observation carried forward) (78), and multiple imputations (66) were adopted, whereas bias still existed among dropouts. Linear Mixed Modeling (LLM) (69, 73, 76), General Linear Model (GLM) (66), and Hierarchical Linear Modeling (HLM) (77) were optimal, accounting for missing data, small sample size, and non-parametric distributions (91); thus, six studies were considered as at low risk. As for detection bias, seven studies where participants were informed of comparing different types of light therapy (30, 31, 66, 77, 78, 82, 87) were regarded as moderate risk since the blinding of outcome assessment remained unclear. For nine quasi-experimental/non-randomized controlled studies, the quality of evidence was evaluated using the Joanna Briggs Institute (JBI) Critical Appraisal Checklist for Quasi-Experimental studies (Supplementary Table S3). In two studies (83, 92), other measures remained unclear, and in two studies (69, 75), the baseline conditions could not be totally balanced, but the overall quality was eligible.

### 3.3 Circadian stimulus models and deduction

The evolving CLA and CS model and calculation formula had been optimized by Rea et al. (42) as follows (Eqs. 1, 2):


(1) $$\begin{array}{l} CLA\;=\;\{\begin{array}{cc} 1548\left[\begin{array}{c} \int Mc_{\lambda }E_{\lambda }d\lambda -a_{rod1}\left(\frac{\int V_{\lambda }^{{\;}^{\prime }}E_{\lambda }d\lambda }{\int V_{c\lambda }E_{\lambda }d\lambda +g_{1}\int S_{c\lambda }E_{\lambda }d\lambda }\right)\left(1-e^{\frac{-\int V_{\lambda }^{{\;}^{\prime }}E_{\lambda }d\lambda }{RodSat}}\right)\\ \;\\ +a_{b-y}\left(\int S_{c\lambda }E_{\lambda }d\lambda -k\int V_{c\lambda }E_{\lambda }d\lambda \right)\\ \;\\ -a_{rod2}\left(\frac{\int V_{\lambda }^{{\;}^{\prime }}E_{\lambda }d\lambda }{\int V_{c\lambda }E_{\lambda }d\lambda +g_{2}\int S_{c\lambda }E_{\lambda }d\lambda }\right)\left(1-e^{\frac{-\int v_{\lambda }^{{\;}^{\prime }}E_{\lambda }d\lambda }{Rodsat}}\right) \end{array}\right] & b-y≻0\\ 1548\left(\int Mc_{\lambda }E_{\lambda }d\lambda -a_{rod1}\left(\frac{\int V_{\lambda }^{{\;}^{\prime }}E_{\lambda }d\lambda }{\int V_{c\lambda }E_{\lambda }d\lambda +g_{1}\int S_{c\lambda }E_{\lambda }d\lambda }\right)\;\left(1-e^{\frac{-\int V_{\lambda }^{\prime }E_{\lambda }d\lambda }{RodSat}}\right)\right)\; & b-y≺0 \end{array} \end{array}$$

Where, *b* − *y* = ∫*S*_*cλ*_*E*_λ_*dλ* − *k* ∫ *V*_*cλ*_*E*_λ_*dλ*

k = 0.2616;

*a*_*b* − *y*_ = 0.21;

*a*_*rod*1_ = 2.30;

*a*_*rod*2_ = 1.60;

*g*_1_ = 1.00;

*g*_2_= 0.16;

*RodSat* = 6.5 W/m^2^, representing the half-saturation constant of bleached rod cells;

$V_{c\lambda }=\frac{\frac{V_{\lambda }}{mp_{\lambda }}}{\max\left(\frac{V_{\lambda }}{mp_{\lambda }}\right)}$, $S_{c\lambda }=\frac{\frac{S_{\lambda }}{mp_{\lambda }}}{\max\left(\frac{S_{\lambda }}{mp_{\lambda }}\right)}$

*E*_λ_: light source spectral irradiance

*Mc*_λ_: melanopsin sensitivity (corrected for crystalline lens transmittance) (93)

*S*_λ_: S-cone fundamental (94)

*mp*_λ_: macular pigment transmittance (95)

*V*_λ_: photopic luminous efficiency function (96)

${V\;}_{\lambda }^{{\;}^{\prime }}$: scotopic luminous efficiency function (96)

And,


(2) $$\begin{array}{l} \text{C}{\text{S}}_{\text{t},\text{f}}=0.7\times \left[1-\frac{1}{1+{\left(\frac{\text{t}\times \text{f}\times \text{CLA}}{355.7}\right)}^{1.1026}}\right] \end{array}$$

where *t* represents the exposure duration time and has been fitted by a duration of consecutive 0.5–3 h in previous studies (56). *f* represents the spatial distribution of circadian light exposure. As most of the related studies used light boxes, lamps, or light visors/glasses with less visual field covering than Ganzfeld, *f* was all valued as 1.0. CL_A_, representing circadian light illumination (circadian lux), was determined by the physiological properties of human eyes and light sources. As some spectral information of the devices was unavailable, similar CIE standard light sources or devices have been substituted for CS_t,f_ calculation (Table 1). Besides, we deduced that the accumulative light dose was also important and would contribute significantly to therapeutic efficacy. So that relationship was presumed as (Eq. 3):


(3) $$\begin{array}{l} {\text{P}}_{\left(\text{u}\right)}=\left[{\text{T}}_{\left(\text{u},\right)}\text{C}{\text{S}}_{\text{t},\text{f}}{\;}_{\left(\text{u}\right)}\right] \end{array}$$

where *P(u)* means the function of effect size, *T(u)* represents the function of accumulative exposure time, and CS_t,f_ represents a continuous circadian stimulus. However, whether T stands for min or days was not yet known and was verified through meta-analysis and dose-response fitting.

## 4 Clinical efficacy of circadian lighting

The clinical efficacy of circadian lighting was elaborated through between-group efficacy comparison, pre-to-post-treatment evaluation, dose-response, and saturation deduction. We anticipated heterogeneity of study methodologies with variability in diagnosis (depression or not), co-medication, lighting administration, duration, and so on and hence planned several exploratory subgroup analyses (protocol/flow can be checked in Supplementary Figure 2).

### 4.1 Main heterogeneity sources

Pre- to-post-treatment outcomes were sub grouped based on study characteristics, based on which preliminary meta-regression was stratified separately by categorical covariates: (a) co-medication, (b) disease severity, (c) light intensity, (d) light color, (e) accumulative duration of exposure to light during intervention, (f) circadian stimulus of light, (g) intervention period and follow-up, and (h) whether study designed as RCT (Supplementary Table S4). The *p*-values of meta-regression had implied co-medication (*p* = 0.01) and temporal pattern (*p* = 0.000) as main sources of heterogeneity. Subgroup meta-analysis was further adopted to quantify the between-study differences, with *I*^2^ values of 25, 50, and 75% reflecting a small, medium, and large degree of heterogeneity, and H values of 1, <1.2, 1.2–1.5, >1.5 indicating non, small, substantial, and considerable heterogeneity.

Subgroup meta-analysis subdivided by co-medication × depression (shown in Tables 1, 2 Subgroup A) yielded a significant effect of this covariate on the outcome (*p* = 0.000), suggesting significant difference existed between studies with co-medication, without co-medication, and non-depressed (non-medicated) groups. Significant differences also existed between temporal pattern subgroups during the intervention (*p* = 0.044). None of the other tests showed a statistically significant influence of the moderator variable: light intensity (*p* = 0.208), light color (*p* = 0.241), CS_t,f_ division (*p* = 0.543), intervention period/follow-up (*p* = 0.361) or study design (*p* = 0.105), whereas disease severity (*p* = 0.085) may be slightly more influencing (Supplementary Table S5). Notably, whether these factors yield a significant effect on the primary and secondary outcomes or not (see discussion in Section 4.2) could be dose-response confounding variables (see discussion in Section 4.3).

**Table 2:** Pre- to-post-treatment outcome sub grouped by study design, co-medication, and disease severity.

**Subgroup (study *n*)**	**Pooled SMD, random, 95% CI**	***z* test (*p*_1_)**	***I*^2^ (*p*_2_)**	***p* ^3^**	***p* ^4^**	***p* ^5^**	***p* ^6^**
**Subgroup A (31)**							
**Medicated (18)**	−2.10 (−2.50, −1.68)	−9.991(<0.01)	94.8% (<0.01)	0.01	0.05		
RCTs	−2.321 (−2.798, −1.845)	−9.543 (<0.01)	94.8% (<0.01)			0.000	0.000
Quasi experimental	−1.102 (−1.895, −0.310)	−2.726 (<0.01)	94.7% (<0.01)				
**Non-medicated (13)**	−0.799 (−1.010. −0.587)	−7.411 (<0.01)	70.6% (<0.01)	0.001	0.002		
RCTs (6)	−1.03 (−1.27, −0.78)	−8.283 (<0.01)	64.5% (<0.01)				
Non-depressed (7)	−0.398 (−0.700, −0.095)	−2.574 (<0.01)	60.9% (<0.01)				
**Subgroup B (24)**							
**Medicated (18)**	−2.10 (−2.50, −1.68)	−9.991(<0.01)	94.8% (<0.01)	0.135	0.29	0.000	0.009
Moderate to severe (13)	−2.215 (−2.683, −1.747)	−9.282 (<0.01)	95.5% (<0.01)				
Mild to moderate (5)	−1.536 (−2.293, −0.779)	−3.975 (<0.01)	87.2% (<0.01)				
**Non-medicated (6)**	−1.03 (−1.27, −0.78)	−8.283 (<0.01)	64.5% (<0.01)	0.502	0.45		
Moderate to severe (4)	−0.917 (−1.099, −0.734)	−9.833 (<0.01)	0.0% (0.597)				
Mild to moderate (2)	−1.137 (−1.752, −0.521)	−3.620 (<0.01)	88.1% (<0.01)				

Pre-to-post outcomes extracted at various time points within 24 depression-related studies; *p*1 is the *p*-value of the overall effect size test; *p*2 is the *p*-value within the subgroup referring to heterogeneity using Cochran's Q statistic; *p*3 is the *p*-value between severity subgroups using *Q*-statistic; *p*4 is the *p*-value between severity subgroups using *F-*statistic; *p*5 is the *p*-value between medication subgroups using *Q-*statistic; and *p*6 is the *p*-value between medication subgroups using *F-*statistic.

With co-medication suggested as a confounding factor, outcome data were further extracted within both co-medicated and non-medicated studies with only depressed participants. The meta-analysis (Table 2, Subgroup B) showed that 18 studies with co-medication (575 participants) obtained higher pre-to-post effect size (SMD = −2.1, 95% CI = −2.5 to −1.68; *z* = −9.991, *p* = 0.000; *I*^2^ = 94.8%) than those non-medicated (231 participants) (SMD = −1.03, 95% CI = −1.27 to −0.78; *z* = −8.283, *p* = 0.000, *I*^2^ = 64.5%), and the effect of this variable showed significant between-group heterogeneity [$\chi_{\left(1,85\right)}^{2}$ = 19.14, *p* = 0.000]. The outcome was simultaneously confirmed in conjunction with the F statistic [*F*_(1,83)_ = 7.08, *p* = 0.009].

To confirm the disease severity factor, a further subgroup meta-analysis of severity was undertaken. There was no significant between-group heterogeneity both with [$\chi_{\left(1,n=60\right)}^{2}$ = 2.24, *p* = 0.135] and without [$\chi_{\left(1,n=23\right)}^{2}$ = 0.45, *p* = 0.502] co-medication. Some inconsistency was observed in the subgroup with more severity—it yielded a numerically higher effect size (SMD = −2.215, 95% CI = −2.68 to −1.75; *z* = −9.282, *p* = 0.000; *I*^2^ = 95.5%) than a presumed milder subgroup (SMD = −1.53, 95% CI = −2.29 to −0.77; *z* = −3.975, *p* = 0.000; *I*^2^ = 87.2%) under co-medication conditions, whereas the reverse was observed in non-medicated studies.

### 4.2 Between-group efficacy comparison

Between-group efficacy comparison of circadian-active BLT vs. dim light control among RCTs was also studied with possible confounding factors such as visualization, co-medication, disease severity, as well as time pattern, which were all reported in the primary and secondary results.

#### 4.2.1 Visualization factors

**Figure 2:**
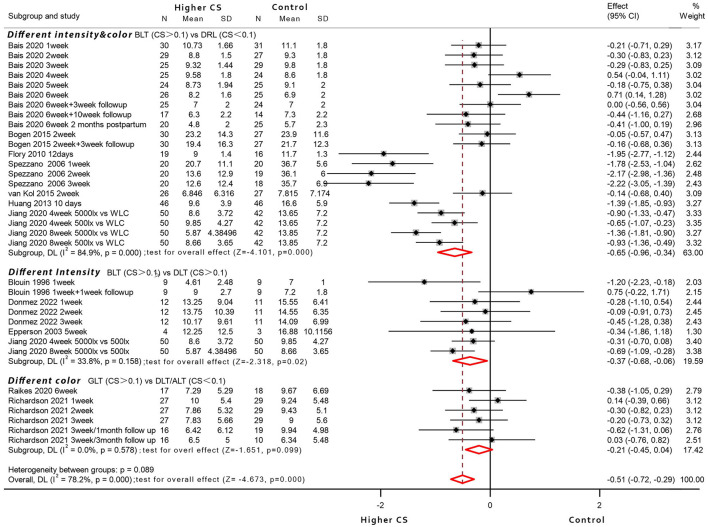
Forest plot displaying between-group meta-analysis of bright light therapy (higher circadian stimulus) vs. the control group (*n* = 12 articles; RCTs without identical baseline were excluded).

Assuming light intensity and light color across RCTs may exert visualization influence, they were examined as weighted random-effects meta-analyses (Figure 2), undertaking for each of the comparisons that were eligible for analysis, i.e., higher CS_t,f_ of BLT vs. lower CS_t,f_ of DLT- dim light therapy (four studies); higher CS_t,f_ of BLT vs. non-circadian dim red light-DRL (seven studies); and higher CS_t,f_ of GLT (green light therapy) vs. non-circadian DLT/amber light therapy-ALT (two studies). The intervention/control conditions were compared after parameter conversion (CS_t,f_ in the intervention group > CS_t,f_ in the control group).

Eleven studies had <100 lux dim-light control (light box or light visor), four studies utilized a sham negative ion generator with working lights and sound (81, 88, 90, 97), four studies adopted 500 lux light to eliminate placebo effect as much as possible (22, 30, 31, 71), and one study employed an untreated control arm (31). To conclude, ten studies where intervention could be visually distinguished among different arms were included as a subgroup, and so were the two RCT studies that adopted similar devices with identical photopic lux (76, 78). The participants were reported keeping as naive as possible regarding the existence and effects of LT (70, 71, 77, 81), or they held identical expectations for different interventions (70, 78, 88, 90, 97), or the light devices were identical in shape and appearance among double-blind studies (30, 78). However, the placebo effect could hardly be ruled out—it was proposed that the circadian stimulus from a non-visual perspective would exert additional influence; thus, the between-group comparison was based on higher/lower CS_t,f_ variation. Apart from exclusionary RCTs with unequal baseline [or not reported identical (68, 70, 79, 80, 82)], all others reported no statistically significant baseline difference and identical expectation of treatment response.

For the higher CS_t,f_ of GLT vs. non-circadian DLT/ALT (different color, two studies) subgroup, where LT devices shared similar appearance with equal photon densities of blue-green light vs. amber/red light, the results for a total of 91 participants were evaluated, of which 44 patients received blue-green light (CS_t,f_ > 0.1) and 47 patients received a placebo control light therapy (CS_t,f_ < 0.1). The meta-analysis with random-effects models found no evidence for the significant efficacy of GLT compared to amber/red light conditions (SMD = −0.21, 95% CI = −0.45 to 0.04; *z* = −1.651, *p* = 0.099; *I*^2^ = 0%).

For the higher CS_t,f_ of BLT vs. lower CS_t,f_ of DLT (different intensity of white light, four studies) subgroup where bright white light (*N* = 75 participants) compared with mainly 500 lux white light (*N* = 73 participants), the former yielded significant efficacy with pooled effect size of −0.37 (95% CI = −0.68 to −0.06; *z* = −2.318, *p* = 0.02; *I*^2^ = 33.8%) compared to controls (CS_t,f_ > 0.1 in both groups).

For higher CS_t,f_ of BLT vs. circadian-inactive DRL (different intensity and color, seven studies), the results for a total of 430 participants were evaluated, of which 221 patients received BLT (CS_t,f_ > 0.1), and 209 patients received a placebo control, presumably circadian-inactive (CS_t,f_ < 0.1). The results showed an overall high heterogeneity with a significant heterogeneity index when included trials were pooled (*I*^2^ = 84.9%) and revealed the bright light superiority in depressive symptoms reduction compared to the control group (pooled SMD = −0.65, 95% CI = −0.96 to −0.34; *z* = −4.101, *p* = 0.000).

The secondary outcome was also pooled on the random-effects inverse-variance model. Relative risk and 95% CIs were calculated for the subset of nine RCT studies of which the number of subjects who experienced response was known (Supplementary Figures 3A, B). The pooled estimate RR of BLT over no light placebo was 2.39 (95% CI = 1.54–3.73; *z* = 3.856, *p* = 0.000; *I*^2^ = 28.4%; three studies). For comparison over DLT and DRL controls, a revealed RR of 1.33 (95% CI = 0.98–1.80; *z* = 1.823, *p* = 0.068; *I*^2^ = 0.0%; four studies) and 1.53 (95% CI = 0.71–3.29; *z* = 1.085, *p* = 0.278; *I*^2^ = 0.0%; three studies) showed no significant superiority. Similarly, calculated odds ratios (95% CI) showed a significant advantage over no light control (OR = 9.59, 95% CI = 3.7–24.88; *z* = 4.648, *p* = 0.000; *I*^2^ = 70.3%) and DLT control (OR = 9.59, 95% CI = 3.7–24.88; *z* = 3.370, *p* = 0.001; *I*^2^ = 0.0%), but no significant difference of response rates between BLT and DRL control was found (pooled OR = 2.13, 95% CI = 0.76–5.99; *Z* = 1.434, *p* = 0.152; *I*^2^ = 12.3%). Overall, there was no significant difference between the three subgroups subdivided by visual characteristics [SMD: $\chi_{\left(2,35\right)}^{2}$ = 4.83, *p* = 0.09; RR: $\chi_{\left(2,13\right)}^{2}$ = 2.78, *p* = 0.25; OR: $\chi_{\left(2,13\right)}^{2}$ = 0.50, *p* = 0.78].

#### 4.2.2 Co-medication and disease severity factors

Since co-medication was largely a main heterogeneity source, in addition to the fact that response outcomes were barely reported by quasi-experimental trials, a subgroup meta-analysis was further carried out but focused on depression-oriented RCTs accompanied with or without co-medication (Supplementary Figures 3C, E). Where primary results indicated circadian-active BLT, the analysis showed significant efficacy compared to dimmer light controls under both co-medicated (pooled SMD = −0.47, 95% CI = −0.80 to −0.13; *z* = −2.749, *p* = 0.006; *I*^2^ = 82.0%; five studies) and non-medicated conditions (pooled SMD = −0.57, 95% CI = −0.81 to −0.33; *z* = −4.67, *p* = 0.000; *I*^2^ = 58.0%; four studies). There was no significant between-group heterogeneity caused by co-medication [$\chi_{\left(1,32\right)}^{2}$ = 0.24, *p* = 0.62].

Secondary outcomes indicated that circadian-active bright light showed significantly greater response likelihood than controls among both medicated (pooled RR = 1.56, 95% CI = 1.24–1.95; *z* = 3.869, *p* = 0.000; three studies) and non-medicated individuals (pooled RR = 6.31, 95% CI = 2.34–16.99; *z* = 3.645, *p* = 0.000; five studies). Both subgroups showed non-significant heterogeneity (*I*^2^ = 0.0%), indicating significant between-group heterogeneity caused by co-medication [$\chi_{\left(1,15\right)}^{2}$ = 8.45, *p* = 0.007]. Similar results of superiority could be drawn on a pooled estimate OR of 5.01 (95% CI = 2.79–8.99; *z* = 5.934, *p* = 0.000; eight studies) over controls. The noticeable superiority of response possibility may be due to co-medication that caused significant between-group heterogeneity [$\chi_{\left(1,15\right)}^{2}$ = 5.23, *p* = 0.004]. Both outcomes were further confirmed [co-medication RR: *F*_(1,13)_ = 8.24, *p* = 0.01; OR: *F*_(1,13)_ = 7.75, *p* = 0.01].

Response data was further extracted and subdivided by disease severity as a secondary subgroup. On the whole, consistent conclusions could be drawn from secondary outcomes among non-medicated studies (Supplementary Figure 3F), with a pooled RR value indicating circadian-active bright light showing overall superior response rate compared to controls, whether not significant under more severe conditions (pooled RR = 2.20, 95% CI = 0.86–5.63; *z* = 1.648, *p* = 0.099; *I*^2^ = 0.0%; three studies) or significant under milder conditions (pooled RR = 1.53, 95% CI = 1.21–1.92; *z* = 3.578, *p* = 0.000; *I*^2^ = 0.0%; two studies). Similar results were shown among co-medication studies (Supplementary Figure 3G), which showed significant superiority under more severe conditions (pooled RR = 6.29, 95% CI = 2.18–18.14; *z* = 3.405, *p* = 0.001; *I*^2^ = 0.0%; two studies) or non-significant under milder conditions (pooled RR = 6.43, 95% CI = 0.39–106.44; *z* = 1.299, *p* = 0.194; *I*^2^ = 0.0%; one study). There was no significant between-group heterogeneity among severity subgroups regarding response rates both with [$\chi_{\left(1,5\right)}^{2}$ = 0.01, *p* = 0.457] and without [$\chi_{\left(1,10\right)}^{2}$ = 0.42, *p* = 0.989] co-medication, confirming “co-medication” rather than “disease severity” was an efficacy comparison influential factor.

#### 4.2.3 Time pattern factors

The time factor (cumulative duration) that was implied as a heterogeneity source was further elaborated among depression-related RCTs. Primary results indicated circadian-active BLT showing significant or non-significant efficacy compared to dimmer light controls under various duration conditions (Supplementary Figure 3H), with combined results significantly indicating superior efficacy than the control group (pooled SMD = −0.49, 95% CI = −0.71 to −0.27; *z* = −4.34, *p* = 0.000; *I*^2^ = 77.7%; nine studies). There was no significant between-group heterogeneity caused by time pattern factor [$\chi_{\left(4,32\right)}^{2}$ = 9.03, *p* = 0.06].

Similar secondary pooled RR and OR estimates of eight RCTs indicated that the circadian-active bright light showed overall greater response likelihood than controls. There was no significant between-group heterogeneity caused by time [RR: $\chi_{\left(4,15\right)}^{2}$ = 6.72, *p* = 0.235; OR: $\chi_{\left(4,15\right)}^{2}$ = 7.88, *p* = 0.146], whether significant or not (Supplementary Figures 3I, J).

In conclusion, due to the non-ignoring visual distinction and the placebo effect, the efficacy of the bright light intervention compared to both circadian-inactive and active control conditions cannot be simply elucidated from the “circadian” perspective. Nevertheless, it seemed not all observed treatment responses were related to the non-specific effects since some cases had reported no relationship between expectation and improvement (88). On the whole, circadian-active BLT, whether significant or not, showed a greater possibility of response than active/inactive placebo, regardless of visualization, co-medication, disease severity, or cumulative duration that were assumed as confounding factors; co-medication alone showed more likelihood.

### 4.3 Dose-response relationship and influencing factors

**Figure 3:**
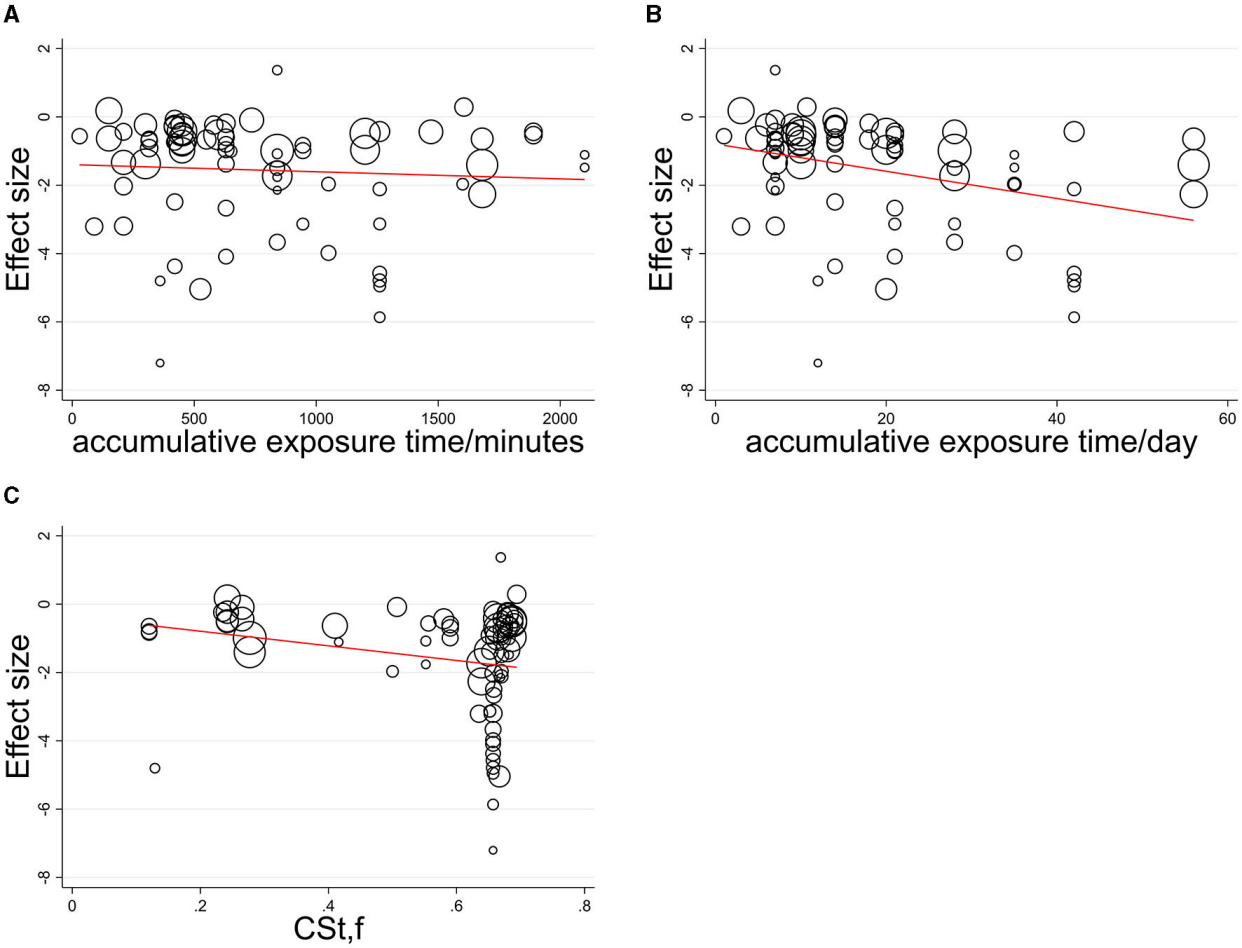
Meta-regression relationship between effect size and its standard error with **(A)** accumulative exposure min/h, **(B)** accumulative exposure days, **(C)** CS_t,f_ as a covariate.

A dose-response relationship was quantified with only vital continuous parameters such as CS_t,f_, and accumulative exposure time and was explored among the circadian studies (CS_t,f_ > 0.1; 31 studies, *N* = 813 participants). The single covariate was carried out in meta-regression to show the statistically significant influence of the moderator variable (Figure 3), indicating accumulative exposure min/h as irrelevant explanatory covariate (no explanation, *R*^2^ = 1.01%, *p* = 0.582) and relevant covariate as accumulative exposure days (some explanation, *R*^2^ = 12.43%, *p* = 0.003 < 0.05) and CS_t,f_ value (weak explanation, *R*^2^ = 5.71%, *p* = 0.041 < 0.05).

**Figure 4:**
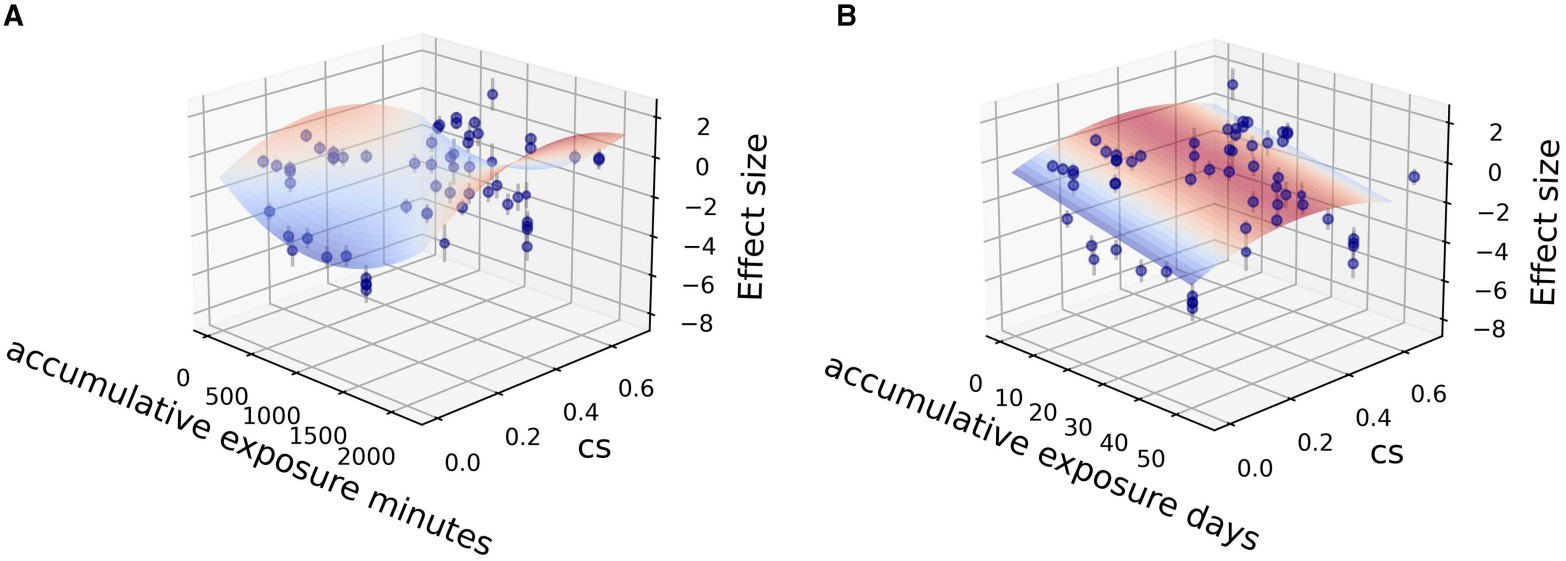
Fitting for data points clusters of 18 co-medication studies on poly 2D fitting models. **(A)** Poly 2D model fitted with accumulative exposure minutes, *z* = 1.0178 – 0.004*x* – 10.544*y* + 0.000002*x*^2^ – 15.33*y*^2^ + 0.00156*xy* (*R*^2^ = 6.07%). **(B)** Poly 2D model fitted with accumulative exposure days, *z* = −0.488 – 0.153*x* + 6.999*y* + 0.002*x*^2^ – 9.358*y*^2^ + 0.004*xy* (*R*^2^ = 17.0%).

**Figure 5:**
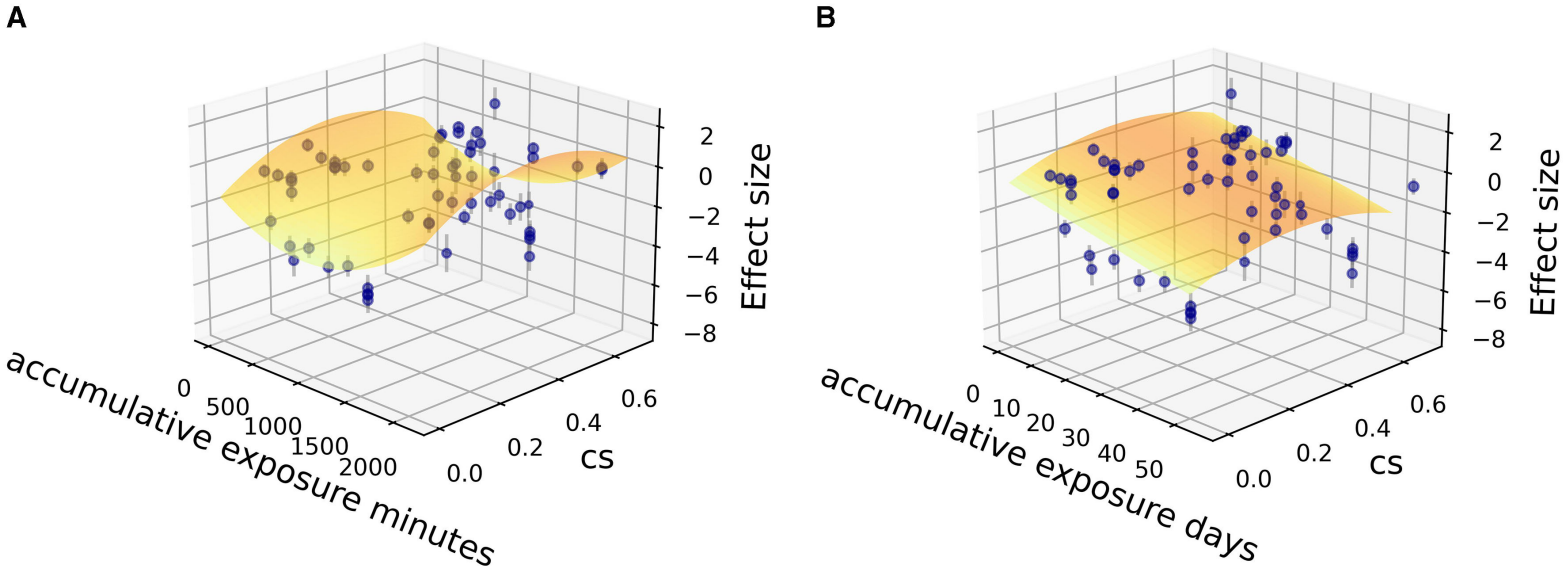
Fitting for data points clusters of 18 co-medication studies on parabola 2D fitting models. **(A)** Parabola 2D model fitted with accumulative exposure minutes, *z* = −1.50 – 0.00368*x* + 11.78*y* + 0.00000195 *x*^2^ – 15.31*y*^2^ (*R*^2^ = 4.59%). **(B)** Parabola 2D model fitted with accumulative exposure days, *z* = – 0.52 – 0.15*x* + 7.127*y* + 0.0021 *x*^2^ – 9.421 *y*^2^ (*R*^2^ = 17.0%).

**Figure 6:**
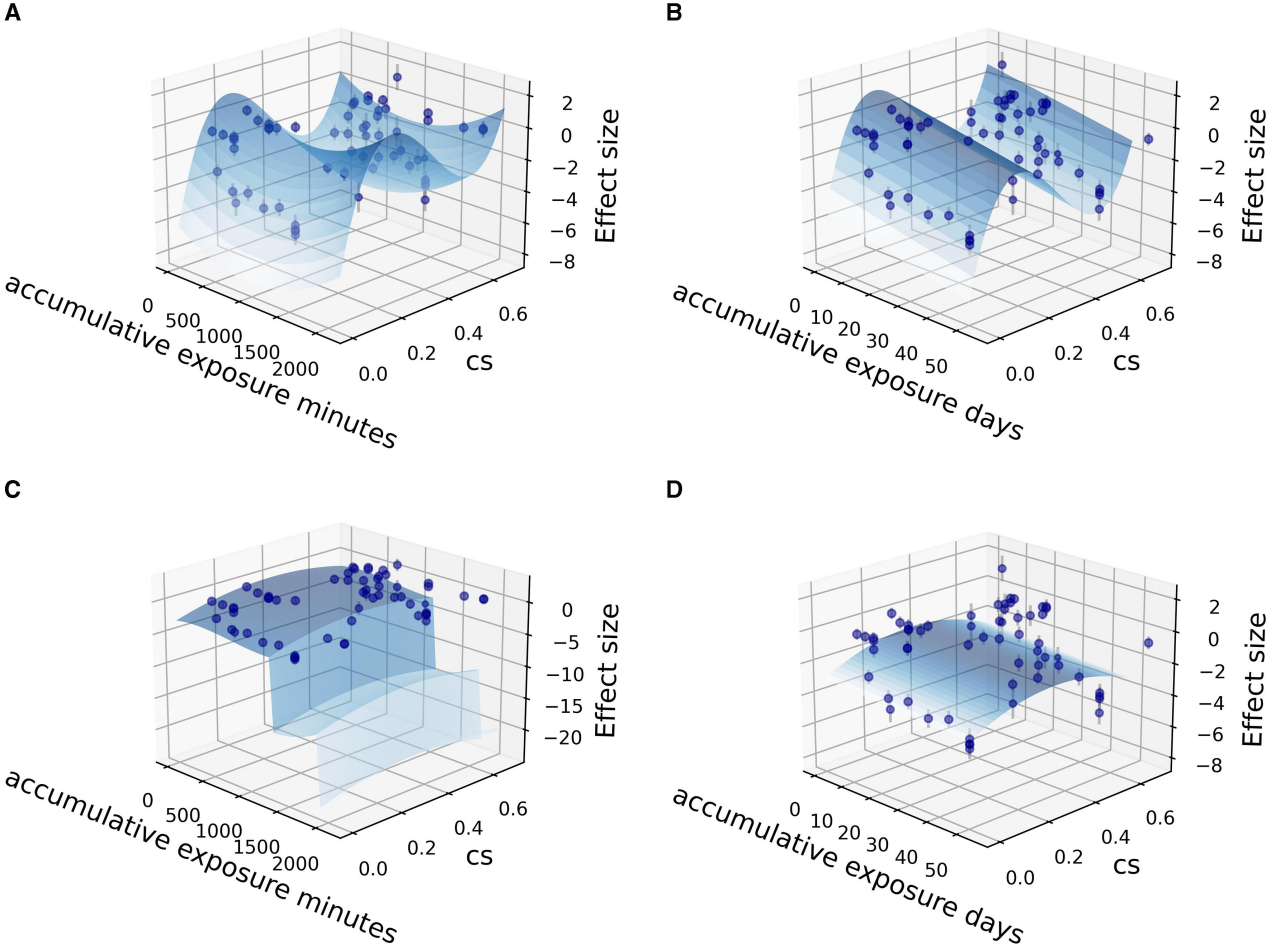
Fitting for data points clusters of 18 co-medication studies on polynormal 2D fitting models. **(A)** Polynormal 2D model fitted with accumulative exposure minutes, *z* = 6.211–0.0037*x* + 0.000975*x*^2^ + 80.506*y* + 0.00000188*x*^2^ – 265.733*y*^2^ + 236.39*y*^3^ (*R*^2^ = 15.62%). **(B)** Polynormal 2D model fitted with accumulative exposure days, *z* = −5.349 – 0.1556*x* + 0.002*x*^2^ + 75.99*y* + 0.00000188*x*^2^ – 258.30*y*^2^ + 233.96*y*^3^ (*R*^2^ = 28.0%). **(C)** Polynormal 2D model fitted with accumulative exposure minutes (–). **(D)** Polynormal 2D model fitted with accumulative exposure days, z = 2.933 + 0.306*x* – 0.0185*x*^2^ + 0.000243*x*^3^ + 8.649*y* – 11.993*y*^2^ (*R*^2^ = 33.76%).

Fitting was further carried out by two independent variables (CS_t,f_, time) among the sub grouped medicated and non-medicated studies to show their contribution to therapeutic effect size, utilizing the Levenberg Marquardt algorithm. The 3D fitting outcomes among medicated patients (Figures 4–6) offer a glimpse of the relationship between accumulative circadian stimulus and malady reduction. Based on current data, the fitting adaptability of accumulative exposure days (*R*^2^ = 17.0–33.7%) was overall better than minutes (*R*^2^ = 4.59–12.62%). Meanwhile, accumulative circadian stimulus, i.e., *P(u)* illustrated by *T*_(*u*)_ and CS_t,f_ may largely be explained by polynomial models with better goodness of fit that shown in Equations 4 and 5:


(4) $$\begin{array}{l} \text{P}\;\left(\text{u}\right)\;=\;\text{a}+\;\text{b}\;\times \;\text{T}\;+\;\text{c}\;\times {\text{T}}^{2}\;+\;\text{d}\;\times {\text{T}}^{3}+\;\text{e}\;\times {\text{C}\text{S}}_{\text{t},\text{f}}\;+\;\text{f}\;\times \;{{\text{C}\text{S}}_{\text{t},\text{f}}}^{2} \end{array}$$

or


(5) $$\begin{array}{l} \text{P}\left(\text{u}\right)=\text{a}+\text{b}\times \text{T}+\text{c}\times {\text{T}}^{2}+\text{d}\times \text{C}{\text{S}}_{\text{t},\text{f}}+\text{e}\times \text{C}{\text{S}}_{\text{t},\text{f}}{\;}^{2}+\text{f}\times \text{C}{\text{S}}_{\text{t},\text{f}}{\;}^{3} \end{array}$$

where a, b, c, d, e, f are all parameters working on the slope and direction of the curve. T represents accumulative exposure time. And the models implied that the therapeutic effect may reach saturation.

The overall dose-response relationship among depressed, co-medicated patients (18 studies) implied saturation would reach at about 36 days (1,000 min) modeled on both poly 2D parabora2D equations and 34.4 days modeled on polynormal 2D models.

**Figure 7:**
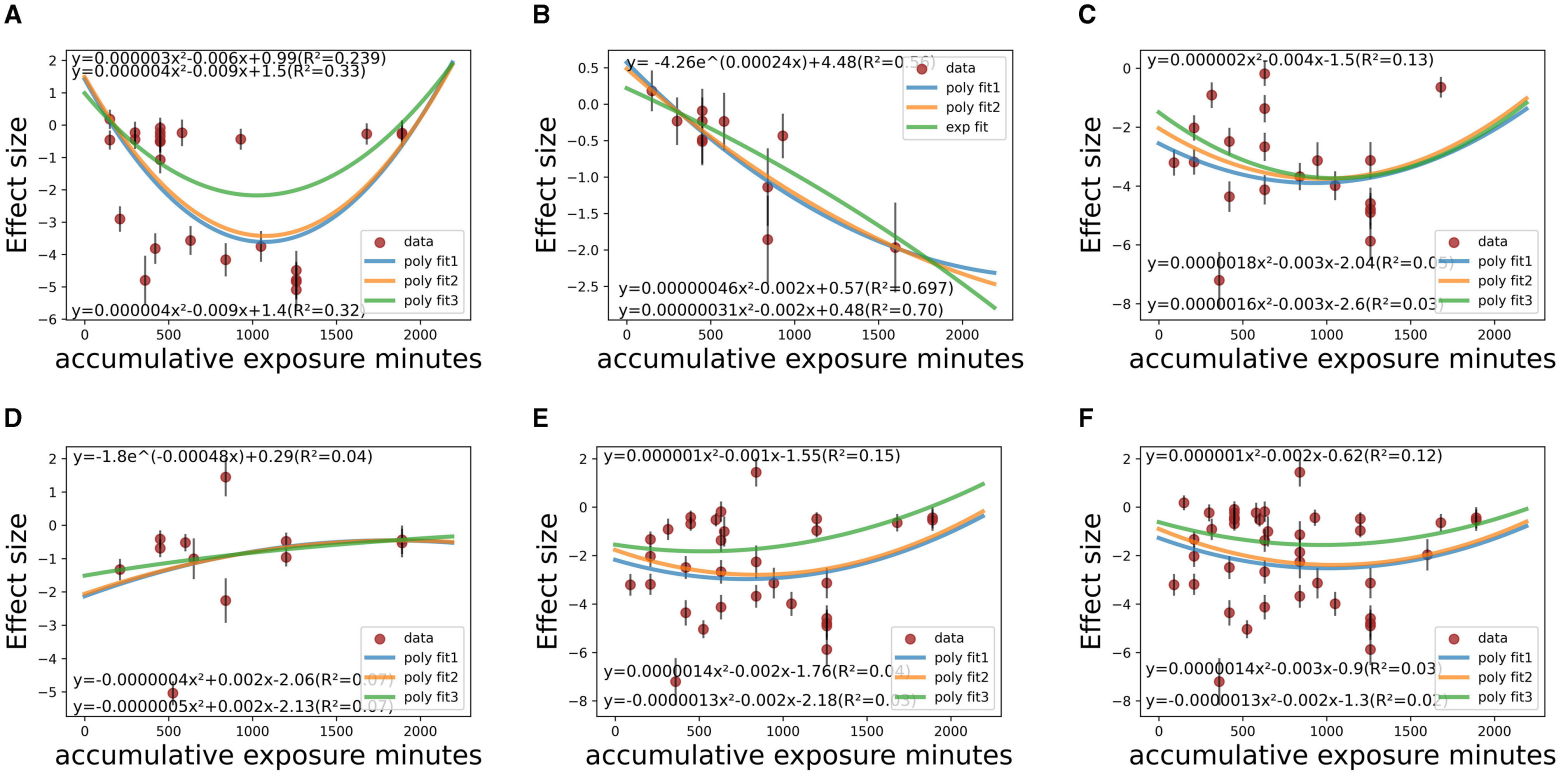
Temporal fitting (accumulative e*x*posure minutes) among co-medicated studies within various CS_t,f_ ranges. **(A)** CS_t,f_ range < 0.1–0.4 (*R*^2^ = 23.9–33.0%). **(B)** CS_t,f_ range 0.40–0.60 (*R*^2^ = 28.2–30.8%). **(C)** CS_t,f_ range 0.60–0.665 (*R*^2^ = 1.0–13%). **(D)** CS_t,f_ range 0.665–0.7 (*R*^2^ = 4.0–7.0%). **(E)** CS_t,f_ range 0.60–0.70 (*R*^2^ = 3.0–15.0%). **(F)** CS_t,f_ range 0.20–0.70 (*R*^2^ = 2.0–12.0%).

**Figure 8:**
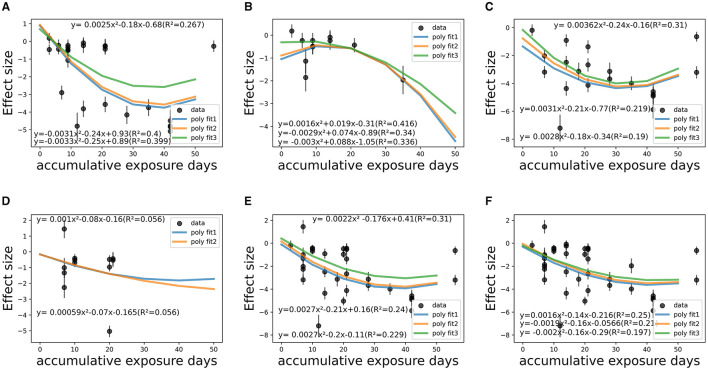
Temporal fitting (accumulative exposure days) among co-medicated studies within various CS_t,f_ ranges. **(A)** CS_t,f_ range < 0.1–0.40 (*R*^2^ = 26.7–40.0%). **(B)** CS_t,f_ range 0.40–0.60 (*R*^2^ = 33.6–41.6%). **(C)** CS_t,f_ range 0.60–0.665 (*R*^2^ = 19.0–31.0%). **(D)** CS_t,f_ range 0.665–0.70 (*R*^2^ = 5.6%). **(E)** CS_t,f_ range 0.60–0.70 (*R*^2^ = 22.9–31.0%). **(F)** CS_t,f_ range 0.20–0.70 (*R*^2^ = 19.7–25.0%).

Figures 7, 8 show that the dose-response relationship between therapeutic effect size (SMD value) and its standard error with accumulative exposure time as an independent variable in various CS_t,f_ ranges fit well with the exponent function or polynomial function. Among medicated individuals, The effect size would reach saturation in about 1,000 min/36–38 days in CS_t,f_ < 0.1–0.4 range (*n* = 7 studies, 26 items), 900–1,000 min/32–33 days in 0.6–0.665 range (*n* = 7 studies, 20 items), and 40 days in 0.665–0.7 (*n* = 7 studies, 13 items) ranges (780–850 min/37–38 days in 0.6–0.7 range, 970–1,000 min/41–42 days in 0.2–0.7 range). On the whole, in most “circadian” conditions (0.2 < CS_t,f_ < 0.7), polynomial models implied the saturation would reach 900–1,000 min (32–42 days) as temporal saturation for medicated AYAs.

Similar quantification outcomes were checked in non-medicated and non-depressed people (Supplementary Figures 5, 6) and severity × co-medication interaction tests (Supplementary Figures 7, 8). For non-medicated, depressed people (six studies), it was found that saturation would reach in ~1,350 min (accumulative) fitted by the poly 2D model and 1,450 min (58.9 days) by the parabora2D equations. The polynomial models implied 1,100–1,500 min as temporal saturation for non-medicated AYAs. Similar outcomes were verified among those who suffered at least moderate disease severity, where 700–1,000 min (22–41 days) saturation was implied with co-medication and 700–1,500 min for the non-medicated subgroup, indicating the dominant synergistic effect of medication. In contrast, among those with mainly mild depression (seven studies) or non-depressed (seven studies), the saturation seemed indistinct. The confounding effect of disease severity was not qualified due to limited samples.

For circadian RCT studies (CS_t,f_ > 0.1), the implied saturation reached about 1,000 min (46 days) based on the poly2D model, 1,145 min (44 days) on the parabora2D model, and 1,230 min (44 days) modeled on the polynormal 2D equation. No quantitative conclusions were derived among quasi-experimental studies. Whether the study design was influencing remains ambiguous (Supplementary Figure 9).

On the whole, CS_t,f_, duration, and co-medication proved to be dose-response influencing factors, whereas the potential confounding effects of disease severity, study design, and lighting administration are yet to be fully explicated (Supplementary Figures 4, 10). Overall, 1,000–1,500 min (~30–60 days) of accumulative duration can be inferred as saturation, as fully discussed with certain factors. Meanwhile, it is concluded that accumulative duration *T*_(*u*)_ suitably fitted into the polynomial model within various CS_t,f_ intervals, and the relationship did not change significantly after adjusting for various confounding factors (equation 6), that:


(6) $$\begin{array}{l} {\text{P}}_{\left(\text{u}\right)}\;=\;\text{a}\;+\;\text{b}\;\times \;\text{T}\;+\;\text{c}\;\times {\text{T}}^{2} \end{array}$$

### 4.4 Saturation of light therapy

Since continuous dose-response analysis showed a non-linear relationship between temporal pattern and depression reduction, subgroup meta-analysis of temporal pattern within CS_t,f_ variation was further specified, considering co-medication as a covariate. Depression reduction showed in pooled estimates of SMD was associated with cumulative duration intervals (subdivided by 5, 35, 65, 95%, i.e., dose division). The categorical dose-response analysis was undertaken by comparing 0–300 min/300–500 min/500–1,000 min/1,000–1,500 min/>1,500 min subgroups with each other, using random-effects modeling techniques, and *p*-values < 0.05 were considered statistically significant with all double-sided testing (Table 3).

**Table 3:** Significant between-group differences are sub grouped by main heterogeneity sources (CS_t,f_, co-medication, and temporal pattern).

**Subgroup (study *n*)**	**Temporal pattern/mins**	**Pooled SMD, random, 95% CI**	***z* test (*p*_1_)**	***I*^2^ (*p*_2_)**	***p* ^3^**	***p* ^4^**	***p* ^5^**
**CS**_t,f_<**0.2**							
Medicated (4)	0–300	−1.25 (−2.66, 0.16)	−5.869 (<0.01)	94.5% (<0.01)	<0.01	0.006	0.007
	300–500	−2.01 (−3.43, −0.59)		94.7% (<0.01)			
	500–1,000	−3.80 (−4.30, −3.29)		0% (0.639)			
	1,000–1,500	−4.81 (−5.34, −4.27)		0% (0.902)			
	>1,500	−0.26 (−0.77, 0.24)		0% (0.943)			
Non-medicated (2)	0–300	−0.65 (−1.51, 0.22)	−5.190 (<0.01)	0 (1.0)	0.922	NA	
	300–500	−0.84 (−1.23, −0.44)		0% (0.66)			
	500–1,000	−0.81 (−1.43, −0.19)		0% 0.966)			
	>1,500	−0.48 (−1.20, 0.25)		0 (1.0)			
Non-depressed, non-medicated (4)	0–300	0.15 (−1.15, 1.44)	−0.173 (0.863)	83.1% (0.015)	0.688	NA	
	500–1,000	−0.34 (−1.08, 0.41)		0 (1.0)			
	1,000–1,500	0.06 (−0.59, 0.71)		0 (1.0)			
**0.2**<**CS**_t,f_<**0.4**							
Medicated (4)	0–300	0.15 (−1.15, 1.44)	−2.253 (0.024)	0% (0.424)	0.729	NA	0.02
	500–1,000	−0.34 (−1.08, 0.41)		0% (0.759)			
	1,000–1,500	0.06 (−0.59, 0.71)		0 (1.0)			
Non-medicated (1)	500–1,000	−0.99 (−1.40, −0.57)	−5.709 (<0.01)	0 (1.0)	0.175	NA	
	>1,500	−1.40 (−1.84, −0.97)		0 (1.0)			
**0.4**<**CS**_t,f_<**0.6**							
Medicated (2)	500–1,000	−1.45 (−2.21, −0.69)	−4.911 (<0.01)	0% (0.358)	0.473	NA	0.04
	>1,500	−1.97 (−3.15, −0.79)		0 (1.0)			
Non-medicated (2)	300–500	−2.01 (−3.43, −0.59)	−3.311 (<0.01)	0 (1.0)	0.904	NA	
	500–1,000	−3.80 (−4.30, −3.29)		0% (0.519)			
	>1,500	−0.48 (−1.20, 0.25)		0 (1.0)			
Non-depressed, non-medicated (4)	0–300	−0.61 (−1.08, −0.15)	−2.590 (0.01)	0% (0.901)	0.475	NA	
	300–500	−0.08 (−0.80, 0.64)		0 (1.0)			
	1,000–1,500	-−0.43 (−1.11, 0.25)		0 (1.0)			
**0.6**<**CS**_t,f_<**0.7**							
Medicated (15)	0–300	−2,12 (−3.03, −1.21)	−7.945 (<0.01)	87.2% (<0.01)	<0.01	0.26	0.037
	300–500	−2.01 (−4.98, −0.97)		97.0% (<0.01)			
	500–1,000	−2.23 (−3.29, −1.18)		95.3% (<0.01)			
	1,000–1,500	−3.48 (−5.10, −1.87)		96.6% (<0.01)			
	>1,500	−0.55 (−0.97, −0.14)		0% (0.918)			
Non-medicated (5)	0–300	−1.25 (−2.66, 0.16)	−5.209 (<0.01)	0 (1.0)	<0.01	0.53	
	300–500	−2.01 (−3.43, −0.59)		0% (0.824)			
	500–1,000	−3.80 (−4.30, −3.29)		0.5% (0.389)			
	1,000–1,500	−4.81 (−5.34, −4.27)		0 (1.0)			
	>1,500	−0.26 (−0.77, 0.24)		93.5% (<0.01)			
Non-depressed, non-medicated (3)	300–500	−0.67 (−1.45, 0.11)	−2.264 (0.024)	82.1% (<0.01)	0.986	NA	
	500–1,000	−0.66 (−1.38, 0.05)		0 (1.0)			

*p*1 is the *p*-value of the overall effect size test; *p*2 is the *p*-value within the subgroup referring to heterogeneity using Cochran's *Q*-statistic; *p*3 is the *p*-value between temporal subgroups referring to heterogeneity using *Q-*statistic; *p*4 is the *p*-value between time pattern groups using *F*-statistic; and *p*5 is the *p*-value between medication-depression groups using *F*-statistic.

In the CS_t,f_ < 0.1–0.2 range that is barely considered as “circadian” condition, there was significant pre- to post-treatment difference between 1,000–1,500 min/>1,500 min (*p* = 0.0018), 1,000–1,500 min/0–300 min (*p* = 0.004), 1,000–1,500 min/300–500 min (*p* = 0.008), and 500–1,000 min/0–300 min (*p* = 0.03), 500–1,000 min/>1,500 min (*p* = 0.01) subgroups among depressed and medicated AYAs, indicating time as a confounding factor [*F*_(4,12)_ = 6.06, *p* = 0.006]. There was no significant between-group heterogeneity among depressed but non-medicated persons (*p* = 0.922), nor among non-depressed, non-medicated people (*p* = 0.688). In contrast, there was a significant difference between the three subgroups [*F*_(2,24)_ = 5.99, *p* = 0.007].

In the CS_t,f_ 0.2–0.4 range, there was no significant between-group heterogeneity of time pattern among depressed and medicated patients (*p* = 0.729) or non-medicated people (*p* = 0.175). No significant pre- to post-treatment difference was observed between various duration subgroups [*F*_(2,5)_ = 0.33, *p* = 0.73]. In contrast, a significant difference existed between with/without co-medication subgroups [*F*_(2,8)_ = 15.01, *p* = 0.002].

In the CS_t,f_ 0.4–0.6 range, there was no significant between-group heterogeneity of time pattern among medicated patients (*p* = 0.473), depressed but non-medicated patients (*p* = 0.904), and non-depressed, non-medicated people (*p* = 0.475). In contrast, a significant difference existed between these three groups [*F*_(2,8)_ = 4.55, *p* = 0.04].

In the CS_t,f_ 0.6–0.7 range among the depressed but non-medicated people, there was significant between-group heterogeneity (*p* = 0.004 < 0.5), but it could not be explained by between-group differentiation due to high heterogeneity within subgroups. No significant difference was found between temporal pattern groups [*F*_(4,7)_ = 0.87, *p* = 0.53]. In the CS_t,f_ 0.6–0.7 range among depressed, medicated people, no significant pre to post-treatment difference was found between time pattern differentiation [*F*_(4,29)_ = 1.39, *p* = 0.26]. However, there was a significant difference between 1,000 and 1,500 min/>1,500 min subgroups (*p* = 0.03 < 0.05). Similarly, a significant difference was observed between the three groups [*F*_(2,45)_ = 3.52, *p* = 0.037].

In conclusion, 1,000–1,500 min of accumulative exposure duration is suggested as a threshold, especially on a higher CS_t,f_ basis, and co-medication was verified as the main heterogeneity source, corresponding with previous outcomes.

### 4.5 Publication bias

Using Egger's linear regression test, we found there existed publication bias and small study effects (Supplementary Figure 11) with all outcomes (97 items, intercept = −6.25, 95% CI = −8.52 to −3.99, *t* = 5.48, *p* = 0.000 < 0.05), only “circadian” studies (73 items, intercept = −4.82, 95% CI = −7.23 to −2.41, *t* = 3.98, *p* = 0.000 < 0.05), only “depression” studies (85 items, intercept = −7.27, 95% CI = −9.69 to −4.86, *t* = 5.99, *p* = 0.000 < 0.05), only co-medicated studies (61 items, intercept = −10.75, 95% CI = −13.62 to −7.88, *t* = 7.49, *p* = 0.000 < 0.05), as well as only RCT studies (85 items, intercept = −7.20, 95% CI = −9.61 to −4.78, *t* = 5.94, *p* = 0.000 < 0.05), respectively. However, for the purpose of dose-response quantification, the more data was included, the better.

## 5 Discussion

### 5.1 Efficacy of specified, quantified circadian light therapy

To our knowledge, the study may not be the first systematic review of BLT on youth, but it is the first meta-analysis of lighting therapy focusing on circadian stimulus and its accumulative dose-response on depression-related illnesses for AYAs. On the whole, bright light therapy for depressed AYA with higher CS_t,f_ cannot be proved significantly efficacious over lower CS_t,f_ light interventions since symptom reduction was seen in both groups. It was largely influenced by both circadian ways and visual ways, accompanied by the fact that strong circadian evidence has not been found in young individuals with severe visual impairment or blindness (capacity for photoentrainment may be sustained) since empirical evidence was only derived from certain adults (98). However, measuring circadian timing in future trials would allow for a more rigorous examination of mechanisms (and possibly different pathways) linking circadian misfunction with depressive symptomology.

To explore circadian stimulus connection with therapeutic efficacy, CS_t,f_ can indeed be used as a metric quantification method for its accuracy in circadian phototransduction process in AYA depression-oriented clinical trials and theoretical studies. The conclusion was supported and validates previous conclusions quantitatively in the following ways. (1) It can be conservatively concluded that when exerting light exposure with certain circadian stimulus (CS_t,f_ = < 0.1–0.7), 30–2,100 min of accumulative time (roughly within 8 weeks) is efficacious for disease amelioration. Despite the value of *I*^2^ (*I*^2^ = 92.8%) indicating a high degree of heterogeneity, the pooled SMD values of the vast majority of studies indicated at least small (>0.2) to large (>2.0) change of effect size. The result has supported a broader range of CS_t,f_ of light therapy compared to previous conclusions where CS_t,f_ ranged from 0.57 to 0.7 (1).

(2) The therapeutic effect has shown a positive relationship with increasing light dose in both within-group and crossover changes. For young people primarily aged <32 (approximate mean age 22.3 ± 7.4), temporal duration of exposure contributed up to about 20–30% (or much higher) to within-group effect size variation (fitted by various models), while the therapeutic effect size was less be explained by CS_t,f_ (*R*^2^ = 5.71%), co-medication (*R*^2^ = 6.94%) or hardly by other confounding factors, indicating that overall temporal pattern was the most crucial. These quantified conclusions have been drawn from regression models performed by Statas 17.0, CMA 3.0, and Python 3.9 that polynomial 2D models can better illustrate quantification correlation between therapeutic effect and accumulative circadian stimulus, despite fitting models showing imperfection statistically (much-oscillated *R*^2^).

(3) Dose-response saturation. From the dose-response fitting and subgroup meta-analysis of temporal patterns, accumulative 900–1,000 min (32–42 days) of duration may be the saturation for depressed and medicated AYAs and 1,100–1,500 min (58–59 days) for non-medicated patients. Albeit, 1,000–1,500 min (5–7 weeks) of accumulative exposure duration showed more efficacy in symptom reduction than <1,000 min (3–4 weeks) or >1,500 min (7+ weeks) subgroups within high circadian stimulus (0.6 < CS_t,f_ < 0.7). For CS_t,f_ < 0.2 intervals that are barely considered as “circadian” conditions, accumulative 500–1,000 min duration may be the most efficacious among depressed and medicated AYAs. Meanwhile, for non-depressed individuals, the temporal pattern could not be verified due to limited samples. The results suggest that for common LT devices (LT-box, lamps, glasses), 1,000–1,500 min (5–7 weeks) of the threshold may be saturation combined with medication, regardless of their lighting features (e.g., light levels, spectra, light distribution). This conclusion endorses and expands previous conclusions, suggesting 2–5 weeks of exposure (16).

(4) Possible polynomial models on accumulative circadian stimulus and therapeutic effect have been quantified beyond consecutive light dose (CS_t,f_ value), which has not been illustrated in previous studies.

### 5.2 Heterogeneity and clinical efficacy discussion

Discussion on clinical efficacy with regard to PICO principles: (1) Participants. Although the subjects discussed were all adolescents and youth, their depression episodes, phenotype, severity, light exposure history, and co-medication status may have caused differentiation and heterogeneity. From another perspective, there is preliminary evidence with regard to various circadian-related illnesses where light therapy has shown improvement [e.g., bipolar depression (99), atypical depression (100), melancholic depression (100), unipolar depression (101), light therapy with more accumulated circadian stimulus may be an efficacious treatment for “circadian” depression (102)], where conventional pharmacological intervention had poor responses. Indeed, AYAs have benefited from light therapy as an adjunctive, additive, and non-invasive treatment to their continued treatment modalities despite uncertainties and difficulties. In this study, the discrepancy may partially be explained by demonstrated resistance to pharmacotherapy (83) or depression severity [mild depression might coexist (62, 67, 76, 81, 82, 92)], whilst compliance and adverse side effect did not appear to be the confounding factors.

As indicated, bipolar depression, major depressive disorder, postpartum depression, subthreshold depression, and dysthymia may share and respond to similar lighting therapeutic mechanisms. However, as not yet extensively investigated, the presence of certain comorbid disorders may compromise treatment efficacy, e.g., whether seasonality or comorbid SAD increases the likelihood of positive response to light (103) as reported included (22, 71, 72, 74, 88, 97), or Axis *I* anxiety disorders (90) and Axis II personality disorders vice versa (72). For most non-comorbid cases, a reduction in disease severity had been observed (30, 31, 70, 78). On one hand, it is necessary to identify homogenous patient groups. On the other hand, we still emphasize the vital role of dosing. As had been implied, emerging hypomanic symptoms may be relieved after a small increment in exposure duration (22), but qualitative discussion on comorbidity alone may be far from sufficient.

(2) Intervention perspective. Only three studies excluded any form of intervention (medication, psychotherapy, etc.) within at least the past 6 months (30, 31, 75). Three studies reported no medication (22, 82, 87). A few studies reported no recently initiated antidepressants or the use of psychotropic medication had remained stable (68, 70–72, 74, 88). Several studies excluded light-sensitizing medication that may act as photosensitizers and increase the risk of eye/skin damage (62, 74). Additional interventions were generally balanced between experiment vs. control groups, and participants from both groups had received identical medication/psychotherapy, if applicable (64). A few studies reported medication had little or no effect on the overall result (66, 97). However, it is scarcely possible that the evaluation of light therapy on mood eliminated a potentially confounding variable of medication. Light therapy has been reported with a clear synergistic effect when combined with SSRIs (selective serotonin reuptake inhibitors) during a moderate to severe major depressive episode (4). Though medication components were barely reported by included studies, significant differences between with and without co-medication were seen not only in between-group comparison but also in pre-to-post-effect size and dose-response relationship, although it was impossible to completely separate the effects of co-medication from depression severity. Additionally, it is less possible that medication masks the effects of light therapy since the outcomes of LT monotherapy were found to be equivalent or superior to that of the medicated group on a lower CS_t,f_ basis (Table 3).

(3) Experimental design. In this study, both RCTs and non-randomized experimental studies were included for therapeutic efficacy evaluation, while few previous studies have discussed the aspect by merely including RCT studies (3) since total blindness for RCTs is quite hard to achieve, as mentioned. Since intuitive, neurophysiological, and chronobiological light therapies are distinct from pharmaceutical interventions, we deduced less differentiation caused by design methodology and combined overall outcomes by fully discussing between-group differences and comparing dose-response saturation. Moreover, the heterogeneity could be influenced by in-, out- patients or whether they adopted home-based protocol, since those administered in laboratory or hospital treatment rooms where the protocol may largely be correctly followed had implied more eligibility than those less-supervised home-based evidence.

(4) Statistics. Although SMD effect size and random-effect model were applied for collected data, depression measurement outcomes with different scales may have led to certain heterogeneity.

## 6 Limitation

The study has several limitations. On the whole, there were limited samples since only a few studies focused on AYA-oriented depression light therapy with mainly small samples. In order to elaborate on accumulative light stimulus and reductions in relevant maladies, 31 articles (*N* = 1,031) included not only diagnosed depressed individuals but also healthy participants for circadian improvement intentions. Since the adopted outcomes were SMD mean values rather than individual results, the meta-regression may result in aggregation bias. The outcomes of fitting accumulative exposure time and CS_t,f_ as two independent variables indicated imperfection in mathematics. However, the results had been adjusted and intercalibrated with subgroup meta-analysis and have implied trend and saturation of accumulative light dose.

The limitations based on CS_t,f_ model, are as follows: (1) Despite that CS_t,f_ values ranged from <0.1–0.7, it is scarcely possible that continuous CS_t,f_ values can be acquired. Subgroup meta-analysis and meta-regression have been carried out on this basis, by which the accuracy of the fitting models was also influenced by restricted CS_t,f_ values. (2) Spatial distribution. The circadian light spatial distribution factor *f* and intensity have not been totally validated. Parameters like the distance, the angle between the lighting device and human eyes, light source positions (104), and background reflection factors were not provided. Therefore, CS_t,f_ factor was not well-discussed without details. (3) Intervention moment factor may also be influential. Some studies (105, 106) have quantified light moments in circadian phase shift and DLMO calculation. However, since only one included study (83) carried out morning and night BLT, four studies carried out nocturnal BLT (63, 71, 80, 81). In some studies, subjects were relatively flexible in receiving BLT at home; further validation is needed for the quantification. The quantified model could be explored in future studies with larger samples and specific individual results.

At present, there is only a CS_t,f_ model in discussion; other light dose-related responses through light-sensitive circuits have not yet been explored. More targeted phototherapy studies on depression-related light-sensitive circuits on patients with different depression phenotypes, severity, light exposure history, physiological characteristics, gender as well and exposure duration are necessary for therapeutic efficacy validation. Moreover, light therapies and correspondent circadian stimulus for combined treatment (e.g., antidepressants, chronotherapy) should be explored with consistent clinical trials and follow-ups. In addition to larger, all-around samples and precise experimental design for heterogeneity reduction, more objective parameters and indicators are necessary for efficacy evaluation beyond standardized depression measurement outcomes. Objective evaluation methods and approaches like neuron-related blood inflammatory markers (107), cerebrospinal fluid (CSF) analysis (108), or brain physiological examinations electroencephalogram/EEG (109) can also be adopted as evaluation tools when possible.

## 7 Conclusion

The significant efficacy of a higher circadian stimulus of light therapy over a lower circadian stimulus of light intervention among AYAs remains unproven. Yet, factors such as co-medication, disease severity, time pattern, visual characteristics, etc., are considered sources of heterogeneity that affect the response potential. Conservatively, light therapy with certain circadian stimuli has indicated significant reductions in relevant maladies both among medicated (pooled SMD = −2.1, 95% CI = −2.51 to −1.68; *z* = −9.979, *p* = 0.000; *I*^2^ = 94.8%) as well as non-medicated persons (pooled SMD = −1.03, 95% CI = −1.27 to −0.78; *z* = −8.283, *p* = 0.000; *I*^2^ = 64.5%), with enhanced response superiority through co-medication. The dose-response relationship between accumulative circadian stimulus (considered as light dose for the circadian system) and disease reduction has been specified by meta-regression and dose-response quantification based on CL_A_ and CS_t,f_ models, indicating accumulative 32–58 days (1,000–1,500 min) as saturation, considering co-medication, severity, study design, etc., are all dose-response influencing factors. It is advised that for the treatment of depression in adolescents and young adults, using current common light therapy devices for “circadian” light therapy (0.1 < CS_t,f_ < 0.7), an accumulative duration of 1,000–1,500 min (5–7 weeks/32–58 days) may be effective. However, for co-medicated patients, the effect size may reach saturation in about 900–1,000 min (32–42 days), while for non-medicated, depressed individuals, it may take 1,100–1,500 min (48–58 days) to reach saturation. It is also possible that an accumulative duration of more than 1,500 min may not be as efficacious on a high CS_t,f_ basis. Overall, the study has provided quantified references for light patterns and neural responses that are vital in the neuropsychological mechanism of light intervention, as well as guidance for clinical application.

## Author contributions

RC: Conceptualization, Data curation, Methodology, Software, Writing – original draft, Writing – review & editing. YY: Conceptualization, Supervision, Writing – original draft, Writing – review & editing. XC: Data curation, Writing – original draft, Writing – review & editing.
