# Supplementary material for: Circadian light therapy and light dose for depressed young people: a systematic review and meta-analysis
Source: Front Public Health. 2024 Jan 8;11:1257093. doi: 10.3389/fpubh.2023.1257093 (PMC10800803; doi:10.3389/fpubh.2023.1257093)
Supplement: Supplementary file 1 [file Data_Sheet_1.docx]

Supplementary Material

# Table S1. Search strategy for the meta-analysis

| Database | Search Terms | Results |
| --- | --- | --- |
| Embase |  | 237 |
|  | ('juvenile'/exp OR 'juvenile' OR 'youth'/exp OR 'youth' OR 'adolescent' OR 'adolescent'/exp OR adolescent OR 'young adult'/exp OR 'young adult' OR 'student'/exp OR 'student' OR 'depression'/exp OR 'depression' OR 'children'/exp OR children OR 'affective disorder'/exp OR 'affective disorder' OR 'affective disorders'/exp OR 'affective disorders' OR 'affective disturbance'/exp OR 'affective disturbance' OR 'affective illness'/exp OR 'affective illness' OR 'mood disorder'/exp OR 'mood disorder' OR 'mood disorders'/exp OR 'mood disorders' OR 'mood disturbance'/exp OR 'mood disturbance' OR 'mood disturbances'/exp OR 'mood disturbances') AND ('randomized controlled trial'/exp OR 'randomized controlled trial' OR 'sham procedure'/exp OR 'sham procedure' OR 'placebo'/exp OR 'placebo' OR 'placebo gel'/exp OR 'placebo gel' OR 'placebos'/exp OR 'placebos' OR 'open clinical study'/exp OR 'open clinical study' OR 'open clinical trial'/exp OR 'open clinical trial' OR 'open study'/exp OR 'open study' OR 'open trial'/exp OR 'open trial' OR 'open-label clinical study'/exp OR 'open-label clinical study' OR 'open-label clinical trial'/exp OR 'open-label clinical trial' OR 'open-label study'/exp OR 'open-label study' OR 'open-label trial'/exp OR 'open-label trial' OR 'open-labeled clinical study'/exp OR 'open-labeled clinical study' OR 'open-labeled clinical trial'/exp OR 'open-labeled clinical trial' OR 'open-labelled clinical study'/exp OR 'open-labelled clinical study' OR 'open-labelled clinical trial'/exp OR 'open-labelled clinical trial') AND ('hamilton depression rating scale'/exp OR 'hamilton depression rating scale' OR 'hamilton rating scale for depression'/exp OR 'hamilton rating scale for depression' OR 'hamilton scale'/exp OR 'hamilton scale' OR 'hamilton depression scale'/exp OR 'hamilton depression scale' OR 'hamilton rating scale'/exp OR 'hamilton rating scale' OR 'beck depression inventory'/exp OR 'beck depression inventory' OR 'beck depression inventory ii'/exp OR 'beck depression inventory ii' OR 'beck depression inventory revised edition'/exp OR 'beck depression inventory revised edition' OR 'beck depression scale'/exp OR 'beck depression scale' OR 'madrs'/exp OR 'madrs' OR 'montgomery asberg depression rating'/exp OR 'montgomery asberg depression rating' OR 'montgomery asberg depression rating scale'/exp OR 'montgomery asberg depression rating scale' OR 'montgomery asberg depression scale'/exp OR 'montgomery asberg depression scale' OR 'montgomery and asberg depression rating scale'/exp OR 'montgomery and asberg depression rating scale' OR 'center for epidemiologic studies depression scale'/exp OR 'center for epidemiologic studies depression scale' OR 'had scale'/exp OR 'had scale' OR 'had score'/exp OR 'had score' OR 'hads'/exp OR 'hads' OR 'hads (hospital anxiety and depression scale)'/exp OR 'hads (hospital anxiety and depression scale)' OR 'hospital anxiety depression scale'/exp OR 'hospital anxiety depression scale' OR 'hospital anxiety depression scale (hads)'/exp OR 'hospital anxiety depression scale (hads)' OR 'hospital anxiety and depression scale'/exp OR 'hospital anxiety and depression scale' OR 'hospital anxiety and depression scale (hads)'/exp OR 'hospital anxiety and depression scale (hads)' OR 'hospital anxiety and depression scale (hads-a, hads-d)'/exp OR 'hospital anxiety and depression scale (hads-a, hads-d)' OR 'hospital anxiety and depression scale (anxiety-subscale: hads-a and depression-subscale: hads-d)'/exp OR 'hospital anxiety and depression scale (anxiety-subscale: hads-a and depression-subscale: hads-d)' OR 'hospital anxiety and depression scale anxiety subscale (hads-a) and depression subscale (hads-d)'/exp OR 'hospital anxiety and depression scale anxiety subscale (hads-a) and depression subscale (hads-d)' OR 'hospital anxiety and depression scale sigmond and snaith'/exp OR 'hospital anxiety and depression scale sigmond and snaith' OR 'hospital anxiety and depression scale test'/exp OR 'hospital anxiety and depression scale test' OR 'hospital anxiety and depression scale-depression and anxiety'/exp OR 'hospital anxiety and depression scale-depression and anxiety' OR 'zigmond and snaith hospital anxiety and depression scale (hads)'/exp OR 'zigmond and snaith hospital anxiety and depression scale (hads)' OR 'zigmond and snaith`s hospital anxiety and depression scale (hads)'/exp OR 'zigmond and snaith`s hospital anxiety and depression scale (hads)' OR 'hospital anxiety and depression scale (hads-a/d)'/exp OR 'hospital anxiety and depression scale (hads-a/d)' OR 'mood states questionnaire') AND ('dim light'/exp OR 'dim light' OR 'light'/exp OR 'light' OR 'light diffusion'/exp OR 'light diffusion' OR 'light flash'/exp OR 'light flash' OR 'light induction'/exp OR 'light induction' OR 'light radiation'/exp OR 'light radiation' OR 'light source'/exp OR 'light source' OR 'light wave'/exp OR 'light wave' OR 'photoinduction'/exp OR 'photoinduction' OR 'photon radiation'/exp OR 'photon radiation' OR 'photoradiation'/exp OR 'photoradiation' OR 'visible light'/exp OR 'visible light' OR 'light treatment' OR 'chronotherapy'/exp OR chronotherapy OR 'wake and light therapy' OR 'light therapy'/exp OR 'light therapy' OR 'phototherapy'/exp OR 'phototherapy' OR 'light box' OR 'light beam'/exp OR 'light beam' OR 'light therapy device' OR 'light therapy lamp' OR luminere OR 'glasses'/exp OR glasses OR 'light room') AND ([randomized controlled trial]/lim OR 'controlled clinical trial'/de) AND ([child]/lim OR [preschool]/lim OR [school]/lim OR [adolescent]/lim OR [adult]/lim OR [young adult]/lim OR [middle aged]/lim) AND [humans]/lim AND [abstracts]/lim AND [clinical study]/lim |  |
| Cochrane library |  | 52 |
|  | ID Search Hits  #1 MeSH descriptor: [Depression] explode all trees 14311  #2 ("seasonal affective disorder"):ti,ab,kw 363  #3 ("seasonal mood disorder"):ti,ab,kw 0  #4 ("mood disorder"):ti,ab,kw (Word variations have been searched) 3639  #5 #1 or #4 or #2 or #3 17863  #6 MeSH descriptor: [Adolescent] explode all trees 110769  #7 (youth):ti,ab,kw 8443  #8 (teenager):ti,ab,kw 112  #9 ("adolescent"):ti,ab,kw (Word variations have been searched) 150272  #10 ("young adult"):ti,ab,kw (Word variations have been searched) 94805  #11 #6 or #9 or #10 or #8 or #7 206964  #12 MeSH descriptor: [Phototherapy] explode all trees 3648  #13 ("bright light therapy"):ti,ab,kw 343  #14 ("light therapy"):ti,ab,kw 2431  #15 (light treatment):ti,ab,kw (Word variations have been searched) 13142  #16 (Phototherapy):ti,ab,kw (Word variations have been searched) 3539  #17 (Chronotherapy):ti,ab,kw (Word variations have been searched) 465  #18 (wake and light therapy):ti,ab,kw (Word variations have been searched) 407  #19 #12 or #15 #16 or #17 or #18 or #14 or #13 5457  #20 ("Hamilton Depression Rating Scale"):ti,ab,kw (Word variations have been searched) 4487  #21 ("Beck Depression Inventory 2"):ti,ab,kw (Word variations have been searched) 57  #22 ("Beck Depression Inventory-II"):ti,ab,kw (Word variations have been searched) 998  #23 ("Beck Depression Inventory 2"):ti,ab,kw (Word variations have been searched) 57  #24 ("Montgomery Asberg Depression Rating Scale"):ti,ab,kw (Word variations have been searched) 2628  #25 ("Montgomery-Asberg Depression Rating Scale"):ti,ab,kw (Word variations have been searched) 2628  #26 ("Center for Epidemiologic Studies Depression Scale"):ti,ab,kw (Word variations have been searched) 1318  #27 ("light-box"):ti,ab,kw (Word variations have been searched) 104  #28 ("light boxes"):ti,ab,kw (Word variations have been searched) 104  #29 ("light box"):ti,ab,kw (Word variations have been searched) 104  #30 ("LAMP"):ti,ab,kw (Word variations have been searched) 2408  #31 #20 or #21 or #22 or #23 or #24 or #25 or #26 8953  #32 #27 or #28 or #29 or #30 or #31 11447  #33 #32 and #31 and #19 and #5 and #11 11  #34 #19 and #5 and #11 52 |  |
| Web of Science |  | 56 |
|  | TS=(juvenile OR juvenile OR youth OR adolescent OR adolescent OR teenager OR young adult OR adult, young OR prime adult OR prime adults OR young adult OR young adults OR student OR apprentice OR honor student OR honour student OR student OR students OR trainee) AND TS= (light OR dim light OR light OR light beam OR light diffusion OR light flash OR light induction OR light radiation OR light source OR light wave OR photoinduction OR photon radiation OR photoradiation OR visible light OR phototherapy OR light therapy OR phototherapy OR light treatment OR chronotherapy OR chrono therapy OR chronotherapy OR therapy, chrono OR wake and light therapy) AND TS=(hamilton depression rating scale OR hamilton depression rating scale OR hamilton rating scale for depression OR hamilton scale OR hamilton rating scale OR beck depression inventory OR beck depression inventory OR beck depression inventory ii OR beck depression inventory revised edition OR beck depression scale OR seasonal affective version OR montgomery asberg depression rating scale OR madrs OR montgomery asberg depression rating OR montgomery asberg depression rating scale OR montgomery asberg depression scale OR montgomery and asberg depression rating scale OR center for epidemiologic studies depression scale OR hamilton depression scale) AND TS= (randomized controlled trial OR controlled trial, randomized OR randomised controlled study OR randomised controlled trial OR randomized controlled study OR randomized controlled trial OR trial, randomized controlled OR open study OR open clinical study OR open clinical trial OR open study OR open trial OR open-label clinical study OR open-label clinical trial OR open-label study OR open-label trial OR open-labeled clinical study OR open-labeled clinical trial OR open-labelled clinical study OR open-labelled clinical trial OR pilot study OR pilot projects OR pilot studies OR pilot study OR study, pilot) |  |
| APA PsycInfo |  | 127 |
|  | (light therapy or light treatment or phototherapy or chronotherapy or dim light or DRL or BWL or bright light therapy or dim light therapy or BLT or light boxes or light glasses) AND (depression or depressive disorder or depressive symptoms or major depressive disorder or mood disorder or depressed or SAD or NAD or mania or PTSD or fatigue or affective disorder) AND (youth or adolescent or young people or teen or young adults or teenagers or teens or juvenile or student) |  |
| CINAHL |  | 90 |
|  | (light therapy or light treatment or phototherapy or chronotherapy or dim light or DRL or BWL or bright light therapy or dim light therapy or BLT or light boxes or light glasses) AND (depression or depressive disorder or depressive symptoms or major depressive disorder or mood disorder or depressed or SAD or NAD or mania or PTSD or fatigue or affective disorder) AND (youth or adolescent or young people or teen or young adults or teenagers or teens or juvenile or student) |  |
| Medline (OVID) |  | 125 |
|  | 1 (light therapy or light treatment or phototherapy or chronotherapy or dim light or DRL or BWL or bright light therapy or dim light therapy or BLT or light boxes or light glasses).mp. [mp=ti, ab, hw, tn, ot, dm, mf, dv, kf, fx, dq, tx, sh, sw, ct, bt, nm, ox, px, rx, an, ui, sy, ux, mx, tc, id, tm] 126170  2 (depression or depressive disorder or depressive symptoms or major depressive disorder or mood disorder or depressed or SAD or NAD or mania or PTSD or fatigue or affective disorder).mp. [mp=ti, ab, hw, tn, ot, dm, mf, dv, kf, fx, dq, tx, sh, sw, ct, bt, nm, ox, px, rx, an, ui, sy, ux, mx, tc, id, tm] 3750555  3 limit 2 to ("all child (0 to 18 years)" or "adolescent (13 to 18 years)" or "young adult (19 to 24 years)" or "adult (19 to 44 years)" or "young adult and adult (19-24 and 19-44)" or 100 childhood <birth to age 12 yrs> or 180 school age <age 6 to 12 yrs> or 200 adolescence <age 13 to 17 yrs> or 320 young adulthood <age 18 to 29 yrs>) 2906787  4 limit 3 to full text 410185  5 limit 4 to human 384438  6 limit 5 to (child <unspecified age> or school child <7 to 12 years> or adolescent <13 to 17 years>) 230307  7 limit 6 to humans 230307  8 1 and 2 and 7 931  9 (MADRs or HAMD-A or HAMD-D or HAMD or SIGH-SAD-SR or CES-D or DASS or BDI or HDRS or CGI-I or HAMA or QIDS or POMS or hamilton depression or beck depression inventory or scales Depression Anxiety Stress Scale or QIDS).mp. [mp=ti, ab, hw, tn, ot, dm, mf, dv, kf, fx, dq, tx, sh, sw, ct, bt, nm, ox, px, rx, an, ui, sy, ux, mx, tc, id, tm] 284520  10 8 and 9 125 |  |
| PubMed |  | 37 |
|  | ((((((((light treatment[Title/Abstract]) OR ("Phototherapy"[Mesh])) ) OR (bright light therapy[Title/Abstract])) OR (blue-enriched light therapy[Title/Abstract])) OR (Chronotherapy[Title/Abstract])) OR (wake[Title/Abstract] AND light therapy[Title/Abstract])) AND (((("Adolescent"[Mesh]) OR (youth)) OR (young adults[Title/Abstract])) OR (student[Title/Abstract]))) AND (("Depression"[Mesh]) OR (mood disorder[Title/Abstract])) |  |
| Scholar |  | 100 |
|  | (light therapy or light treatment or phototherapy or chronotherapy or dim light or DRL or BWL or bright light therapy or dim light therapy or BLT or light boxes or light glasses) AND (depression or depressive disorder or depressive symptoms or major depressive disorder or mood disorder or depressed or SAD or NAD or mania or PTSD or fatigue or affective disorder) AND (youth or adolescent or young people or teen or young adults or teenagers or teens or juvenile or student) |  |
| Total after de-duplic ation |  | 599 |

# Table S2. Detailed characteristics of the included trials (n=31)

| **Study/ Country/** | **Study Design** | **Duration** | **Accumulative exposure time (min)** | **Detailed parameters for CS calculation** | **Diagnosis** | **CS_t,f_** | **CL_A_** | **Other treatment** | **Side effect/** **Drop out** | **Mean age (years±SD)** | **Sample size (n)** | **Sex: male/female (n/n)** | **Mood measure** | **Other Outcome** | **Results** |
| --- | --- | --- | --- | --- | --- | --- | --- | --- | --- | --- | --- | --- | --- | --- | --- |
| **Random-controlled trials**  **Diagnosed with depression and Medicated** | | | | | | | | | | | | | | | |
| Gest, S,2015, Germany [87] | LT compared with wake therapy (1 night WT) +LT, among inpatients | 2weeks，8：00am -10:00 am, 45 min of morning BLT, 5 times a week | 2 week-450 | LT-box-white light of 10000lux (LD 110; DAVITA Medizinische Produkte GmbH & Co.), 28W/m^2^ | moderate to severe depressive symptoms (BDI-II), 6 in each group with SAD pattern | Tx: CS_t0.75_=0.665 | Tx: 6803.7 | 41 (66.5 %) were without antidepressants, 11 (18 %) patients were medicated before LT, 6 (10 %) were additionally medicated after 2 weeks, 4 (5.5 %) were newly medicated after follow up | Tx: 7 (18.9%), Control: 2 (8%) | Tx: 15.75±1.017;  Control: 16.16±1.275 | Tx: n=37; Control: n=25 | Tx: 2/35; Control: 3/22 | BDI-II | SDQ, CGI-I, German sleep questionnaire | No group differences at post-treatment in depressive symptom reduction dependent on treatment |
| Shawn D. Youngstedt el al, 2011, USA [[90](#_bookmark46)] | RCT among 18-35 yrs old college students | 4weeks, daily morning 45min | 1 week-315  2 week-630  3 week-945  4 week-1260 | Tx: 3000lx white light at the eye, peak at 460nm (Litebook Company, Alberta, Canada)  Control: inactivated negative ion generator (INIG) | Initial screened by [STAI] Form Y2), further received a SCID for DSM-IV, among whom MDD, OCD, PTSD, SP, ADA, GAD patients balanced between the groups | Tx: CS_t0.75_=0.652 | Tx: 5106.3 | Not reported, possibly with meditation | No significant side effects among those remained, e.g., headaches, eye problems | Tx: 22.0±1.0  Control: 21.4±0.6 | Tx: n=17;  Control: n=16 | Tx: 5/12; Control: 3/13 | BDI | HAM-D_21_, HAM-A, CGI-I | Reduction in STAI-psychic subscale significantly correlated with the reduction in BDI, no significant effects were found for the HAM-A, HAM-D, or CGI |
| Goel el al, 2006, USA [81] | a placebo-controlled study among 18-28 years old student population | 3 days，19:00-21:00pm, daily 30mins | 3 days-90 | SPX-30 tri-phosphor fluorescent lamps, 3000K, head-provided～10000 lux. The center of the screen was placed～32 cm from each subject’s eyes | 11 depressed subjects in spring, 13 in summer, 11 in autumn, total 29.7% were depressed | Tx: CS_t0.5_=0.635* | Tx: 5627.2 | Not reported, but those depressed may took medication | — | Overall: 19.4±1.7 | BLT: n=29;  Sound stimulus: n=30; High-density air flow rate: n=29; low-density air flow rate: n=30 | Overall: 49/69 | POMS | / | All three stimuli rapidly improve mood in a college sample among both depressed and non-depressed subjects |
| Richardson, 2021, Australia [76] | 13-24 yrs DSWPD adolescents compared with DRL and healthy good sleepers | 3 weeks, 3 times per week, wore portable bright light glasses for 30-60-mins promptly after rising from bed (50mins) | 1 week-150  2 week-300  3 week-450 | Tx: Re-Timer Light Therapy Glasses, 112lux (～51.0 μW/cm²),  Peak 507 nm, 2.0494 x 10^15^ photons/cm²/s  Control:  Re-Timer Light Therapy Glasses, 112lux, peak 643 nm | 30 self-reported diagnosed depression, mean depressive symptom score for those without diagnosed depression approached the clinical cut-off indicative of major depression | Tx: CS_t0.78_=0.242*  Control:  CS_t0.8_＜0.1 | Tx: 255.3  — | Not reported, those diagnosed depression may took medication | — | Tx: 16.07±2.4; Control: 15.6±2.2 | Tx: n=30; Control: n=30 | Tx: 11/19  Control: 11/19 | Short Mood and Feelings Questionnaire (SMFQ) | / | Depressive symptoms significantly improved in both groups (small improvements post-treatment & 1-month follow-up, large improvements at 3-month follow-up) |
| Flory, 2011, USA [97]**^a^** | a parallel-group design in a controlled laboratory setting among 18- | 12 days, specific 30-min treatment session, 7:30-11:00am | 12 days-360 | Tx: 2 light boxes (Bio-Light Ultra, Enviro-Med, Vancouver, WA), 3×40 W, 4100 K twin tri-phosphor fluorescent tubes, 10000lux at ～37 cm from diffusing screen center to the subject's eyes;  Control: box contained a single twin tube with a translucent red plastic panel, 300 lux at ~61 cm from the center of the screen to eyes | ≥20 points on the overall SIGH-SAD-SR, required to meet each of the 4 DSM-IV criteria for SAD by answering “yes” to the 4 questions provided in a symptom checklist derived from the Criteria for Seasonal Pattern Specifier | Tx: CS_t0.5_=0.642-0.663  Control-DRL: CS_t0.5_=0.129 | Tx: 6359 -9821.7  Control-DRL: 330.94 | All psychotropic medicated | — | Overall: 20.8±5.69 | BLT: n=19; DRL: n=16; high-density negative ions (HDNI): n= 18; low-density negative ions (LDNI): n= 20 | All females | SIGH-SAD-SR, HAM-D, BDI-II | / | When remission outcome criteria were used, BWL was significantly more effective than any of the other three treatments |
| LaRosa, 2022, USA [[68](#_bookmark33)] | among 12-22 years old newly diagnosed solid tumors and received chemotherapy | 8 weeks, 30 min within 1 h of waking | 8week-1680 | Tx: Litebook Advantage, The Litebook Company Ltd., Medicine Hat, Alberta, Canada, emitting 10000lx, one arm distance at eye level (3000-10000lx);  Control: red Light peak 680nm (The Litebook) ～50lux | Cancer-related fatigue | Tx: CS_t0.5_=0.64 (0.549-0.679)  Control: CS_t0.5_＜0.1* | Tx: 5106.3 (2301.8-16949.2) | Recently initiated antidepressant medication was excluded | — | Overall: 15.96±2.41 | Tx: n=21^c^;  Control: n=23 | Tx: 8/13  Control: 11/12 | CDI-II | / | BWL reported a greater rate of improvement than DRL for total depression |
| Spezzano, 2006, USA [88] | randomized, double-blind, parallel design among aged 18-22 college-aged students | 3 weeks, 30 minutes a day (in the morning), | 1 week-210  2 week-420  3 week-630 | Tx: SunBox JR (SunBox Company) 10,000 lux at a distance of 14 inches (35cm);  Control: inactivated ion generator as placebo | Diagnosed with Seasonal Affective Disorder (SAD diagnostic criteria) | Tx: CS_t0.5_=0.658* | Tx: 8711.1 | No recently used antidepressant/ herbal products that act much (within 8 weeks of study), or who changed doses in same time frame | — | Tx: 19.75±1.4; Control:19.7±1.2 | Tx: n=20; Control: n=20 | Tx: 9/11  Control: 7/13 | SIGH-SAD, BDI-II | / | Dramatic improvement in BLT group with a remission criterion of a 50% reduction in symptoms, 80% of BLT group but none in control were in remission at the end |
| Blouin, 1996, Canada [71] | double-blind, placebo-controlled study among ＞17 yrs old people | 1 week, daily 2 hours in the early evening, 17:00-19:00pm | 1 week-840 | light box (Duratest) with 2500 lux of full-spectrum fluorescent light,  Control: 500lux of full-spectrum fluorescent light as placebo | bulimia nervosa (DSM-III-R), 13 were MDD (NIMH-DIS-R), 3 were seasonal mood pattern (DSM-III-R), 10 were SAD (SPAQ) | Tx-2500lux: CS_t2.0_=0.669  Control-500lux: CS_t2.0_=0.552 | Tx-2500lx: 2933.5  Control-500lux: 588.5 | 3 subjects under antidepressant medication, 1 subject under a mild dose of anxiolytic medication. No beginning or ending of medication during participation | — | Overall: 27.9±8.0 | Tx: n=9;  Control: n=9 | Tx: 0/9;  Control: 0/9 | SIGH-SAD, BDI, | Bulimic Symptoms Checklist, POMS, Daily Binge Record (DBR) | Subjects in the BLT condition showed a significant improvement in depressed mood during light exposure than those in the DLT group (BDI, SIGH-SAD) |
| Braun el al, 1999, USA [[70](#_bookmark35)] | double-blind, placebo-controlled study among 18-50 years old female outpatients | 3 weeks, daily 90mins at home 6-9am | 3 week-1890 | Tx: Apollo light boxes (Orem, UT), 10000lux at the retina, full spectrum cool white fluorescent  Control: 50lx red dim light at retina | SCID-determined bulimia nervosa, 4 of 16 subjects (25%) from active group and 4 of 18 (22.2%) from placebo group met criteria for MDD, none met criteria for MDD with a seasonal pattern (SCID) | Tx: CS_t1.5_=0.690  Control: CS_t1.5_＜0.01 | Tx: 11654.8 | with ongoing psychotherapy and medications, those dose changes excluded | No subjects withdrew from the study due to side effects. | Tx: 30.50±7.3;  Control: 30.50±8.6 | Tx: n=15;  Control: n=16 | Tx:0/15; Control: 0/16 | SIGH-SAD, BDI, | YBC-EDS, SPAQ | Scores significantly decreased across time, but there were no differences between groups |
| Bais, 2020, the Netherlands [[73](#_bookmark39)] **^b^** | a randomized, double-blind controlled trial among postpartum female | 6weeks, daily 30mins within 30min of habitual wake up time | 1 week-210  2 week-420  3 week-630  4 week-840  5 week-1050  6 week-1260 | Tx: EnergyUp HF3419/01, Philips, Eindhoven, Netherlands,9000lux, 5000K (LED), with a distance of 40cm  Control: 2700 K, 100lux LED DRL | DSM-5 diagnosis of depressive disorder | Tx: CS_t0.5_=0.657  Control:  CSt_0.5_＜0.1 | Tx: 8395.5 | 4 women started psychotropic medication, 11 women started psychotherapy (3 during intervention, 1 after intervention during pregnancy and 7 in postpartum period) | Tx: headaches (30.3%), sleep problems (12.1%), nausea (6.1%); Control: headaches (20.6%), sleep problems (8.9%), irritable eyes (5.9%), 2 drop outs | Tx: 31.9±4.4; Control: 31.9±5.3 | Tx: n=33; Control: n=34 | Tx: 0/33;  Control: 0/34 | SIGH-SAD, HAMD-17, EPDS | / | No statistically significant difference was found between the two treatment arms for the intervention period, nor for the entire study. |
| **Randomized controlled trials**  **Depressed but non-medicated** | | | | | | | | | | | | | | | |
| Bogen el al, 2015, Germany [[8](#_bookmark48)7] | ITT design among 12-18 years old inpatients | 2weeks，45 min of morning BLT, 5 times a week | 2 week-450 | Tx: 10,000 lx by PhysioLight LD1100 (DAVITA) ～65 cm away;  Control: DAVITA Luxor LED (max.2500 lx), 100-150lux (60% power) at～65 cm | moderate to severe depression (ICD-10), ≥20 points (BDI-II) | Tx: CS_t0.75_=0.665*  Control: CS_t0.75_＜0.1 | Tx: 6803.7 | Combined treatment, but unmedicated | Headaches, Tx: 9 (30%); Control: 11 (40.7%) | Tx: 15.4±1.6;  Control: 15.3±1.5 | Tx: n=30;  Control: n=27 | Tx: 11/19  Control: 4/23 | BDI-II | SDQ, German sleep questionnaire, CGI-I, SPAQ |  |
| Jiang, 2020 China [31] | Three arm RCT among 18-25 years old college students | 8 weeks, daily 30mins (before 12 pm), most in 11:00 - 12:00 am | 4 week-840  8 week-1680 | custom-made light boxes (LED) in Jinan University, 5000lx white cold light, 5000K (analogy with CIE illuminant D50), the device was on a desk at a distance of 50 cm | (8 ≤ HAMD-24 ≤ 20. Nonseasonal subthreshold depression if with depressed mood or anhedonia, plus 1 to 3 depressive symptoms for at least 2 weeks that caused functional impairment | Tx-5000lx: CS_t0.5_=0.639 (0.622-0.648)  Tx-500lx: CS_t0.5_=0.277(0.272-0.30) | Tx:5000lx  4686.6- 7060.6  Tx:500lx  472.6-549.7 | No drugs or other intervention (any form of interventions for depression within the past year were excluded) | — | Tx-5000lx: 21.18±2.31;  Tx-500lx: 21.49±2.35;  Control: 21.38±2.22 | Tx-5000lx: n = 51;  Tx-500lx: n=51;  Control: n=42 | Tx-5000lx: 16/35; Tx-500lx:13/38; Control: 15/27 | BDI-II, HAMD-24, SAI | / | Both low- and high-intensity light therapy are effective in reducing depressive and anxiety symptoms in young adults |
| Janas-Kozik el al，2011, Polland [[29](#_bookmark30)] | BLT compared with CBT among 15–20 years old girls | 6 weeks, 30 min daily morning | 1 week-210  2 week-420  3 week-630  4 week-840  5 week-1050  6 week-1260 | 10 000 lux lamp, no other information | Anorexia nervosa (AN-R) (DSM-IV), ≥17 points (21-item HDRS) | Tx: CS_t0.5_=0.60-0.69 | — | Combined CBT/ psychotherapy, no drugs | — | Tx: 17.8±1.34; Control: 17.0±1.34 | Tx: n=12;  Control: n=12 | Tx:0/12;  Control: 0/12 | HDRS | SDQ, BMI | A reduction of depressive symptoms after 6 weeks was significantly greater than in those without such treatment |
| Donmez, 2022, Turkey [30] | randomized-controlled, double-blind study | 3 weeks, 45 min adhering to a fixed wake-up time (preferably before 10 a.m.) | 1 week-315  2 week-630  3 week-945 | Tx: 10000lux, Beurer TL100 daylight therapy LED lamp; Beurer, Ulm, Germany), 5000K, 40–45 cm from the box (～2500lux at eye)  Control: <500lux dim light (～125lux at eye) | MDD (DSM-5 criteria), ≥12 points on the EPDS, no current diagnosis of any psychiatric or major medical comorbidity | Tx: CS_t0.75_=0.59;  Control: CS_t0.75_≈0.12 | Tx: 2353.1  Control:  118.3 | None received any form of intervention (medication, psychotherapy, etc.) within the past 6 months or any intention for co-intervention during the study | Tx: 6 mild headache; Control: 1 mild insomnia, 1 mild headache | Tx: 29.73±6.57; Control: 28.0±3.8 | Tx: n=15;  Control: n=15 | Tx:0/15; Control: 0/15 | [MADRS](https://www.sciencedirect.com/topics/medicine-and-dentistry/montgomery-asberg-depression-rating-scale), HAM-D, EPDS | PSQI | BLT applied in the morning for 3 weeks was more effective than the placebo in terms of treatment response and remission rates, and side effects were similar to placebo |
| Epperson, 2003, USA [[22](#_bookmark42)] | double-blind placebo-controlled pilot study among 24–37 years old antepartum depressed woman | 5 weeks, daily 60mins, | 5 week-2100 | Tx: broad-band fluorescent light, 7000lux diffusing at 33cm, modified HealthLight, SphereOne Inc., Silver Plume, Colo.  Control: 500lux at 33cm | MDD (DSM-IV), no co-morbid Axis I disorder or sleep disorder, only 1 subject enrolled met seasonal pattern criteria (DSM-IV) | Tx: CSt1.0=0.678-0.682*  Control: CSt1.0=0.497* | Tx: 8175.3-9888  Control: 514.9-799.5 | Current  use of psychotropic medication excluded | transient hypomania appeared in a woman with no previous history | Overall: 32.10±3.9 | Tx: n=4; Control: n=5 | Tx: 0/4  Control: 0/5 | SIGH-SAD | / | A small mean group advantage of active treatment existed throughout the RCT, but not statistically significant. |
| Grandner, 2007, USA [[82](#_bookmark53)] | 18-35 years old students | 12 days, 150mins prior to usual wake time (30 mins intensity 0%-100%) | Tx: 10.7 days-1605;  Control: 11.3 days-1695 | Tx: bright green light mask, (～ 10,000 lux with light from green LEDs set at 100% intensity), peak～500 nm;  Control: mask of dim red light (0.5 lux, white LEDs at 1% intensity filtered through red gel) | Some were healthy young people/ some were with minimal to mild depression | Tx: CS_t2.5_=0.692-0.694  Control: CS_t2.5_<0.1 | Tx: 7958.9-  40533.4 | All drug-free | Some slight “eye” or “chest” symptoms | Tx: 23.13; Control: 22.13 | Tx: n=15  Control: n=15 | Tx: 15/0  Control: 15/0 | QIDS-SR | ESS, MVAS, SAFTEE | No differences in mood rating changes between treatments |
| **Quasi-experimental trials**  **Depressed and medicated** | | | | | | | | | | | | | | | |
| Swanson, 2017, USA, [[74](#_bookmark40)] | Open-label pilot study, one-group pretest posttest among ＞18 yrs postpartum female | 5 weeks, daily 60 mins within 30 mins of waking (at least 30mins) | 5 week-1050-2100 | Re-Timer Light Therapy Glasses, 506lux, Green-blue 500 nm | MDD (DSM-V), scored ≥ 20 on the SIGH-SAD | Tx: CS_t0.5_=0.451*  CS_t1.0_=0.557* | Tx: 1222.5 | light-sensitizing medication excluded, those with a stable medication were included. 3 (30%) were antidepressant medicated (fluoxetine or bupropion) | after 3 weeks 1 dropped out due to side effects (irritability and headache) | Tx: 32.30±3.27 | Tx: n=10 | 0/10 | SIGH-SAD | / |  |
| Kirschbaum-Lesch el al, 2018, Germany [64] | one-group pretest posttest (single arm) among 12-18 yrs youth | 4weeks，morning BLT for 30 minutes, weekends excluded | 2 week-600  4 week-1200 | LT glasses- Luminette® (Lucimed, Belgium) blue light (468 nm) with a power of 10,000 lux | moderate to severe depressive symptoms (BDI-II) | Tx: CS_t0.5_=0.686 | 24069.5 | antidepressant medication was allowed, and controlled for in the analyses performed | 1/3 participants drop out, reported short-lasting headache and dizziness | Tx: 15.74±1.14 | Tx: n=39 | 7/32 | BDI-II | German sleep questionnaire, CGI | significant improvements in sleep quality and depressive symptoms |
| Lisa A. House el al, 2018, USA [[6](#_bookmark26)2] | one-group pretest posttest | 4 weeks，15mins for 1st week, 30-minutes 8:00am-11:00 am, Monday to Friday for next 3 weeks | 4 week-525 | light box 10,000 lux-NorthStar™ light box, Alaska Northern Lights, 4100K, 2 broad spectrum Biax bulbs (10000lux at 24 inches) | brief medical and psychological screenings of signs and symptoms of depression or SAD | CS_t0.5_=0.667-0.668 | 10778-11218 | 44% people were receiving other treatments, those with a medical condition that makes the skin sensitive to light were excluded | 6 who did 15-minute sessions for the first 2 weeks due to headaches experienced during the first week | Tx：19-21 | Tx: n = 79 | 18/61 | BDI-II | / | Significant improvements in depression scores, improved sleeping behaviors and decreases in somatic aches, pains problems |
| Papatheodorou, 1995, Canada [8[3](#_bookmark45)] | one-group pretest posttest | 7 days，7:00am-9:00am+19:00-21:00pm，daily 45 min to 60 min | 1 week-650 | cool-white fluorescent light (Medic Light^TM^), 10,000 lux (analogy with CIE F2/F7 illuminants) | bipolar disorder (DSM-III-R) | Tx: CS_t0.75_=0.679 (0.663-0.683);  CS_t1.0_=0.685 (0.682-0.692) | 9821.71-20245.7 | all were medicated | None of the patients in this study reported any adverse events during BLT treatment | Tx:19.4±2 | Tx: n=7 | 2/5 | BDI-II | Symptoms Check List (SCL) | 3 patients showed a response of ＞70% decrease of baseline score. 2 patients had a moderate decrease (40%-74%) and 2 patients obtained mild to no response. |
| Nixon, 2021, Canada [[92](#_bookmark56)] | open-label study among 15-30 years old outpatients | 4 weeks, 45 to 60 min upon daily awakening | 2 week-735 (630-840)  4 week- averagely 21 days (620-1240mins) | Re-Timer Light Therapy Glasses, 112lux,  Green-blue 507 nm | depressive syndrome (e.g., dysthymia) or at least mild depressive symptoms on QIDS-SR-16 or QIDS-A17 | Tx: CS_t0.78_=0.242*  CS_t1.0_=0.287 * | Tx: 255.3 | Over half took psychotropic medication or antidepressants, No one started medications that affect sleep, circadian rhythms, alertness within the month preceding study | — | Tx: 21.2±1.0 | Tx: n=24^d^ | 4/20 | BDI-II | Leeds Sleep Evaluation Questionnaire | BDI-II scores decreased significantly after 4weeks of intervention |
| T. Kopp el al, 2016, USA [67] | one-group pretest posttest among hospitalized CF patients | 1week, daily 30min, 8:00-10:00am | 1 week-210 | 10,000 lux bright white light, Sun Touch Plus Light and Ion Therapy Lamp© (17000K, 4×8W fluorescent T5 Tubes) | 80% of Hospitalized CF patients had at least mild depressive symptoms upon admission (QIDS) | Tx: CS_t0.5_=0.669-0.684* | Tx: 11650-21772 | 57% were on at least one prescribed antidepressant, ＞85% with Vitamin-D supplements | no adverse events reported | Tx: 27.7±8.5 | Tx: n=30 | 17/13 | QIDS-C | quality of life factors (CFQ-R) | Significant decrease in depressive symptoms for CF patients all receiving LT. |
| Ricketts, 2022, USA [[69](#_bookmark34)] | TD vs  Healthy controls | 2 weeks, 60 mins daily upon awakening at their average rise time, (45 mins) | 2 week-840 | Re-Timer Light Therapy Glasses, 112lux,  peak 507 nm | 85.7% TD, 57.1% MDD, 14.3%, with persistent motor tic disorder | Tx: CS_t0.75_=0.235* | Tx: 255.3 | 4 (28.6%) were on tic medication, 5 (35.7%) were on other psychotropic medication | physical discomfort | Tx: 27.86±5.20;  Control: 31.75±8.49 | Tx: n=14; Control: n=20 | Tx:10/4 | DASS | / | A significant circadian phase advance but no significant improvements in depression or anxiety was shown |
| Bromundt, 2013, Switzerland [[7](#_bookmark37)2] | 23–41 years old BPD woman vs healthy females and no LT condition | 18 days, 30–40 min daily, within 9:00am | 3 week-630 | Daylight® classic box, Uplift Technologies, Canada, 8000 lux (broad-spectrum fluorescent white light) | Borderline personality disorder (DSM-IV), with a cutoff value > 8 in the Borderline Personality Inventory (BPI), 7 (50%) SAD, 2 (14%) subsyndromal form | Tx: CS_t0.5_=0.647-0.669* | Tx: 8546.4-11600 | All patients underwent psychotherapeutic treatment, 4 were unmedicated, 10 were on stable medication for at least 2 weeks prior to the study | — | Tx: 30.1±6.0;  Control: 25.7±4.8 | Tx: n=14; Control: n=10 | Tx: 0/14;  Control: 0/10 | SIGH-ADS-SR, BDI-II | BSL-95, STAI, | Depression scores in BPD did not improve with BLT, the atypical depression scores (SIGH-ADS-SR A8) significantly improved in BPD women during LT compared to oLT |
| **Non depressed and non-medicated** | | | | | | | | | | | | | | | |
| Lee, 2013, USA [[79](#_bookmark41)] | a randomized double-blind pretest–posttest design among 19-41 years old females | 3-week (average 18 days), 30-min daily, | 3 week-550 | Tx: bright light visor (Feel Bright Light model VZ 100, Physician Engineered Products, Fryeburg, ME), 8000lux of gentle blue–green light (470-525/peak～500 nm), fixed close to the eyes (3000-8000lux);  Control: dim red light | healthy first-time mothers with Low-Birth-Weight Infants without any preexisting depression | Tx: CS_t0.5_=0.663-0.688* | Tx: 9801.6-27783.6 | no medication | — | Tx: 24.4±5.4; Control:29.1±6.7 | Tx: n=16; Control: n=14 | Tx: 0/16; Control: 0/14 | EPDS | LFS, Medical Outcomes Short Form-36, version 2 (SF36v2) | Small to large effect sizes were found between pre- to posttreatment differences, no statistically significant difference of depressive symptoms between 2 groups |
| Sasseville, 2015, Canada [[80](#_bookmark44)] | RCT to investigate the contribution of short wavelengths in the alerting effect of bright light | 30 min at 3:00 am, | 1 day-30 | Tx: Litebook® Elite™ (Litebook®, Medicine Hat, AB, Canada), 1420lux at eye level (500μW/cm2, 2.78E+14 photons/cm2/s);  Control: Blue-blocking glasses (Chron-optic glasses, Québec, QC, Canada) 1150lux at eye level (500μW/cm2,2.12E+14 photons/cm2 /s) | All in good mental and  physical health | Tx: CS_t0.5_=0.556  Control: CS_t0.5_<0.1 | Tx: 2420.9  Control: 130.5 | No medications | — | Tx: 24.5±1.5 (21-26 years)  Control: 27.4±1.8 (25-30 years) | Tx: n=10;  Control: n=10 | Tx: 5/5  Control: 4/6 | VAS | SSS |  |
| Raikes, 2020, USA [[78](#_bookmark32)] | double-blind placebo-controlled randomized trial among Mild traumatic brain injuries | 6 weeks, daily 30 mins, 2 hours of waking, 8:00-10:00 am | 6 week-1260 | Tx: blue light Philips goLITE BLU (peak∼469-480nm), 214 lux at eye level, panel irradiance 1.23 mW/cm^2^)  Control: Philips Electronic, amber light Custom light box (ALT peak∼578 nm, 188 lux at eye level, 0.35 mW/cm^2)^ | Mild traumatic brain injuries (Veteran’s Administration/Department of Defense criteria), Axis I disorders were excluded (DSM-IV) | Tx: CS_t0.5_=0.580-0.585;  Control: CS_t0.5_＜0.1-0.135 | Tx: 2995.3-3116.9  Control:  63.5-194.3 | Presumed no psychotropic drugs | — | Tx: 25.53±8.65; Control: 26.63±7.62 | Tx: n=17; Control: n=18 | Tx: 5/12; Control: 8/10 | BDI-II | RPCSQ, ESS, Outcomes of Sleep Questionnaire (FOSQ) | After controlling for baseline values, moderate to large between-group differences with the BLT group was observed, reporting lower BDI-II |
| Huang,2013, China [[63](#_bookmark28)] | prospective, RCT study among nurses on rotating shifts during the evening or night shift | 10 days, over 30mins, evening shift exposure 19:30-20:30pm, night shift exposure 23:00-24:00pm | 10 days-300 | Tx: Apollo briteLITE 6, 5000-6000lx at ～70 cm away (32 inches for 3000lx and 22 inches for 6000lx);  Control: a sham light box of much lower intensity or red light, also wore dark sunglasses | moderate to severe insomnia (ISI score > 14, clinical insomnia) | Tx: CS_t0.5_=0.651 (0.638-0.656) | Tx: 5850.1-8175.3 | Majority were without psychotropic medication other than sleep medications (only a few) | — | Tx: 30.2±4.5; Control: 30.3 ±4.7 | Tx: n=46;  Control: n=46 | Tx: 0/46  Control: 0/46 | HADS, HADS-D, | HADS-A, ISI | The ISI, HADS, and subscales of the HADS scores were significantly improved across treatments in the treatment group compared with the control group. |
| Li, China, 2021 [[75](#_bookmark57)] | 16-60 years old DSWPD patients vs sleep-normal people also wore LT glasses | 1week, daily 60mins between 06:30-09:00 am | 1 week-420 | LT glasses (PEGASI intelligent sleep glasses, PG16K02), peak 470 nm | DSWPD- Delayed Sleep-Wake Phase Disorder, | Tx: CS_t1.0_=0.302-0.507* | Tx: 854.14529 (276.4-17501) | No medication | — | Tx: 29.73±8.98; Control: 34.9±10.80 | Tx: n=15; Control: n=15 | Tx: 4/11 | HAMD-24 | HAMA-14, PSQI, ISI, ESS | sleep-wake phase of the patients was shifted forward to a certain extent. |
| van Kol, 2015, the Netherlands [[77](#_bookmark55)] | a crossover design study, within subject counterbalanced design | 2 weeks, 9.00 am at the latest, on weekdays for 20-30 minutes daily, with 2 lamps each week | 2 week-300 | Tx: EnergyUp HF3419/01, Philips, Eindhoven, The Netherlands, 984-1088 lux at a distance of ～50cm, 4590K;  Control: micro living colors table light, Philips-7001831PH, 205lux of Dim Red Light (DRL) | Teachers who scored ＞2.0 and students scored ＞2.2 on the exhaustion scale of the UBOS, some were mildly depressed | Tx: CS_t0.5_=0.401-0.420*  Control: CS_t0.5_ ＜0.1 | Tx: 929.1-1027 | no reported medication | Some burden | Overall: 28.28±14.10 | Tx: n=29; | Tx:10/19 | BDI-II-NL (Dutch), | MBI, UBOS, FAS, BO-NKS, Shortened Fatigue Questionnaire, WLEQ (Weekly Light Experience Questionnaire) | BLT did not significantly differ in influencing depressive mood compared to DRL, both lamps had lowered depressive mood |
| Danielsson el al, 2014, USA [6[5](#_bookmark49)] | nonblinded RCT among 16-26 years old DSPD young adults | 2 weeks, daily morning BLT for 30-45min at home | 2 week-420 | Tx: 10000lux white-The Brite LITE 6 energy lamp, Philips (fluorescent)  Control: LT+CBT | DSPD (International Classification of Sleep Disorders -American Academy of Sleep Medicine, 2005) | Tx: CS_t0.5_=0.675  (0.656-0.673)  CSt_0.75_=0.684  (0.671-0.683) | Tx: 8297.8-13202.8 | During the 1^st^ 2-week LT, no psychiatric treatments. 1 in LT+CBT group used drugs. Before 6 month follow up, 1 in each group received psychiatric treatment, 1 in each arm received sleep medication | — | Tx: 22±3  Control: 22±2 | Tx: n=19;  Control: n=17 | Tx:9/10; Control:10/7 | HADS-D | HADS-A, ISI | no significant group differences were observed in the primary endpoints |
| ^a^11 remained on prescribed medications other than psychotropic drugs, 8 remained on a psychotropic medication regimen of either a SSRI (6 subjects) or a norepinephrine/dopamine reuptake inhibitor (2 subjects).  **^b^**11 started a SSRI, 1 woman in the postpartum period (both sertraline), 1 with an antipsychotic (quetiapine) and 1 with a benzodiazepine (temazepam) postpartum. The escitalopram dose increased in the postpartum period of 1 participant.  **^c^** Although 26 patients in BWL group and 25 patients in DRL group were randomized, data were available only for 21 in BWL group and 23 patients in DRL group  **^d^** 31 participants were recruited but only 24 completed at least 2 weeks of the intervention and were included in the analyses: Adherence data was missing for 1 participant. During the four weeks (28 days) of intervention, 12 participants (50%) reported using the light therapy glasses in the morning for 30 to 60 minutes between 22 and 29 days, 6 participants (25%) reported using them between 14 and 21 days, and 5 participants (21%) reported using on less than 14 days.  **Abbreviations:** MDD, Major Depressive Disorder; OCD, Obsessive–Compulsive Disorder; PTSD, Posttraumatic Stress Disorder; SP, Social Phobia; ADA, Alcohol/ Drug abuse; GAD, Generalized Anxiety Disorder; DSPD, delayed sleep phase disorder; DSWPD, Delayed Sleep-Wake Phase Disorder; CF, Cystic fibrosis;  SCID, Structured Clinical Interview for DSM-IV Axis I Disorders; CGI, Clinical Global Impression of Severity scale; STAI, State–Trait Anxiety Inventory (Form Y2); QIDS, The Quick Inventory of Depressive Symptomatology self-report; LOS, length of stay; TD, Tourette’s disorder; LT, light therapy; BWL, bright white light; CBT, cognitive– behavioral psychotherapy; EPDS, Edinburgh Postnatal Depression Scale; DSM, Diagnostic and Statistical Manual of Mental Disorders; HDRS, 21-item Hamilton Depression Rating Scale; HADS, Hospital Anxiety and Depression Scale; ISI, Insomnia Severity Index; HADS-A, anxiety subscale of the Hospital Anxiety and Depression Scale; HADS-D, depression subscale of the Hospital Anxiety and Depression Scale; SIGH-SAD, Structured Interview Guide for the Hamilton Depression Rating Scale-Seasonal Affective Disorder; SIGH-SAD-SR, Structured Interview Guide for the Hamilton Depression Rating Scale–Seasonal Affective Disorder Version–Self Rating; SDQ, The Strength and Difficulties Questionnaire (SDQ- emotional problems, hyperactivity, behavioral problems with peers and ability to socialize in 25 items); SAI, state anxiety inventory; HAMD-24, Hamilton Depression Rating Scale; HAM-A, Hamilton Anxiety Scale; ICD-10, International Statistical Classification of Diseases and Related Health Problems; SPAQ, Seasonal Pattern Assessment Questionnaire; BMI, body mass index; BDI-II, Beck Depression Inventory Second Edition; MAVS, mood visual analogue scale; ESS, Epworth Sleepiness Scale; DASS, Depression Anxiety Stress Scale; KSS, Karolinska Sleepiness Scale; POMS, The Profile of Mood States Questionnaire; PSQI, Pittsburgh Sleep Quality Index; MBI, the Maslach Burnout Inventory; UBOS, Utrechtse Burnout Schaal; FAS, Fatigue Assessment Scale; BO-NKS, Burnout- Neurasthenia Complaints Scale; RPCSQ, the Rivermead Post-concussion Symptoms Questionnaire-The Functional; YBC-EDS, Yale-Brown-Cornell Eating Disorder Scale; LFS, Lee’s Fatigue scale; CDI-II, Children’s Depression Inventory, 2nd Edition; BSL-95, Borderline Symptom List; VAS, visual analog scales; SSS, Stanford Sleepiness Scale; NIMH-DIS-R, National Institute of Mental Health Diagnostic Interview Schedule-Revised;  SSRI, selective serotonin reuptake inhibitor; Tx = Treatment condition; LED = light emitting diode; | | | | | | | | | | | | | | | |

# Table S3. Joanna Briggs Institute (JBI) Critical Appraisal Checklist for non-randomized experimental studies

|  | **House el al** [**[**](#_bookmark34)**62]** | **Kopp el al** [**[67**](#_bookmark31)**]** | **Kirschbaum-Lesch et al** [**[64**](#_bookmark34)**]** | **Papatheodorou** [**[83**](#_bookmark34)**]** | **Ricketts 2022**  [**[69**](#_bookmark34)**]** | **Bromundt et al** [**[72**](#_bookmark34)**]** | **Li el al,** [**[7**](#_bookmark57)**5]** | **Nixon**  **[**[**92**](#_bookmark56)**]** | **Swanson**  [**[74**](#_bookmark34)**]** |
| --- | --- | --- | --- | --- | --- | --- | --- | --- | --- |
| (1) Was the causal relationship in the study clearly described? | Yes | Yes | Yes | Yes | Yes | Yes | Yes | Yes | Yes |
| (2) Was the baseline between groups comparable? | Yes | Yes | Yes | Yes | Unclear | Yes | Unclear | Yes | Yes |
| (3) In addition to the intervention measures to be verified, were other measures accepted by each group the same? | Yes | Yes | Yes | Unclear | Yes | Yes | Yes | Unclear | Yes |
| (4) Was there a control group? | Yes | Yes | Yes | Yes | Yes | Yes | Yes | Yes | Yes |
| (5) Was diversified measurement on outcome indicators conducted before and after intervention? | Yes | Yes | Yes | Yes | Yes | Yes | Yes | Yes | Yes |
| (6) Was the follow-up complete, if not, was the lost follow-up reported or treated properly? | Yes | Yes | Yes | Yes | Yes | Yes | Yes | Yes | Yes |
| (7) Were the outcome indicators of each group to evaluated in the same way? | Yes | Yes | Yes | Yes | Yes | Yes | Yes | Yes | Yes |
| (8) Was the evaluation method of outcome indicators reliable? | Yes | Yes | Yes | Yes | Yes | Yes | Yes | Yes | Yes |
| (9) Was the data analysis method appropriate? | Yes | Yes | Yes | Yes | Yes | Yes | Yes | Yes | Yes |

# Table S4. Meta-regression with potential covariates of main heterogeneity.

| **Subgroup/ Covariates** | **Meta-regression results** | **Meta-regression plots** |
| --- | --- | --- |
| Follow-up vs intervention | Meta-regression Number of obs = 97  REML estimate of between-study variance tau2 = 2.595  % residual variation due to heterogeneity I-squared_res = 92.89%  Proportion of between-study variance explained Adj R-squared = -0.64%  With Knapp-Hartung modification  ------------------------------------------------------------------------------  _ES \| Coefficient Std. err. t P>\|t\| [95% conf. interval]  -------------+----------------------------------------------------------------  follow1 \| -0.320 0.434 -0.74 0.462 -1.181 0.541  _cons \| -1.548 0.192 -8.07 0.000 -1.929 -1.168  ---------------------------------------------------------------------------- | 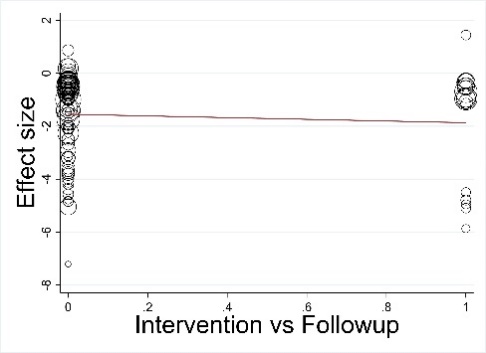 |
| Light color | Meta-regression Number of obs = 97  REML estimate of between-study variance tau2 = 2.579  % residual variation due to heterogeneity I-squared_res = 92.66%  Proportion of between-study variance explained Adj R-squared = -0.03%  With Knapp-Hartung modification  ------------------------------------------------------------------------------  _ES \| Coefficient Std. err. t P>\|t\| [95% conf. interval]  -------------+----------------------------------------------------------------  color1 \| 0.333 0.355 0.94 0.350 -0.371 1.037  _cons \| -1.736 0.217 -7.99 0.000 -2.167 -1.305  ------------------------------------------------------------------------------ | 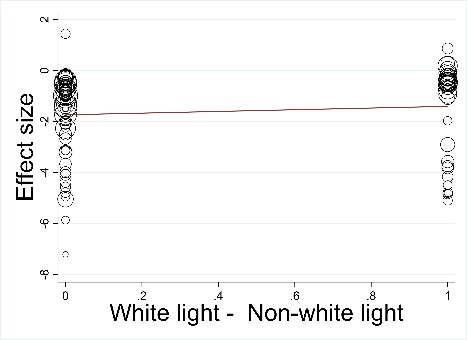 |
| RCT vs quasi-experimental | Meta-regression Number of obs = 97  REML estimate of between-study variance tau2 = 2.56  % residual variation due to heterogeneity I-squared_res = 92.83%  Proportion of between-study variance explained Adj R-squared = 0.70%  With Knapp-Hartung modification  ------------------------------------------------------------------------------  _ES \| Coefficient Std. err. t P>\|t\| [95% conf. interval]  -------------+----------------------------------------------------------------  type2 \| -0.675 0.515 -1.31 0.193 -1.698 0.348  _cons \| -1.020 0.482 -2.12 0.037 -1.976 -0.064  ------------------------------------------------------------------------------ | 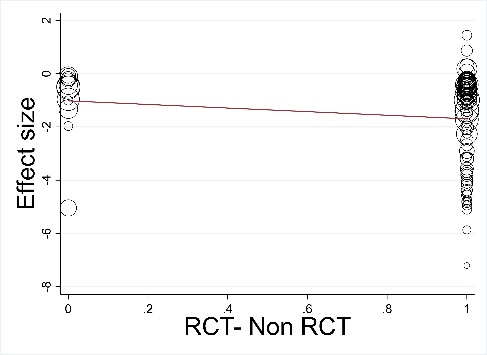 |
| CS_t,f_ ＜0.2 vs ＞0.2 | Meta-regression Number of obs = 97  REML estimate of between-study variance tau2 = 2.603  % residual variation due to heterogeneity I-squared_res = 92.89%  Proportion of between-study variance explained Adj R-squared = -0.94%  With Knapp-Hartung modification  ------------------------------------------------------------------------------  _ES \| Coefficient Std. err. t P>\|t\| [95% conf. interval]  -------------+----------------------------------------------------------------  CStf1 \| -0.184 0.380 -0.48 0.629 -0.938 0.570  _cons \| -1.558 0.204 -7.62 0.000 -1.964 -1.152  ------------------------------------------------------------------------------ | 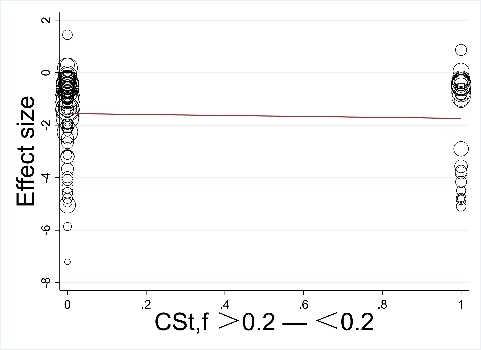 |
| Light Intensity ＜3000lx vs ＞3000lx | Meta-regression Number of obs = 97  REML estimate of between-study variance tau2 = 2.578  % residual variation due to heterogeneity I-squared_res = 92.71%  Proportion of between-study variance explained Adj R-squared = 0.02%  With Knapp-Hartung modification  ------------------------------------------------------------------------------  _ES \| Coefficient Std. err. t P>\|t\| [95% conf. interval]  -------------+----------------------------------------------------------------  intensity1 \| 0.345 0.348 0.99 0.325 -0.347 1.036  _cons \| -1.754 0.224 -7.82 0.000 -2.199 -1.308  ------------------------------------------------------------------------------ | 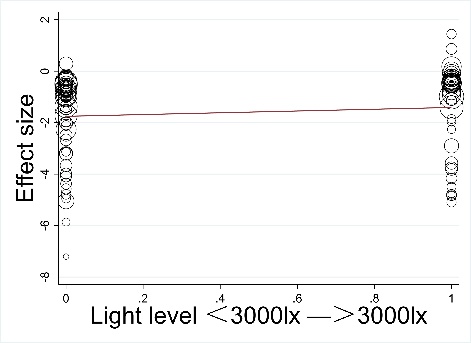 |
| Medicated vs non-medicated***** | Meta-regression Number of obs = 85  REML estimate of between-study variance tau2 = 2.512  % residual variation due to heterogeneity I-squared_res = 93.25%  Proportion of between-study variance explained Adj R-squared = 6.94%  With Knapp-Hartung modification  ------------------------------------------------------------------------------  _ES \| Coefficient Std. err. t P>\|t\| [95% conf. interval]  -------------+----------------------------------------------------------------  medication1 \| -1.063 0.401 -2.65  **0.010*** -1.860 -0.266  _cons \| -1.030 0.339 -3.03 0.003 -1.704 -0.355  ------------------------------------------------------------------------------ | 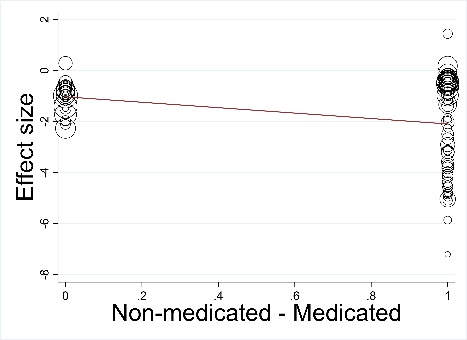 |
| Moderate to severe vs mild to moderate | Meta-regression Number of obs = 85  REML estimate of between-study variance tau2 = 2.697  % residual variation due to heterogeneity I-squared_res = 93.32%  Proportion of between-study variance explained Adj R-squared = 0.08%  With Knapp-Hartung modification  ------------------------------------------------------------------------------  _ES \| Coefficient Std. err. t P>\|t\| [95% conf. interval]  -------------+----------------------------------------------------------------  severe1 \| 0.515 0.467 1.10 0.273 -0.414 1.443  _cons \| -1.898 0.209 -9.08 0.000 -2.313 -1.482  ------------------------------------------------------------------------------ | 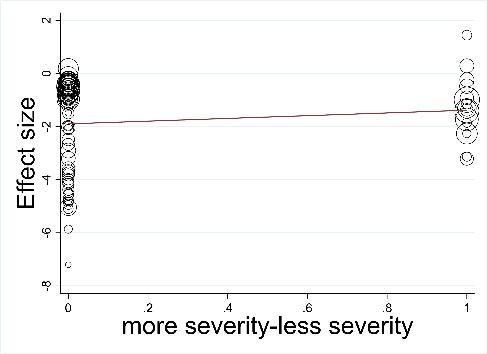 |
| Accumulative exposure time***** | Meta-regression Number of obs = 97  REML estimate of between-study variance tau2 = 2.188  % residual variation due to heterogeneity I-squared_res = 92.42%  Proportion of between-study variance explained Adj R-squared = 15.14%  Joint test for all covariates Model F(4,92) = 4.79  With Knapp-Hartung modification Prob > F = 0.0015  ------------------------------------------------------------------------------  _ES \| Coefficient Std. err. t P>\|t\| [95% conf. interval]  -------------+----------------------------------------------------------------  minute1 \| -0.261 0.591 -0.44 0.660 -1.436 0.913  minute2 \| -2.218 0.598 -3.71 **0.000***  -3.406 -1.030  minute3 \| -0.441 0.533 -0.83 0.411 -1.499 0.618  minute4 \| -1.021 0.528 -1.93 0.056 -2.070 0.028  _cons \| -0.815 0.437 -1.87 0.065 -1.682 0.052  ------------------------------------------------------------------------------ | — |
| *p＜0.05 | | |

# Table S5. Subgroup meta-analysis and significant between-group difference with potential heterogeneity sources

| **Subgroups** | **study n** | **pooled SMD, Random (95% CI)** | **z test (p^1^)** | **I^2^** | **Q statistic (P^2^)** | **H statistic (95% CI, p^3^)** | **Q statistic (p^4^)** | **H statistic, (95% CI)** |
| --- | --- | --- | --- | --- | --- | --- | --- | --- |
| ***Whether intervention administered as adjunctive to antidepressant*** | | | | | | | | |
| *LT for depressed with antidepressants* | 18 | -2.10 (-2.50, -1.68) | -9.991** | 94.8% | 1149.48** | 4.4 (4.0,4.7) ** | 42.46 (p=0.000**) | 4.6 (3.1,6.9) |
| *LT for depressed with no antidepressants* | 6 | -1.03 (-1.270, -0.78) | -8.283** | 64.5% | 64.71** | 1.7 (1.3,2.1) ** |  |  |
| *Non-depressed, non-medicated* | 7 | -0.398 (-0.700, -0.095) | -2.574* | 60.9% | 28.16** | 1.6 (1.2,2.2) ** |  |  |
| ***Maladies severity (among depressed studies)*** | | | | | | | | |
| *Moderate to severe* | 17 | -1.885 (-2.244, -1.526) | -10.294** | 94.0% | 1122.04 ** | 4.1 (3.8, 4.4) | 2.96 (p=0.085) | — |
| *Mild to moderate* | 7 | -1.367 (-1.835, -0.899) | -5.72** | 86.8% | 120.77** | 2.7 (2.3, 3.4) |  |  |
| ***Intensity of light therapy*** | | | | | | | | |
| *Low intensity (<3000 lux)* | 16 | -1.387 (-1.799, -0.975) | -6.600** | 92.9% | 550.80** | 3.8 (3.4,4.2) ** | 1.58 (p=0.208) | — |
| *High intensity (>3000 lux)* | 24 | -1.734 (-2.083, -1.384) | -9.724** | 92.4% | 739.10** | 3.6 (3.3,4.0) ** |  |  |
| ***Light color­*** | | | | | | | | |
| *White light* | 23 | -1.717 (-2.043, -1.391) | -10.312** | 91.8% | 734.26** | 3.5 (3.2,3.8) ** | 1.38 (p=0.241) | — |
| *Non-white light* | 14 | -1.383 (-1.835, -0.932) | -6.009** | 93.6% | 544.39** | 3.9 (3.5,4.4) ** |  |  |
| ***Accumulative exposure time during intervention (no follow-up)*** | | | | | | | | |
| *0-300mins* | 5 | -1.073 (-1.615, -0.531) | -3.880** | 91.2% | 158.48** | 3.4 (2.8,4.1) ** | 9.79 (p=0.044*) | 1.6 (1.0,2.6) |
| *300-500min* | 7 | -1.489 (-2.081, -0.897) | -4.930** | 91.8% | 207.67** | 3.5 (3.0,4.1) ** |  |  |
| *500-1000mins* | 7 | -1.975 (-2.558, -1.392) | -6.641** | 93.3% | 373.32** | 3.9 (3.4,4.4) ** |  |  |
| *1000-1500mins* | 6 | -1.935 (-3.098, -0.772) | -3.260** | 95.0% | 140.01** | 4.5 (3.6,5.6) ** |  |  |
| *>1500mins* | 6 | -0.871 (-1.397, -0.344) | -3.241** | 82.6% | 57.31** | 2.4 (1.8,3.1) ** |  |  |
| ***Circadian stimulus*** | | | | | | | | |
| *CS_t,f_ > 0.2* | 31 | -1.534 (-1.835, -1.232) | -9.966** | 92.0% | 850.65** | 3.5 (3.3,3.8) ** | 0.37 (p=0.543) | — |
| *CS_t,f_ < 0.2* | 12 | -1.734 (-2.304, -1.164) | -5.966** | 94.3% | 470.75** | 4.2 (3.7,4.7) ** |  |  |
| ***Effect size during intervention vs follow-up (stabilization)*** | | | | | | | | |
| *During intervention* | 31 | -1.524 (-1.813, -1.234) | -10.328** | 92.3% | 1003.4** | 3.6 (3.3,3.9 ** | 0.83 (p=0.361) | — |
| *Follow-up* | 8 | -1.879 (-2.584, -1.174) | -5.222** | 94.3% | 317.92** | 4.2 (3.6,4.9) ** |  |  |
| ***Experiment design*** | | | | | | | | |
| *RCT studies* | 22 | -1.676 (-1.964, -1.388) | -11.392** | 92.5% | 1117.54** | 3.6 (3.4,3.9) ** | 2.63 (p=0.105) | — |
| *Quasi-experimental studies* | 9 | -1.017 (-1.760, -0.274) | -2.683** | 94.3% | 192.59** | 4.2 (3.5,5.0) ** |  |  |
| CI, confidence interval; **p<0.01; *p<0.05; P1 is the P value of effect size test; P2 is the P value referring to within-subgroup heterogeneity using Cochran’s Q statistic; P3 is the P value within subgroup referring to heterogeneity using H statistic; P4 is the P value between subgroups referring to heterogeneity using Q statistic; | | | | | | | | |

# Supplementary Figures


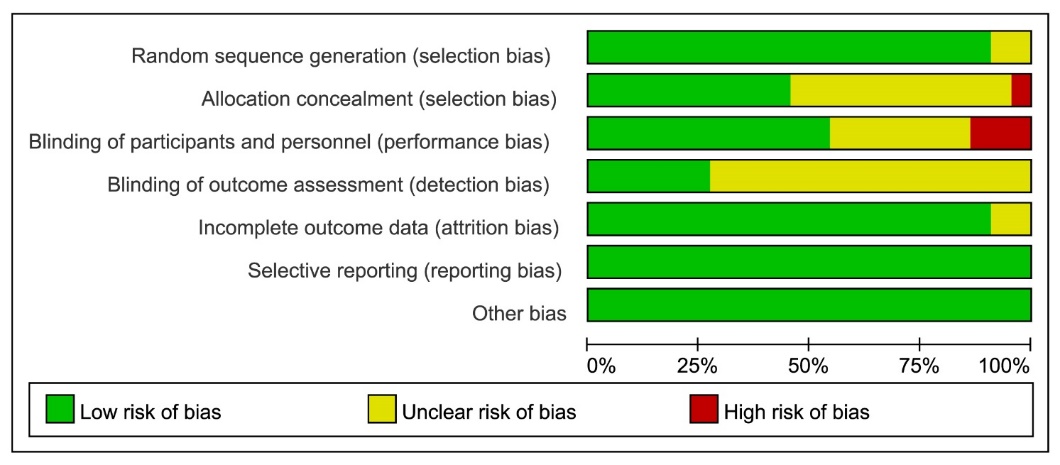


**Supplementary Figure 1(a).** Graphical presentation of overall risk of bias results of included RCTs

**
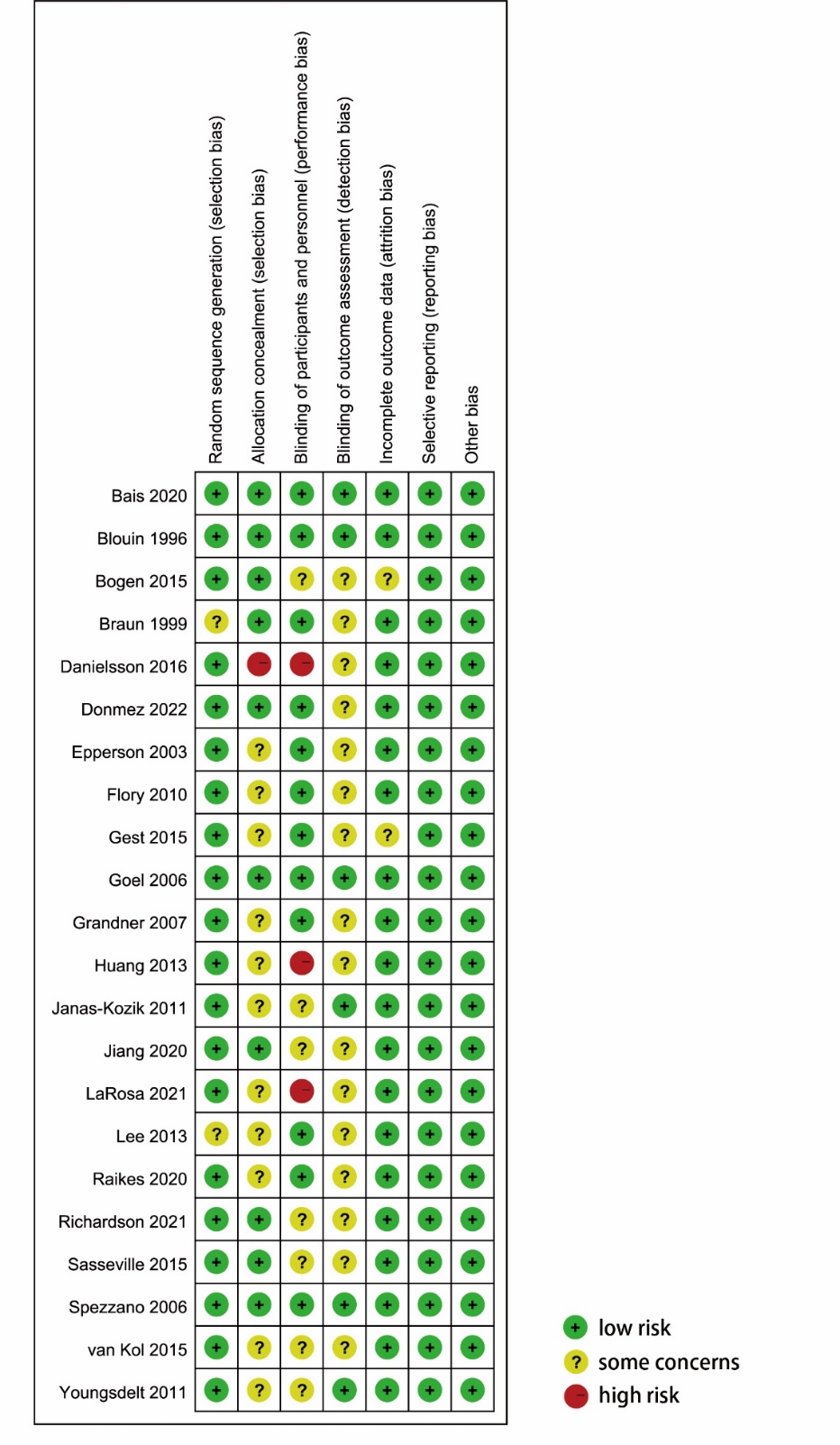
**

**Supplementary Figure 1(b).** Graphical presentation of each risk of bias item for each included RCT


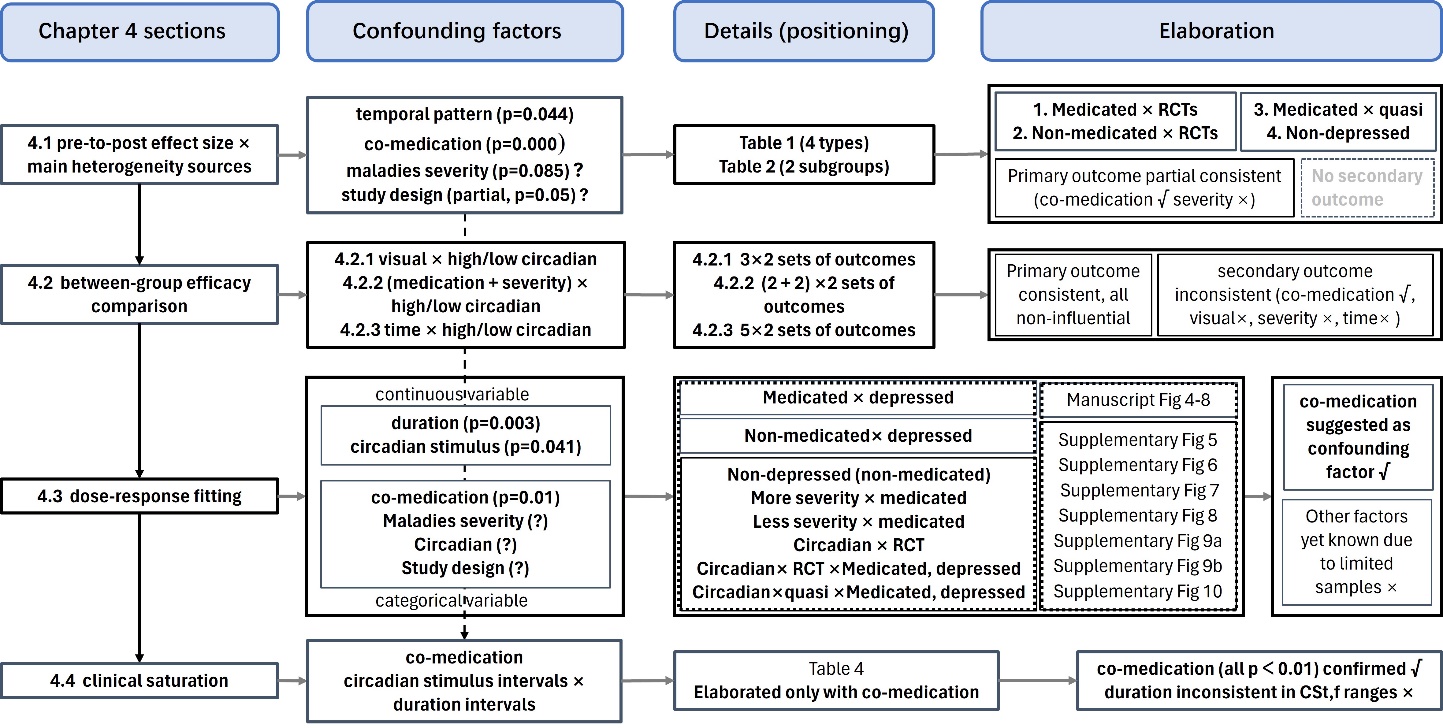


**Supplementary Figure 2.** Main research flow of e.g., between-group efficacy comparison, pre-to-post treatment evaluation and saturation deduction for clinical application


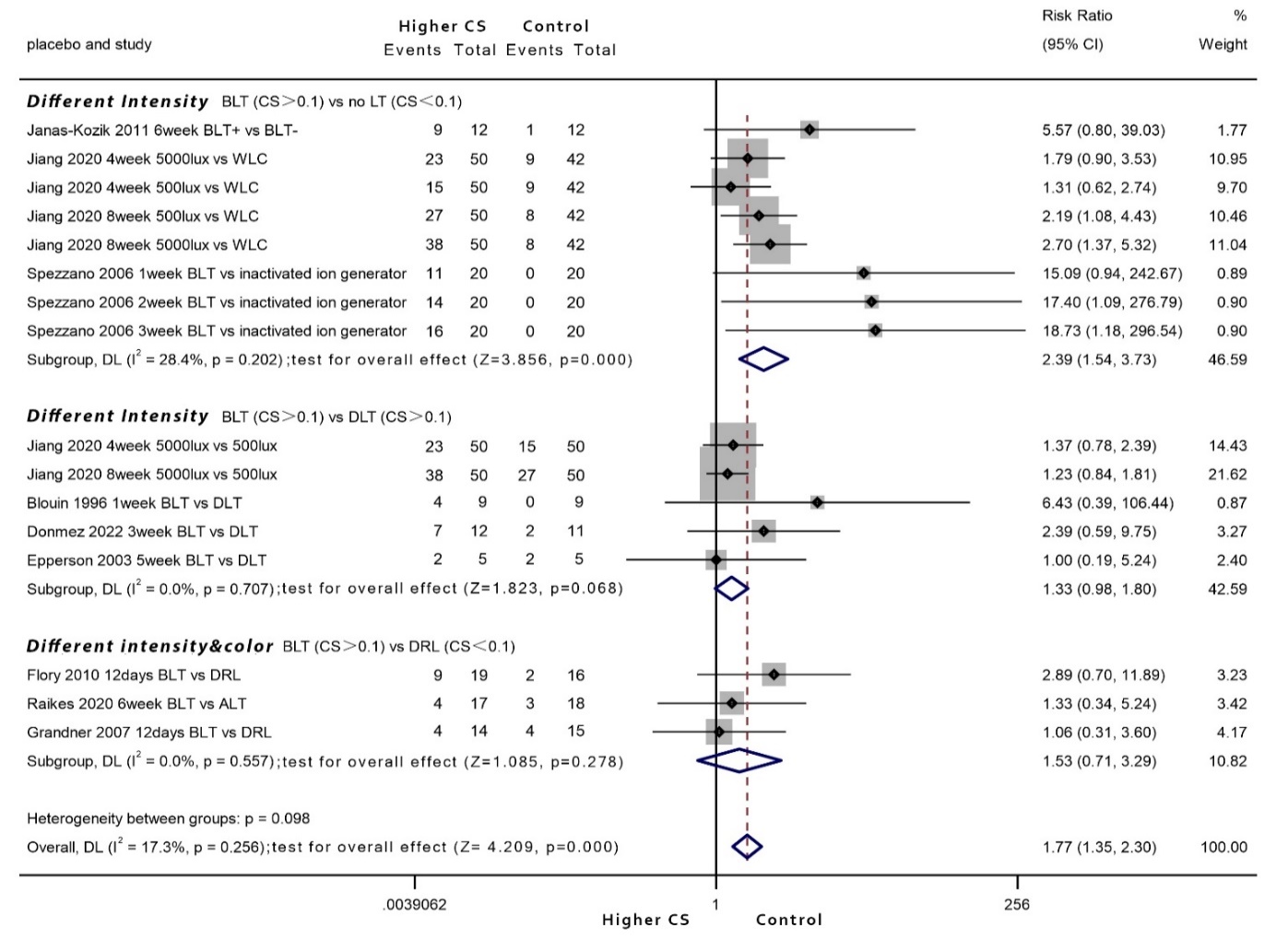


**Supplementary Figure 3 (a).** Forest plot displaying risk ratio (RR) results of between-group meta-analysis of bright light therapy (higher circadian stimulus) versus control group subdivided by visual characteristic (n=9 articles).

**
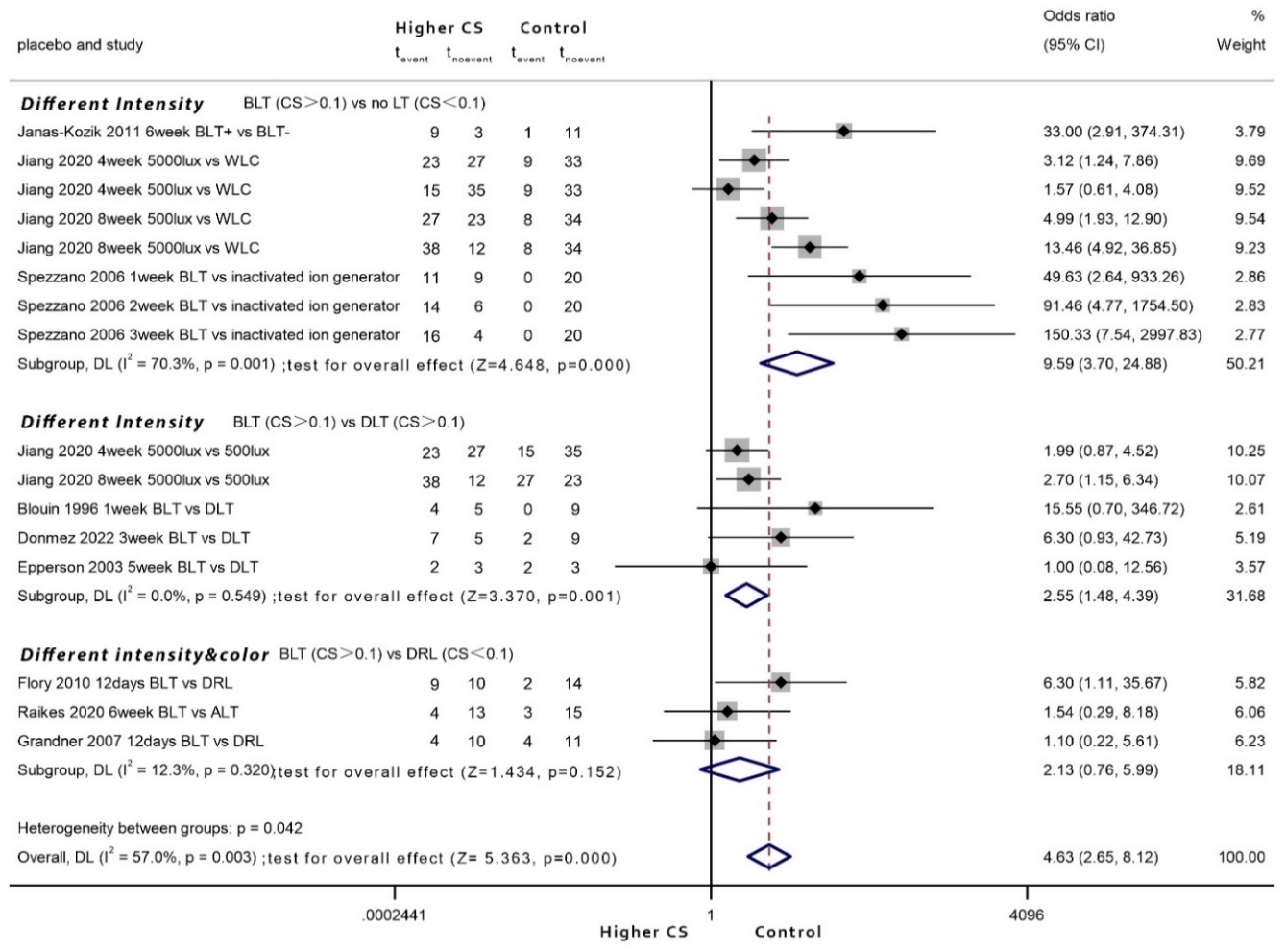
**

**Supplementary Figure 3 (b).** Forest plot displaying odds ratio (OR) results of between-group meta-analysis of bright light therapy (higher circadian stimulus) versus control group subdivided by visual characteristic (n=9 articles).

**
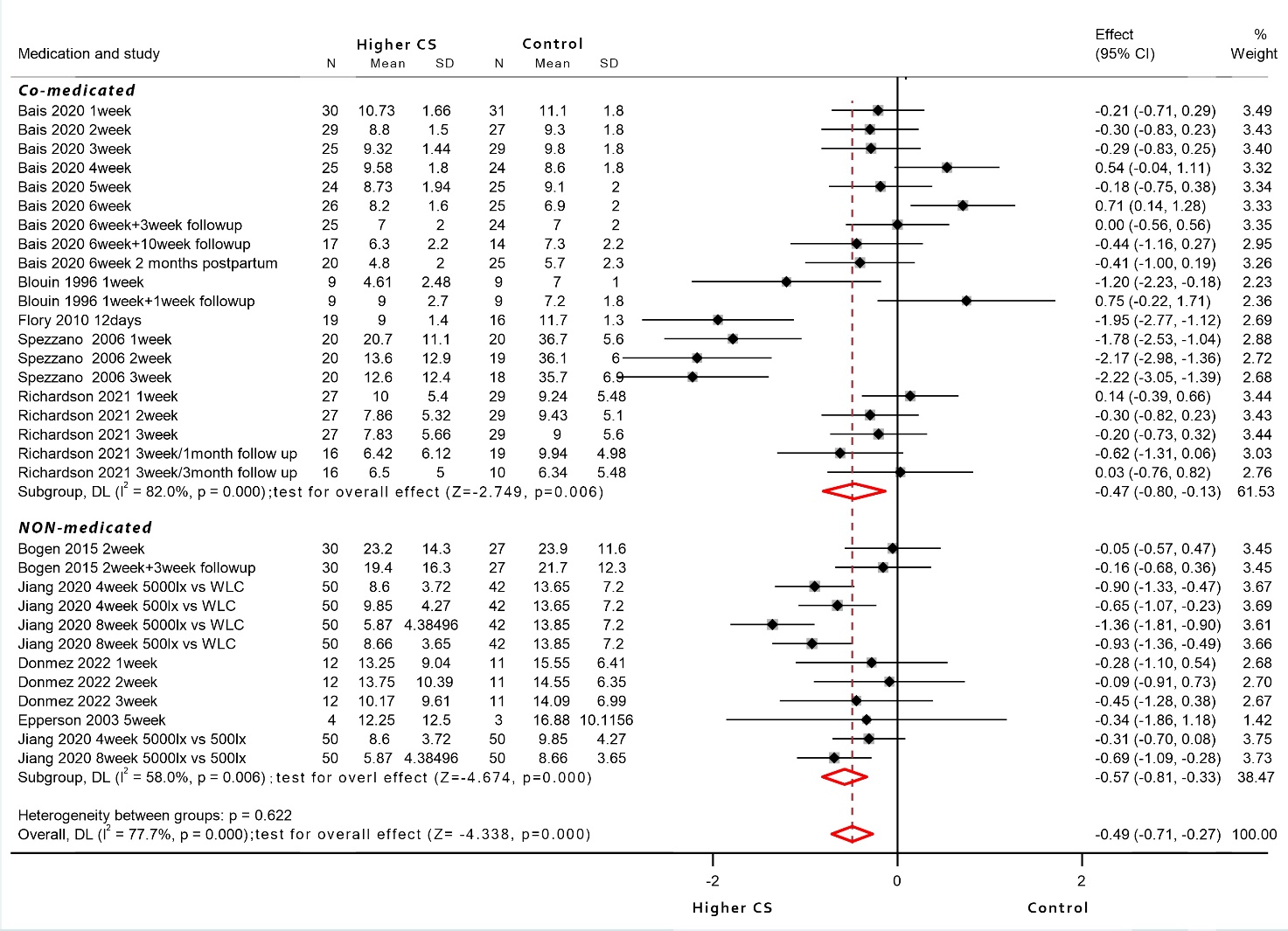
**

**Supplementary Figure 3 (c).** Forest plot displaying primary (SMD) results of between-group meta-analysis of bright light therapy (higher circadian stimulus) versus control group subdivided by co-medication (n=9 articles).


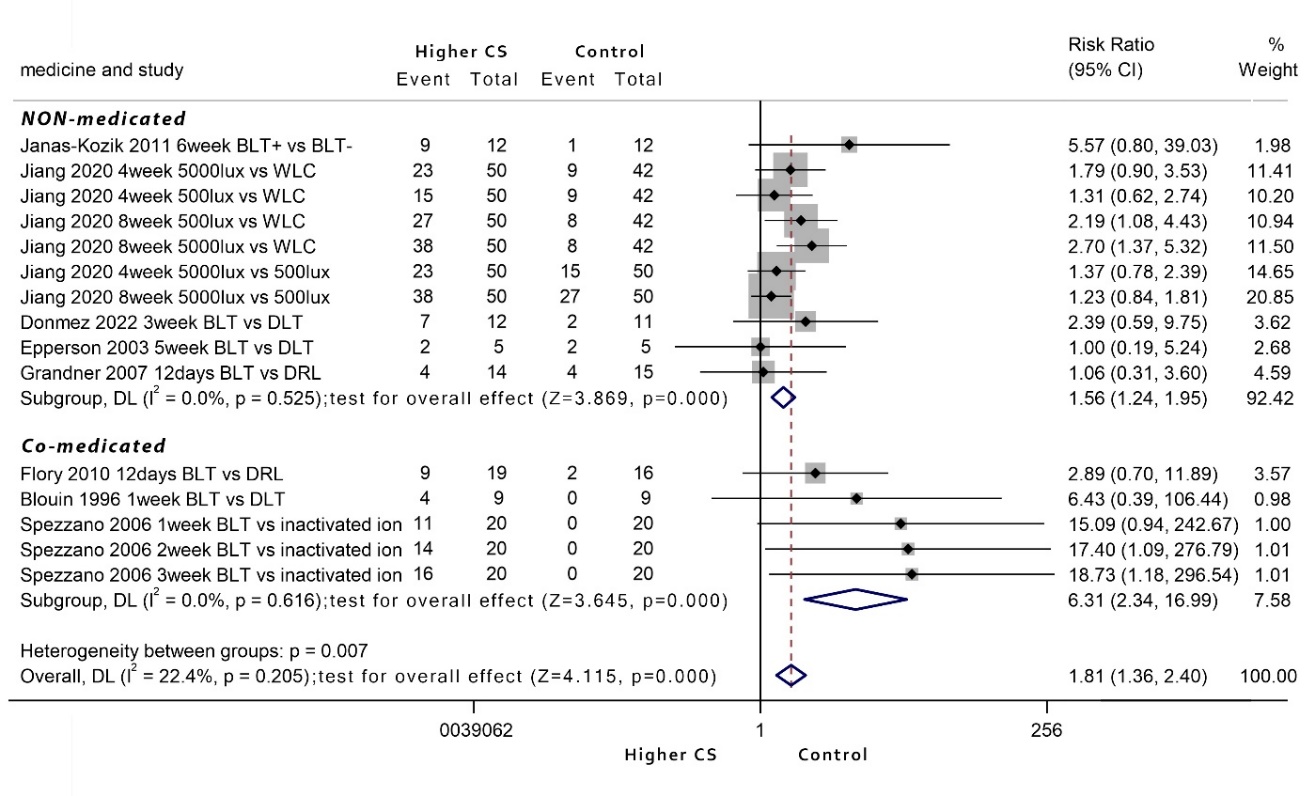


**Supplementary Figure 3 (d).** Forest plot displaying risk ratio (RR) results of between-group meta-analysis of bright light therapy (higher circadian stimulus) versus control group subdivided by co-medication (n=8 articles).


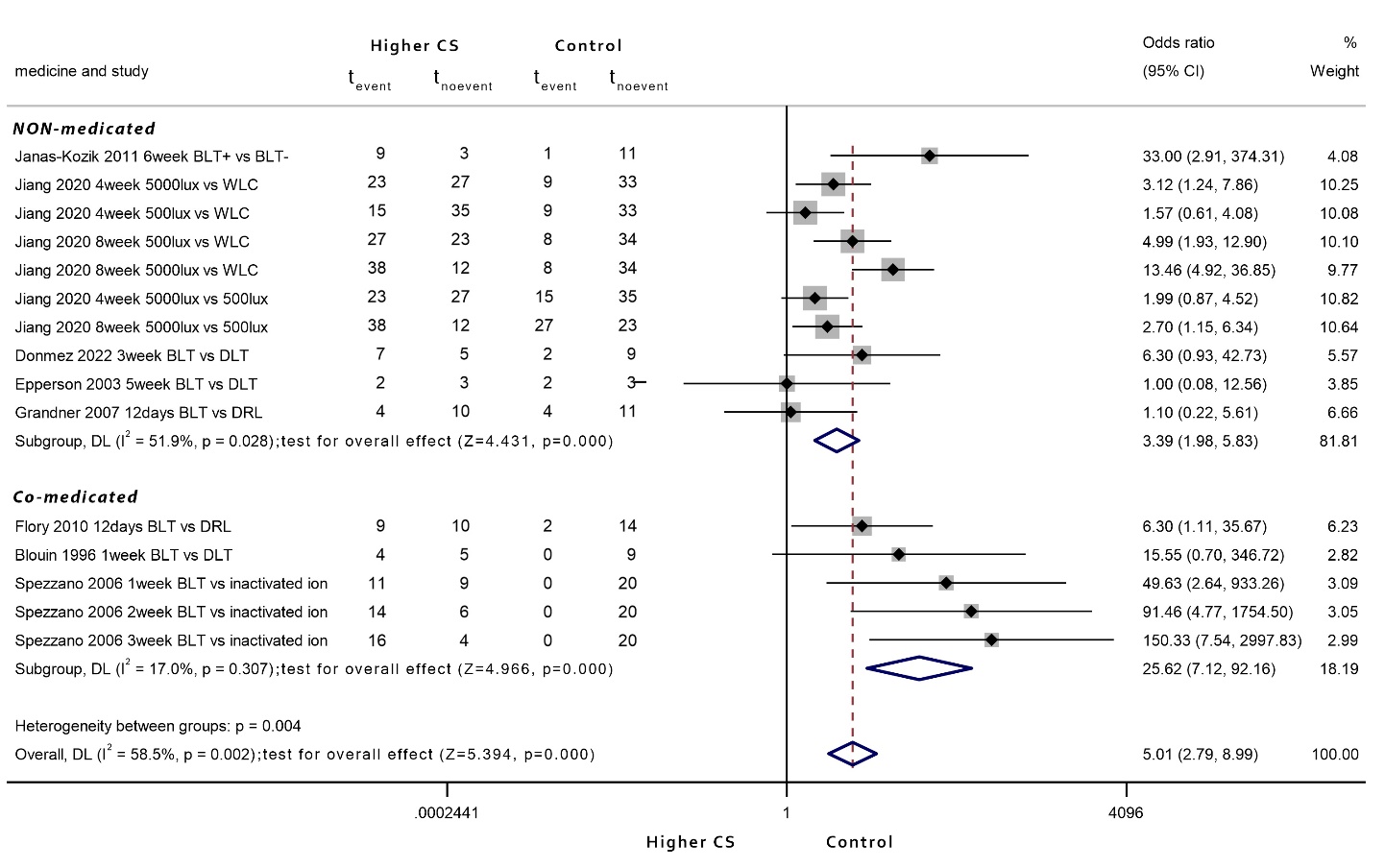


**Supplementary Figure 3 (e).** Forest plot displaying odds ratio (OR) results of between-group meta-analysis of bright light therapy (higher circadian stimulus) versus control group subdivided by co-medication (n=8 articles).


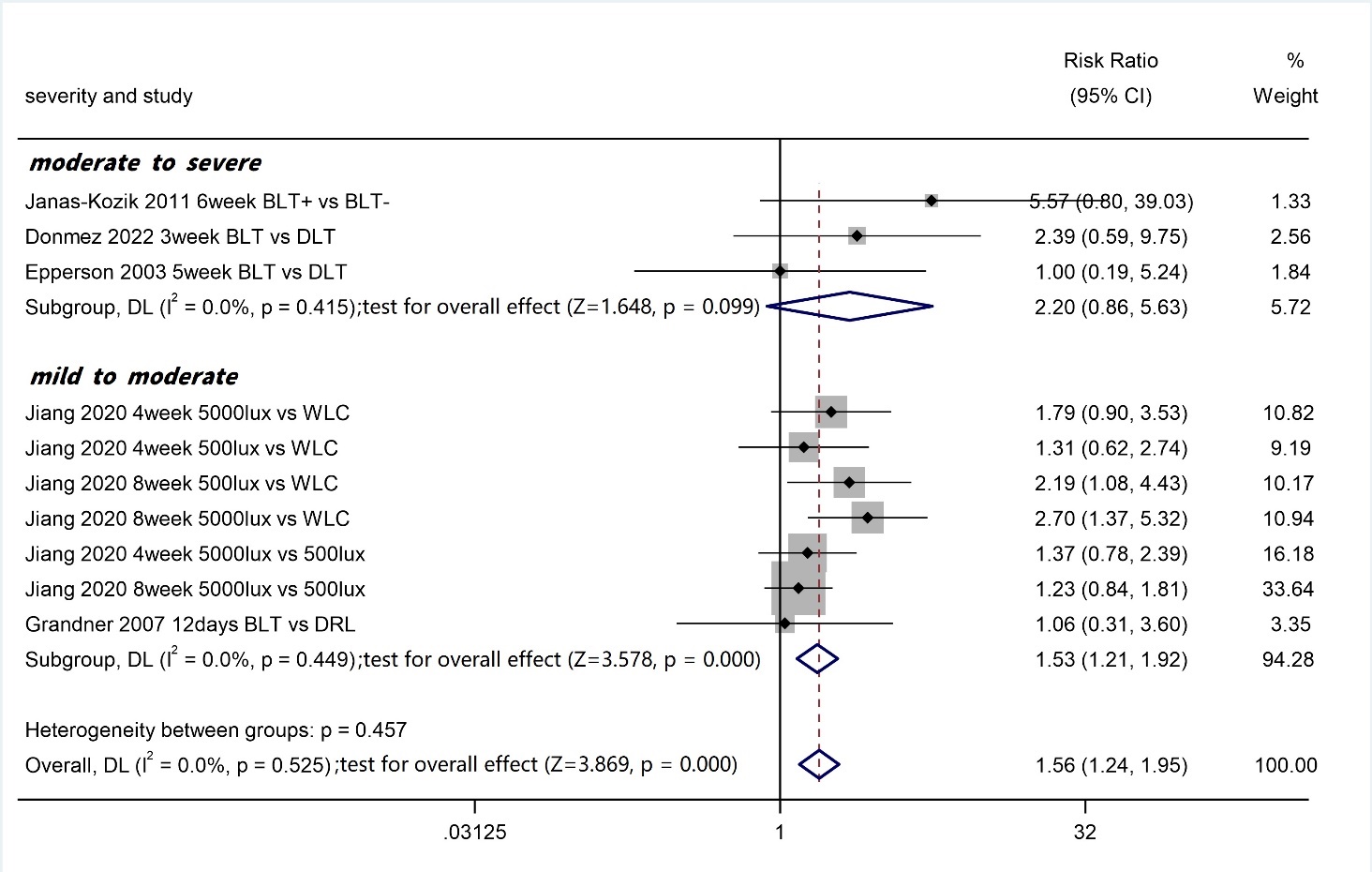


**Supplementary Figure 3 (f).** Forest plot displaying risk ratio (RR) results of between-group meta-analysis of bright light therapy (higher circadian stimulus) versus control group among non-medication studies subdivided by severity (n=5 articles).


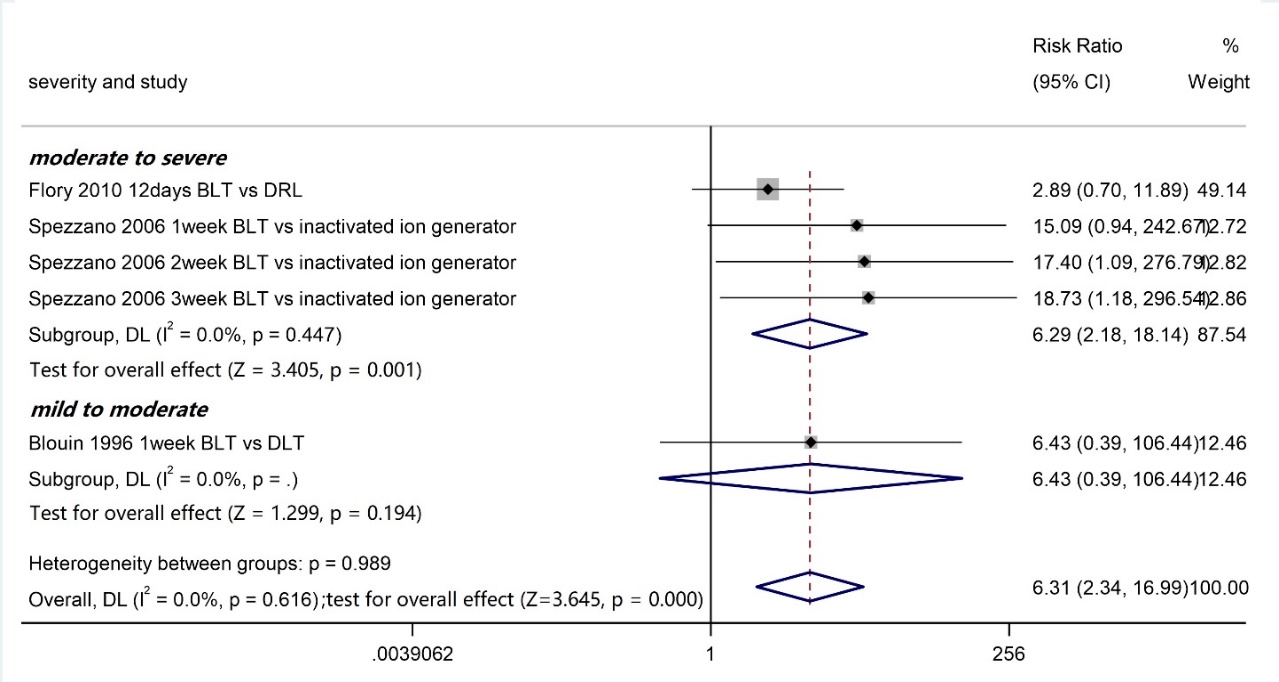


**Supplementary Figure 3 (g).** Forest plot displaying risk ratio (RR) results of between-group meta-analysis of bright light therapy (higher circadian stimulus) versus control group among co-medication studies subdivided by severity (n=3 articles).


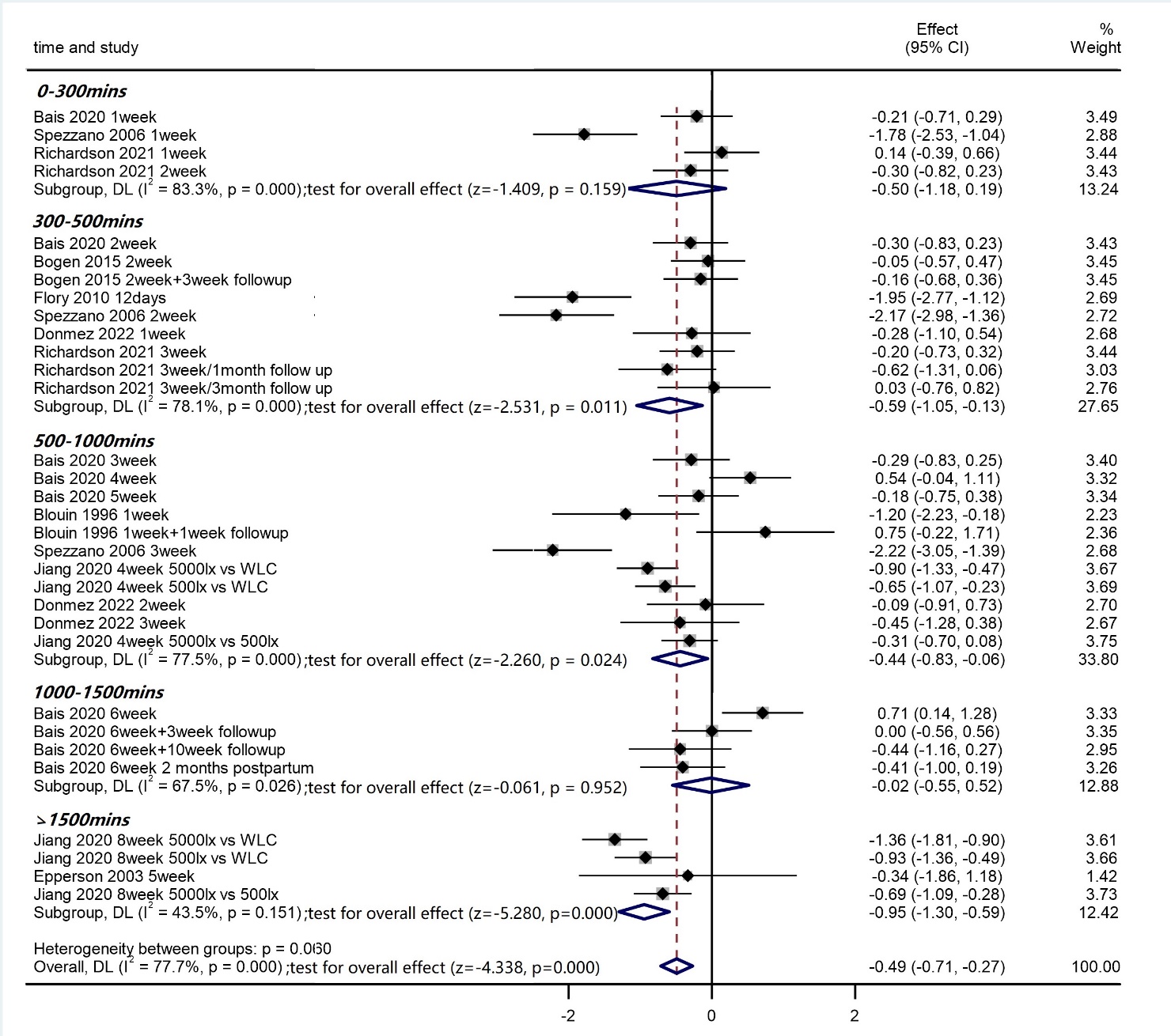


**Supplementary Figure 3 (h).** Forest plot displaying primary (SMD) results of between-group meta-analysis of bright light therapy (higher circadian stimulus) versus control group subdivided by time pattern (n=9 articles).


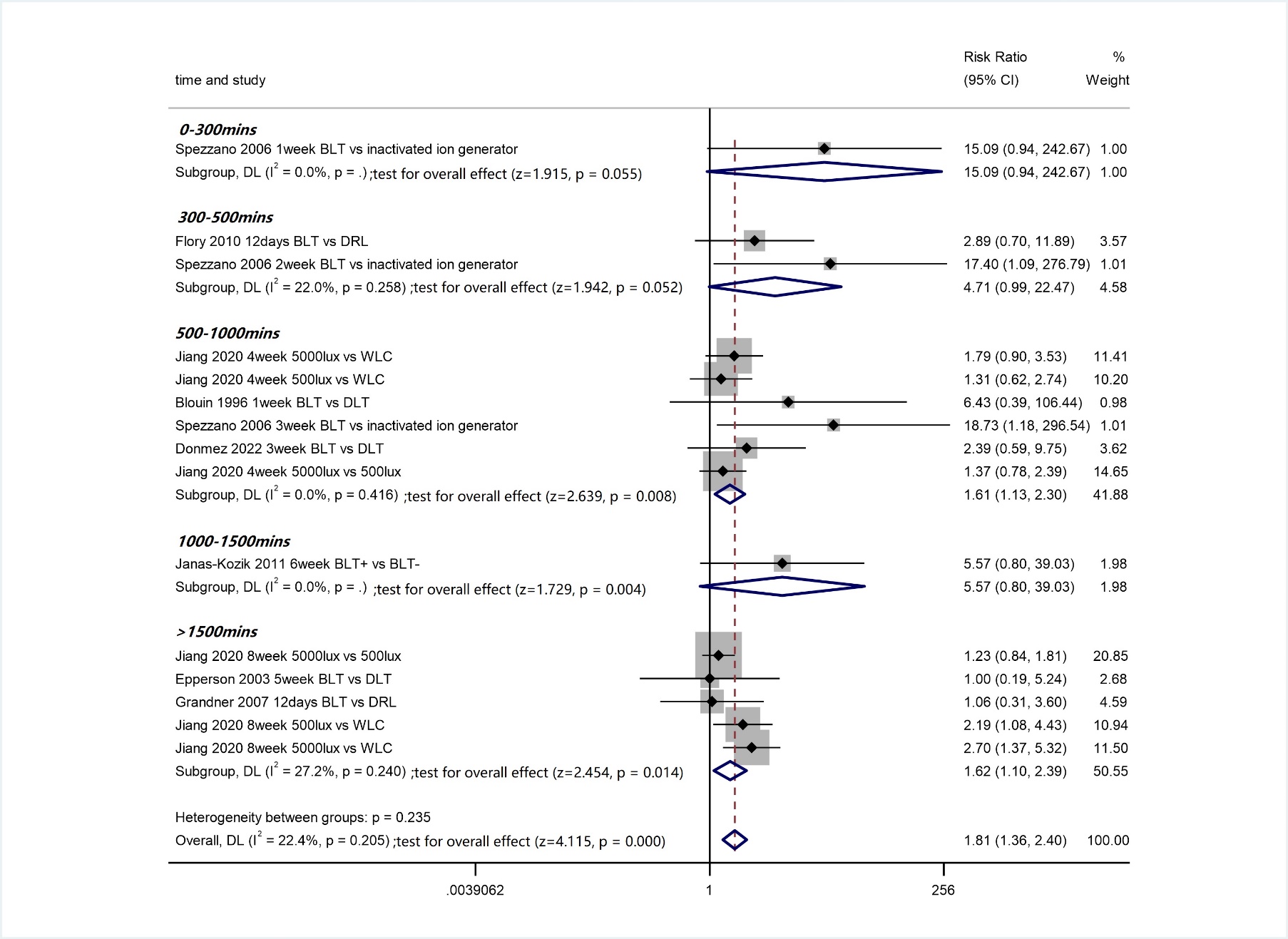


**Supplementary Figure 3 (i).** Forest plot displaying risk ratio (RR) results of between-group meta-analysis of bright light therapy (higher circadian stimulus) versus control group subdivided by time pattern (n=8 articles).


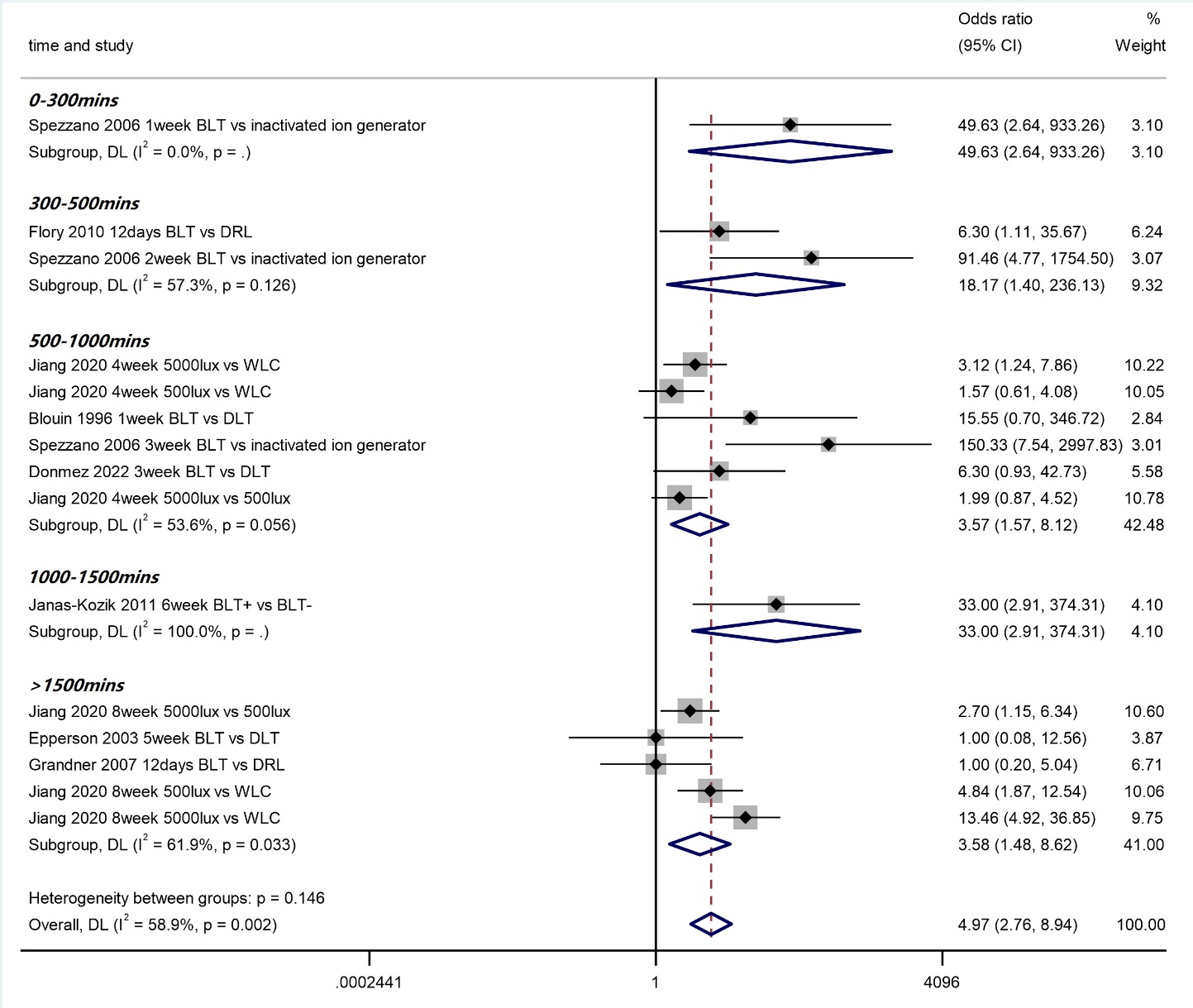


**Supplementary Figure 3 (j).** Forest plot displaying odds ratio (OR) results of between-group meta-analysis of bright light therapy (higher circadian stimulus) versus control group subdivided by time pattern (n=8 articles).

| 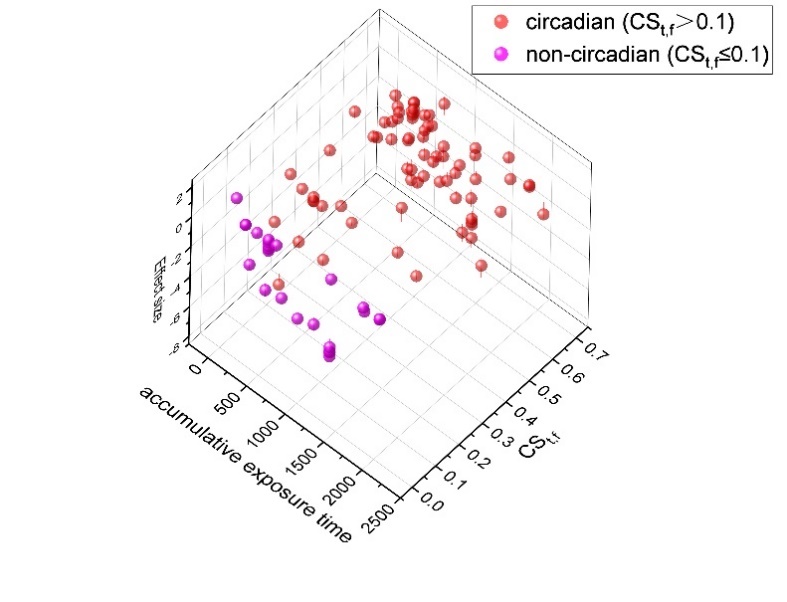 | 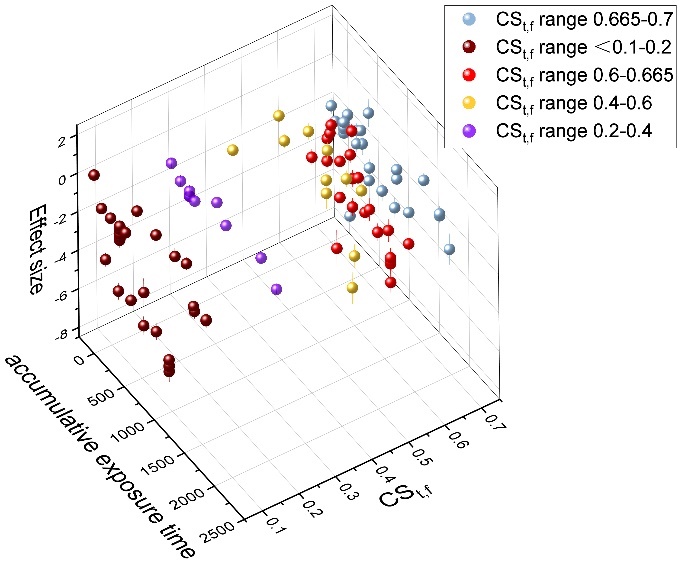 |
| --- | --- |
| (a) **circadian**/non-circadian intervention | (b) various CS_t,f_ ranges |
| 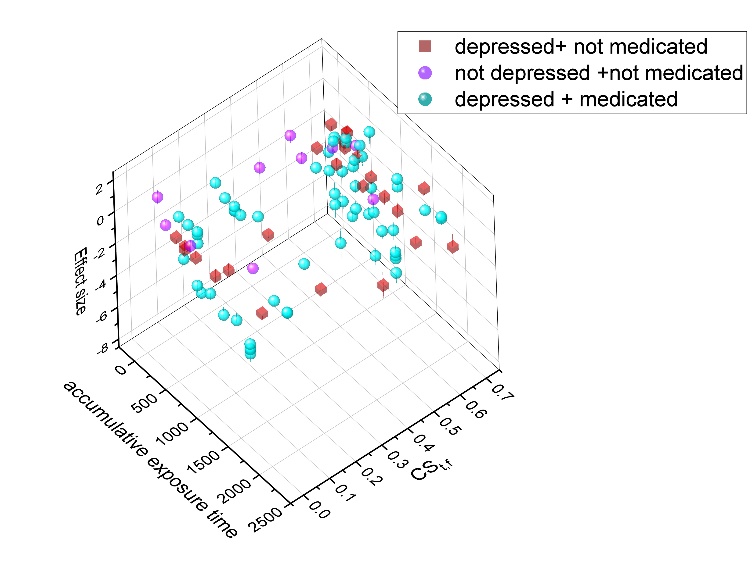 | 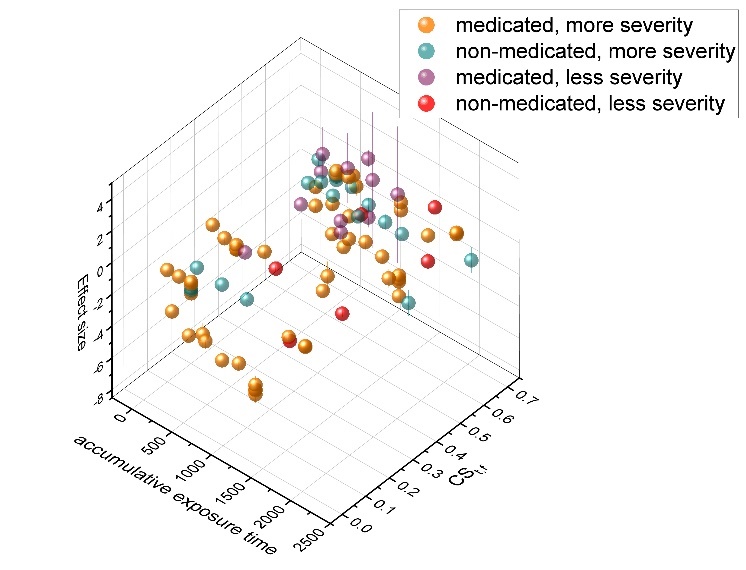 |
| (c) medication/depression condition | (d) more severity/ less severity |
| 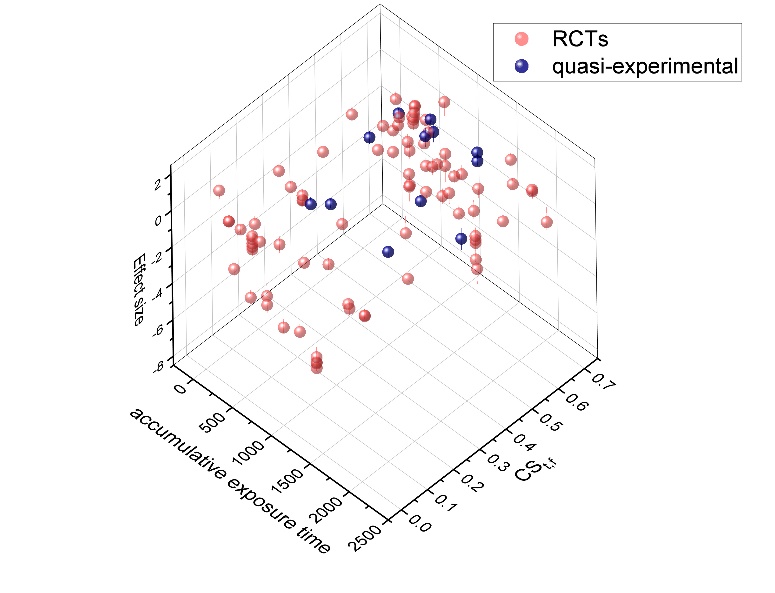 | 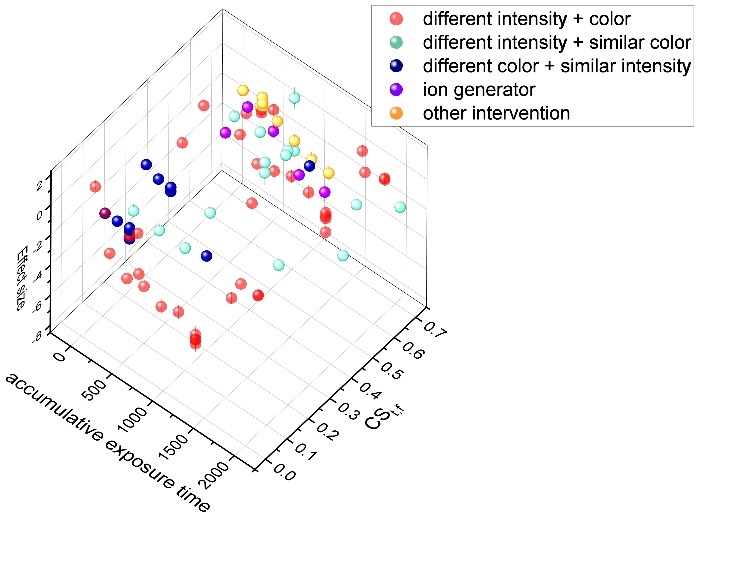 |
| (e) RCTs/non-RCT studies | (f) “visual distinguished” RCT studies |

**Supplementary Figure 4.** Distribution and clusters of data points implying (a) **circadian**/non-circadian intervention, (b) various CS_t,f_ intervals, (c) co-medication, (d) maladies severity, (e) study design, (f) characteristics of RCTs. These are possible dose-response confounding variables, among which, detailed discussion for **(a)** *data points clusters of “circadian” results (73 items, 31 studies),* **(b)** *various CS_t,f_ ranges: the 3D fitting outcome R² of polynomial model, parabola model or power 2D model less convincing with the two variables, but can largely be explained within subgroups (clusters) divided by CS_t,f_ value ranging from＜0.2, 0.2-0.4,0.4-0.6, 0.6-0.665, 0.665-0.7;* **(c)** *the fitting models of medicated/non-medicated people*; **(f)** *between-group difference of “visual distinguished” RCT studies* can be checked in the main body of manuscript, while others were mainly displayed in this supplementary material, with less quantitative conclusions reported due to limited samples.

| 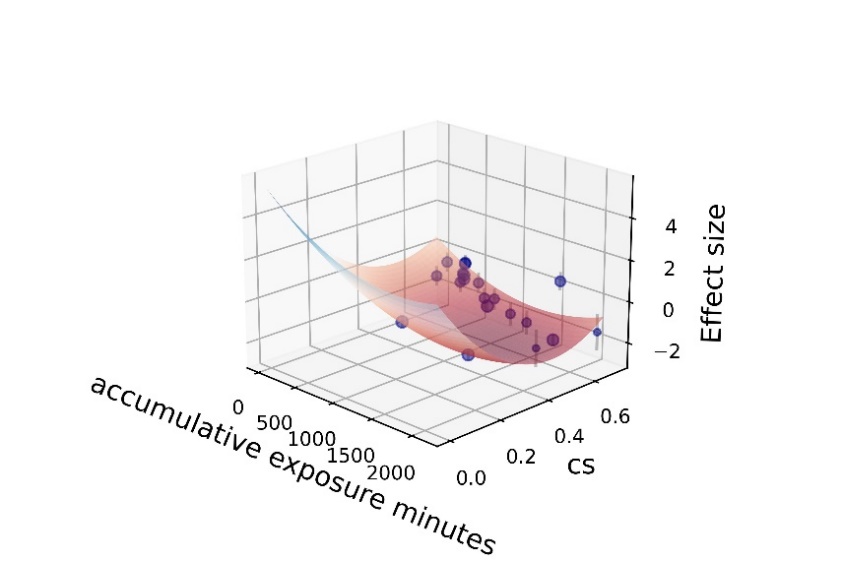 | 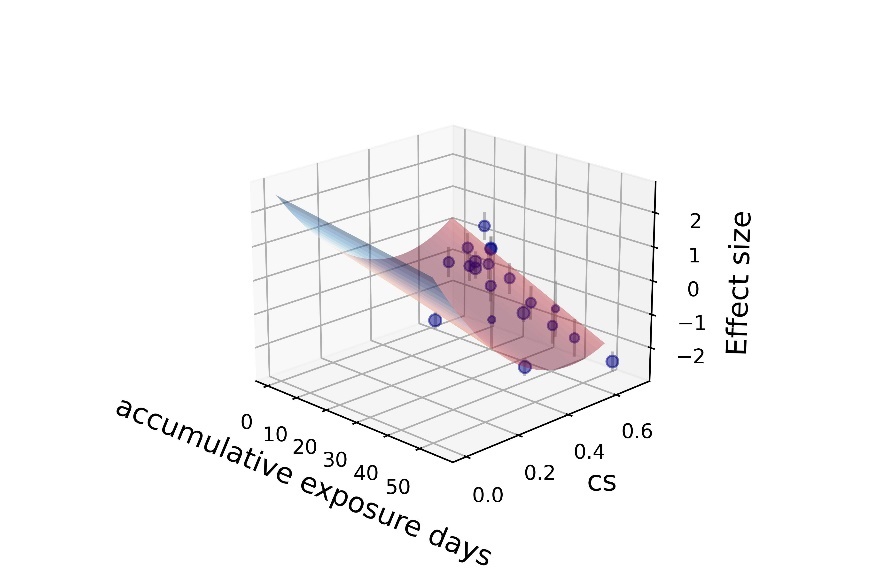 |
| --- | --- |
| 1. poly2D model fitted with accumulative exposure minutes z=5.3919-0.00349x -21.54659y+0.00000105x²+21.0388y² -0.00094xy (R²=23.2%) | 1. poly2D model fitted with accumulative exposure days   z=2.506--0.037x-12.437y+0.00041x²+13.314y²-0.04289xy (R²=75.4%) |
| 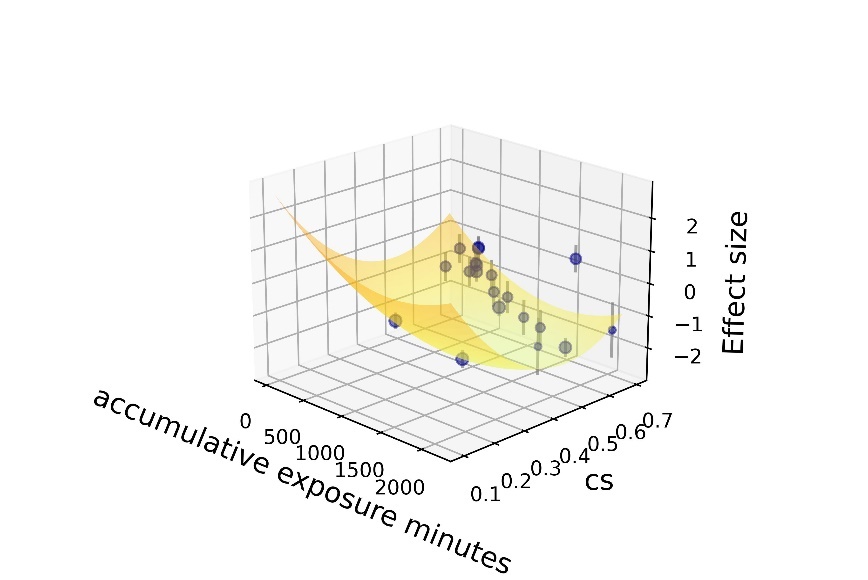 | 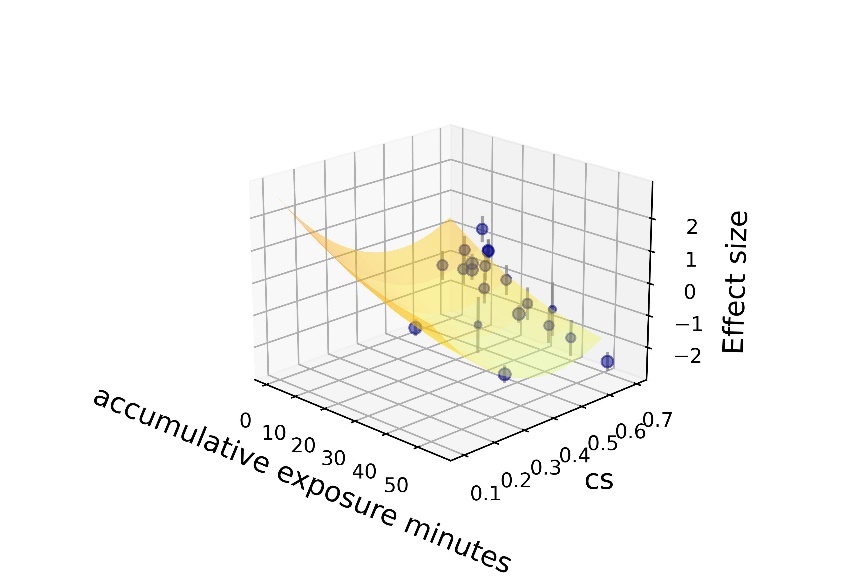 |
| 1. parabola2D model fitted with accumulative exposure minutes z=4.495-0.0026x-19.997y+0.000000893x^2^+20.58y^2^(R²=26.8%) | 1. parabola2D model fitted with accumulative exposure days   z=4.2689-0.07841x-17.099y-0.000666x²+16.567y² (R²=71.9%) |
| 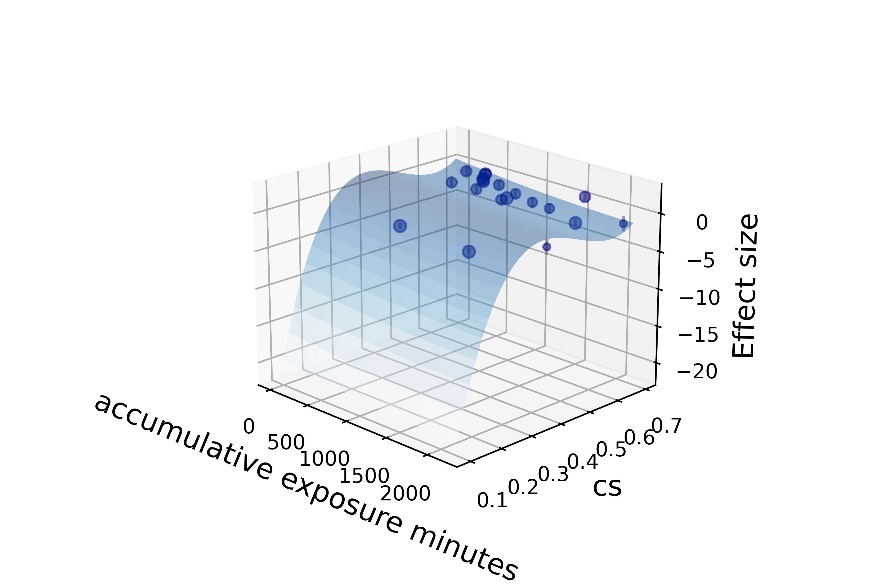 | 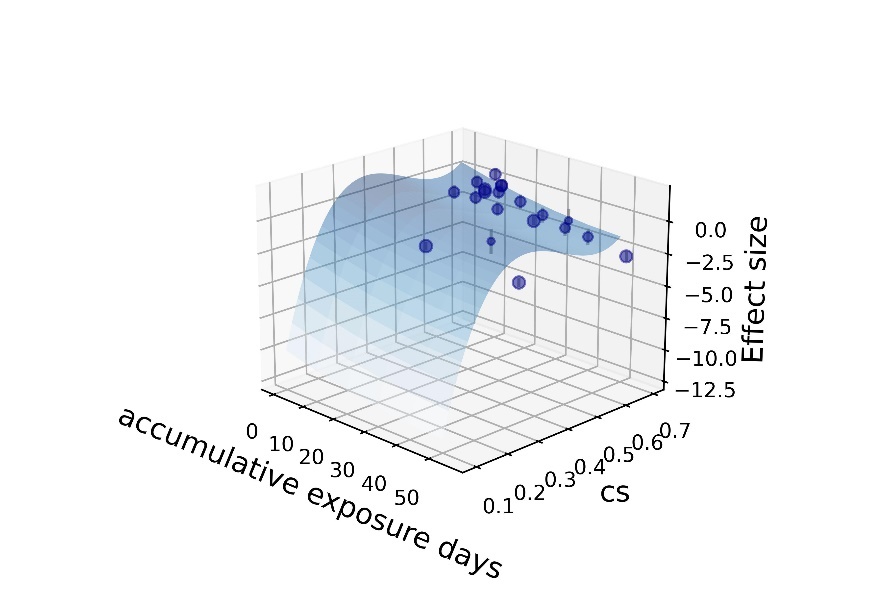 |
| 1. polynormal 2D model fitted with accumulative exposure minutes z=-44.569-0.00143x+0.000975x²+310.16y+0.0000003136x²-661.39y²+444.132y³ (R²=26.1%) | polynormal 2D model fitted with accumulative exposure days   1. z=-23.80-0.076x+0.002x²+172.88y+0.00068x²-375.759y²+255.56y³ (R²=78.3%) |
|  | 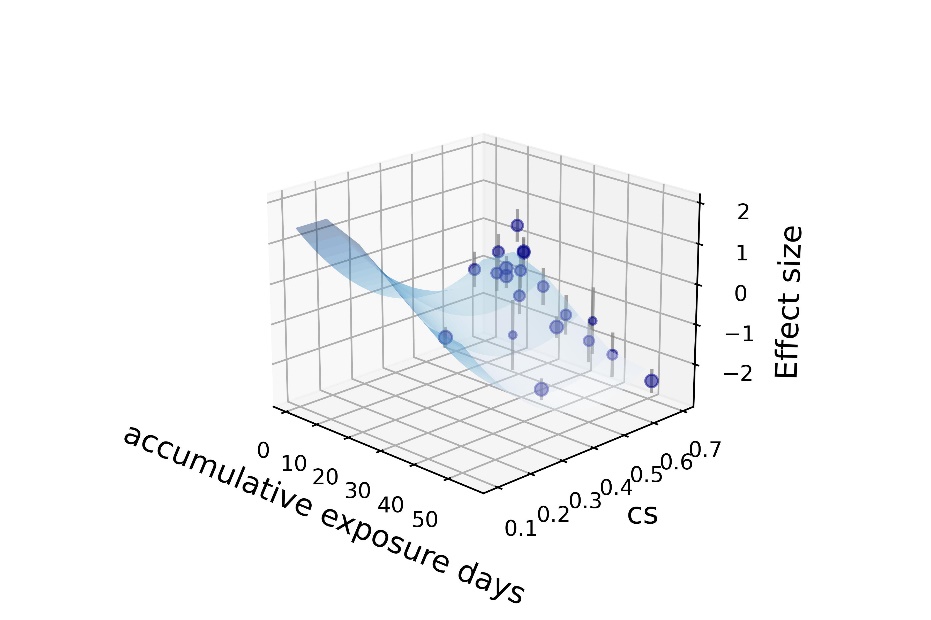 |
|  | 1. polynormal 2D model fitted with accumulative exposure days z=2.8567+0.104x-0.0064x²-0.00007576x³-16.23y+15.677y² (R²=71.3%) |

**Supplementary Figure 5 (a).** Fitting for clusters of data points of 6 studies of unmedicated, depressed people (20 items) on various models

| 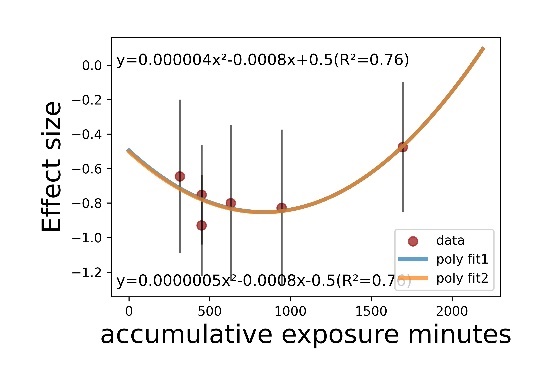 | 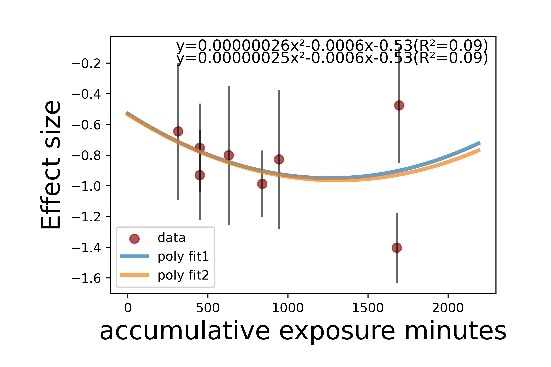 | 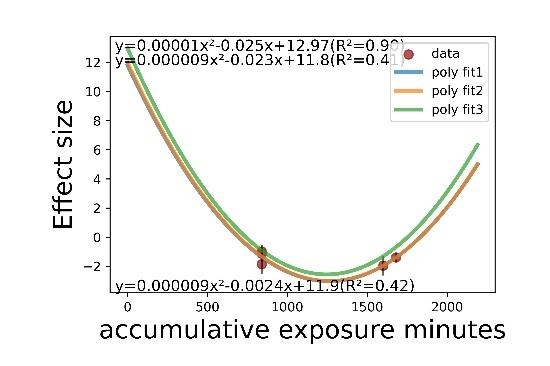 |
| --- | --- | --- |
| CS_t,f_ range ＜0.1, 830mins | CS_t,f_ range ＜0.1-0.4, 1300mins | CS_t,f_ range 0.2-0.6, 1260mins |
| 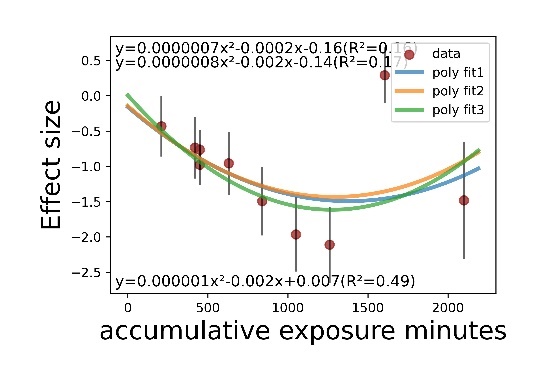 | 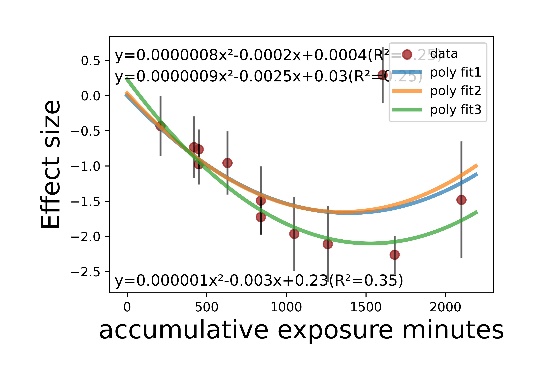 | 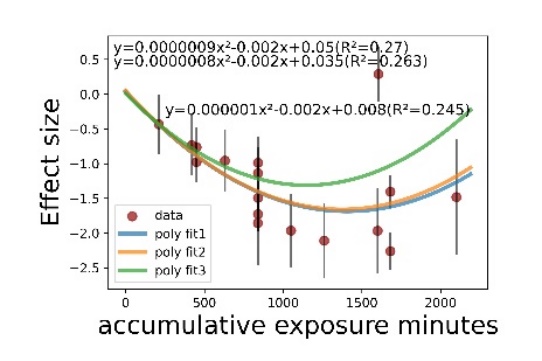 |
| CS_t,f_ range 0.665-0.7, 1300-1400mins | CS_t,f_ range 0.6-0.7, 1400-1500mins | CS_t,f_ range 0.2-0.7, 1100-1400mins |
| **Supplementary Figure 5 (b).** Temporal fitting (accumulative exposure minutes) and saturation among studies without co-medication within various CS_t,f_ ranges | | |
|  | 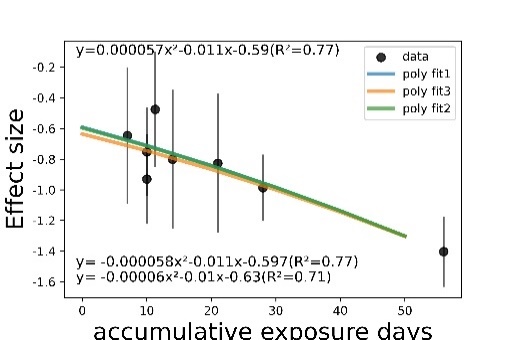 | 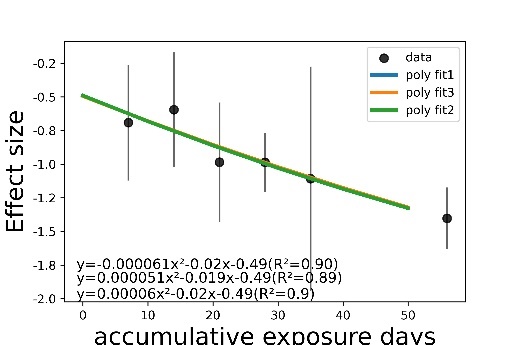 |
|  | CS_t,f_ range ＜0.1-0.4, NA | CS_t,f_ range 0.2-0.6, NA |
| 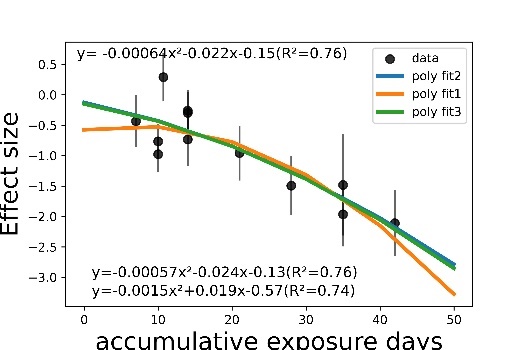 | 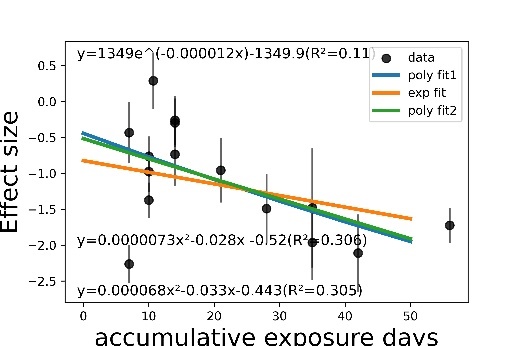 | 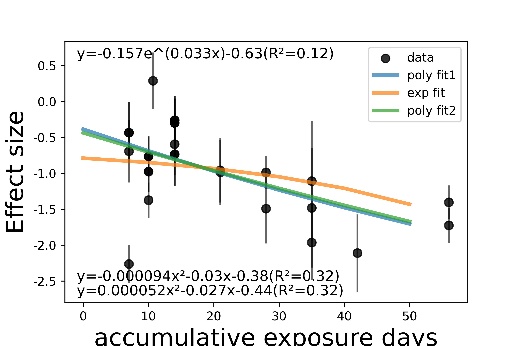 |
| CS_t,f_ range 0.665-0.7, NA | CS_t,f_ range 0.6-0.7, NA | CS_t,f_ range 0.2-0.7, NA |

**Supplementary Figure 5 (c).** Temporal fitting (accumulative exposure days) and saturation among studies without co-medication within various CS_t,f_ ranges

| 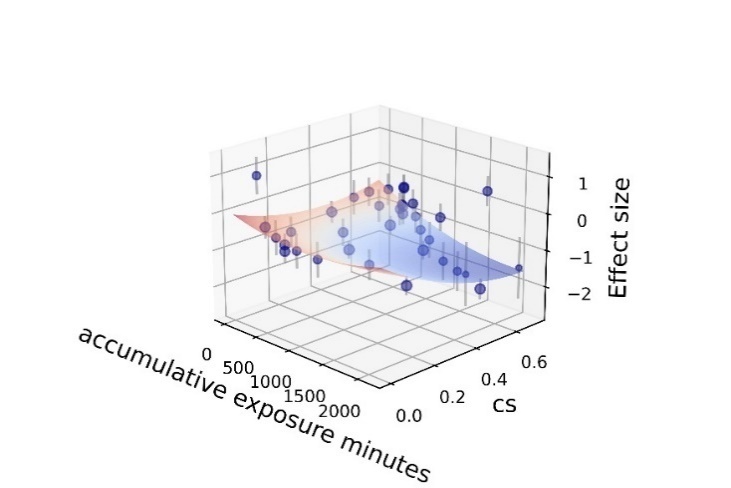 | 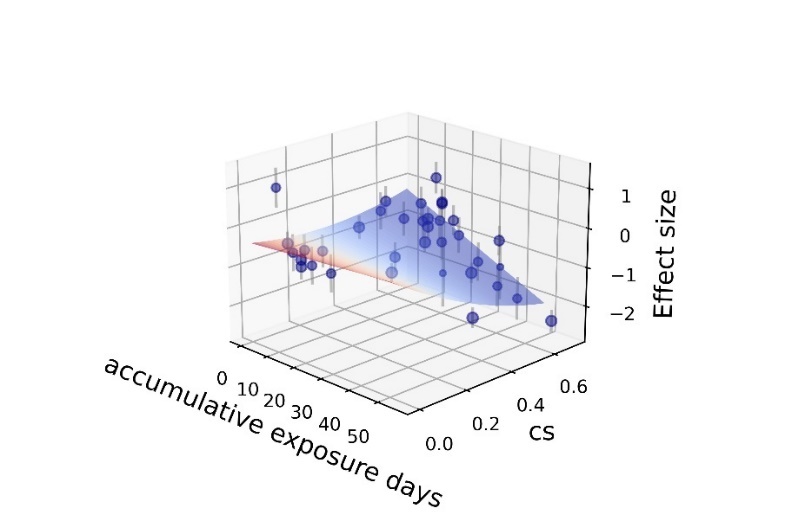 |
| --- | --- |
| 1. poly2D model fitted with accumulative exposure minutes z=0.0157-0.00064x -2.5745y+0.0000002922x²+3.0981y² -0.000821xy (R²=26.9%) | 1. poly2D model fitted with accumulative exposure days   z=-0.373+0.00264x-1.3167y+0.0000597x²+2.213y²-0.0614xy (R²=49.2%) |
| 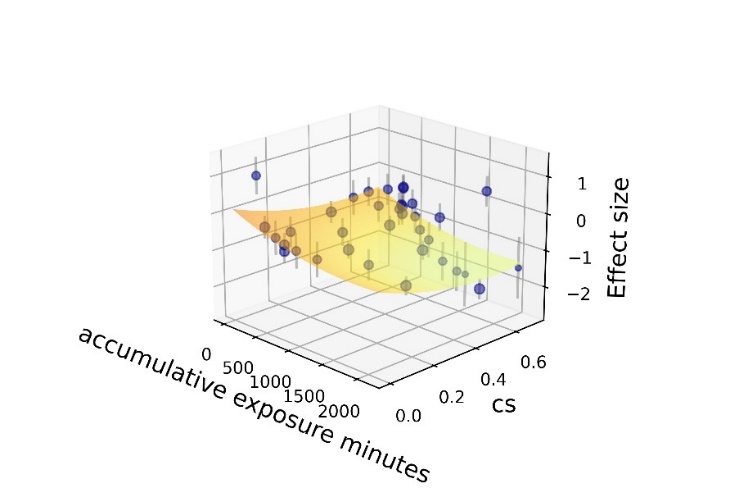 | 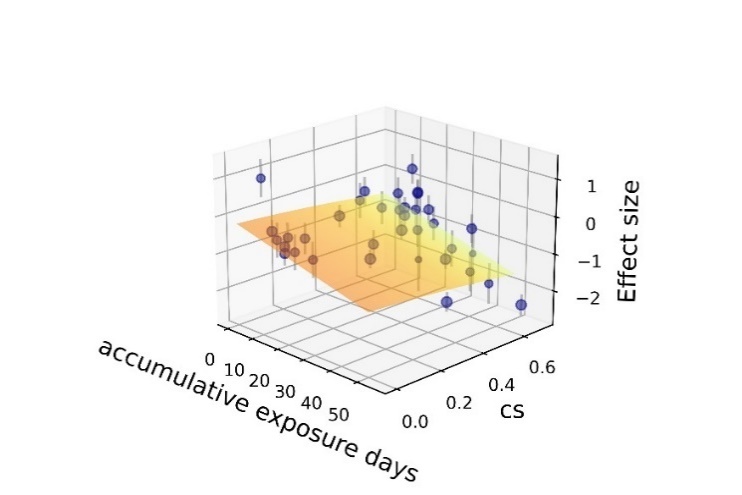 |
| 1. parabola2D model fitted with accumulative exposure minutes z=0.131-0.00089x-2.552y+0.000000241x^2^+2.3359y^2^(R²=25.8%) | 1. parabola2D model fitted with accumulative exposure days   z=-0.179-0.0193x-03812y-0.00000348x²-0.39y² (R²=40.4%) |
| 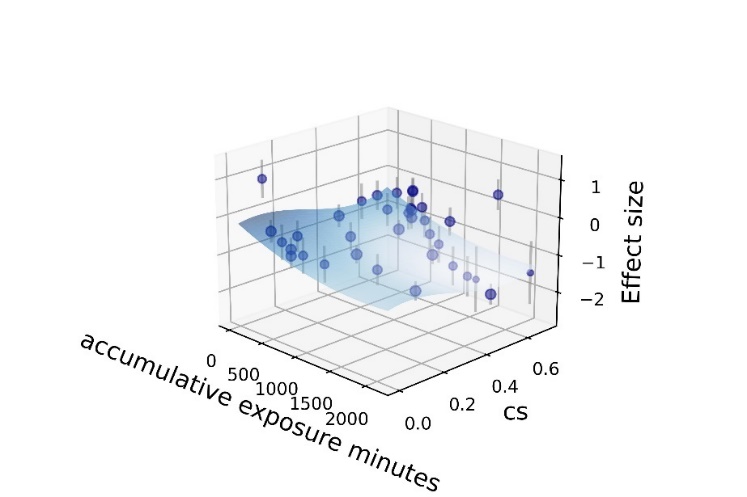 | 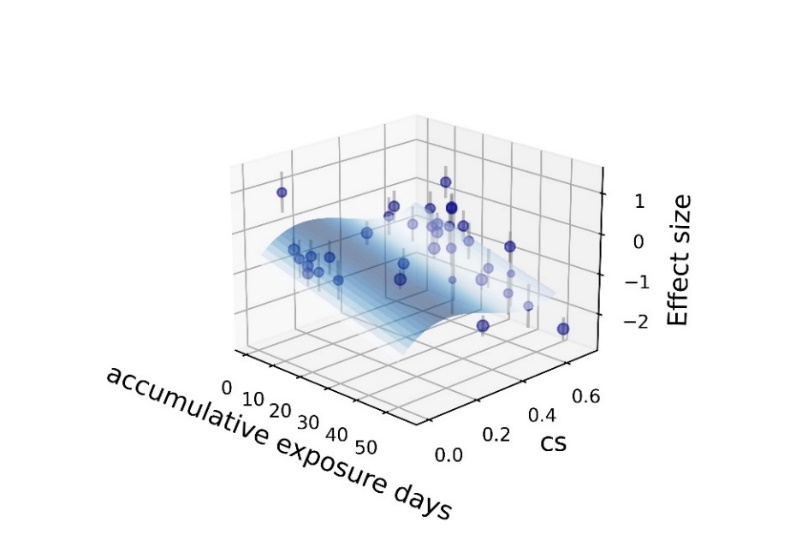 |
| 1. polynormal 2D model fitted with accumulative exposure minutes z=-0.1548-0.00095x+0.000975x²+1.55186y+0.0000002573x²-10.5235y²+11.1668y³ (R²=25.3%) | polynormal 2D model fitted with accumulative exposure days   1. z=-0.689-0.0238x+0.002x²+6.965y+0.000001612x²-23.238y²+19.7558y³ (R²=41.1%) |
| 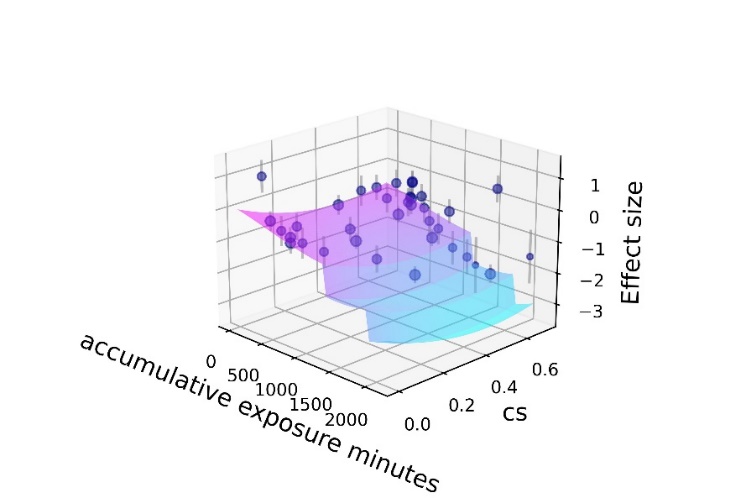 | 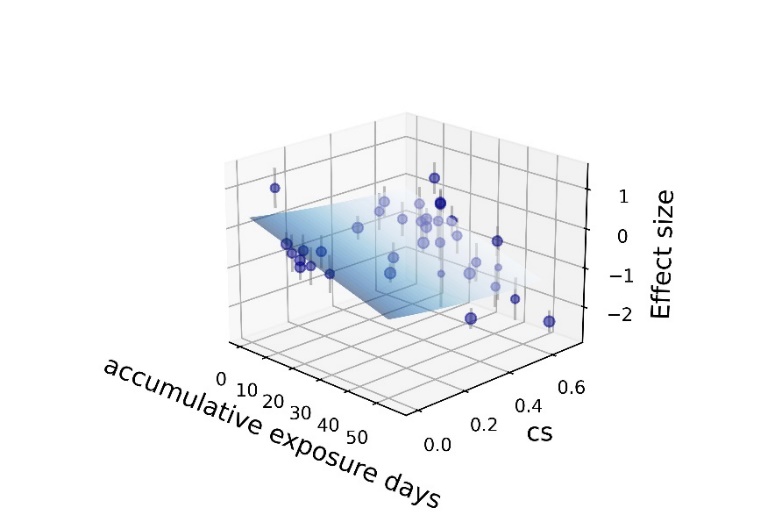 |
| 1. polynormal 2D model fitted with accumulative exposure minutes (-) | 1. polynormal 2D model fitted with accumulative exposure days z=0.286-0.095x+0.0032x²-3.68x³+1.083y+0.599y² (R²=41.3%) |

**Supplementary Figure 6 (a).** Fitting for clusters of data points of 7 studies of non-depressed unmedicated people (20 items) on various models

| 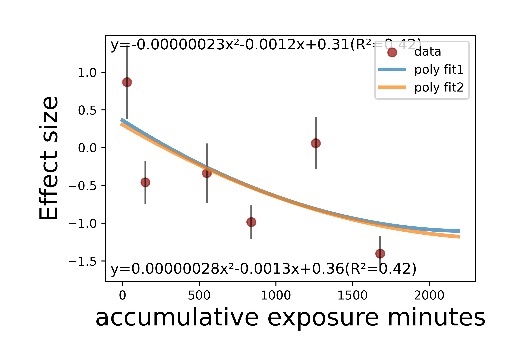 | 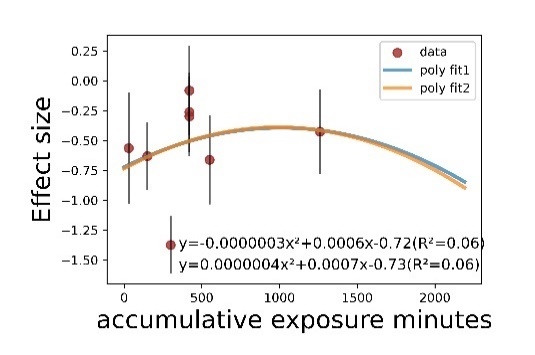 | 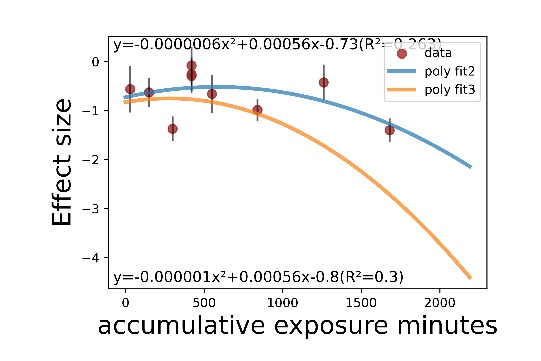 |
| --- | --- | --- |
| CS_t,f_ range ＜0.1-0.4, NA | CS_t,f_ range 0.4-0.7, NA | CS_t,f_ range 0.2-0.7, NA |
| 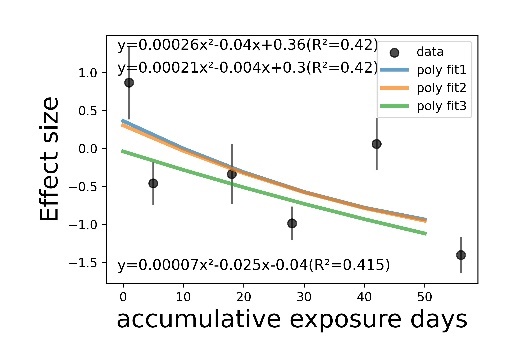 | 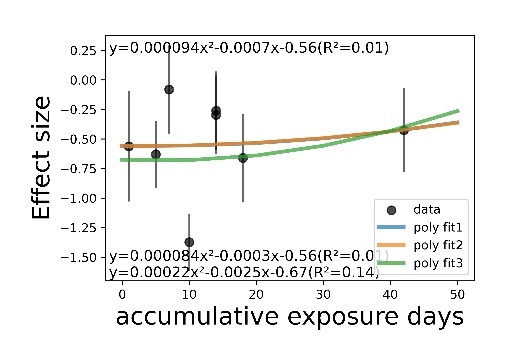 | 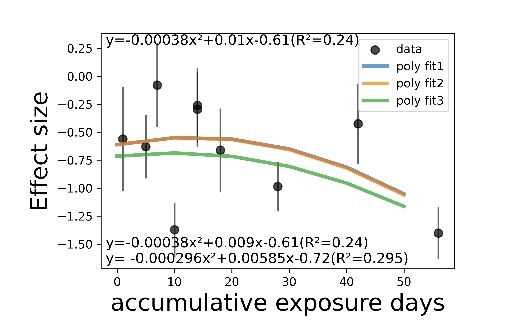 |
| CS_t,f_ range ＜0.1-0.4, NA | CS_t,f_ range 0.4-0.7, NA | CS_t,f_ range 0.2-0.7, NA |

**Supplementary Figure 6 (b).** Temporal fitting (cumulative minutes and days) and saturation among non-depressed studies within various CS_t,f_ ranges

| 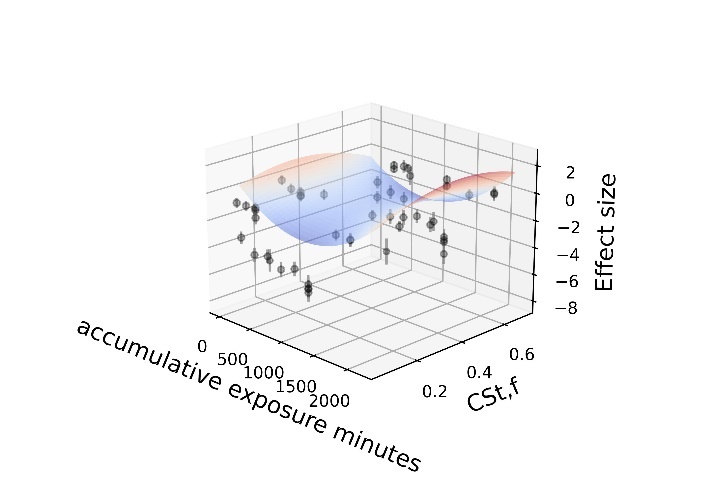 | 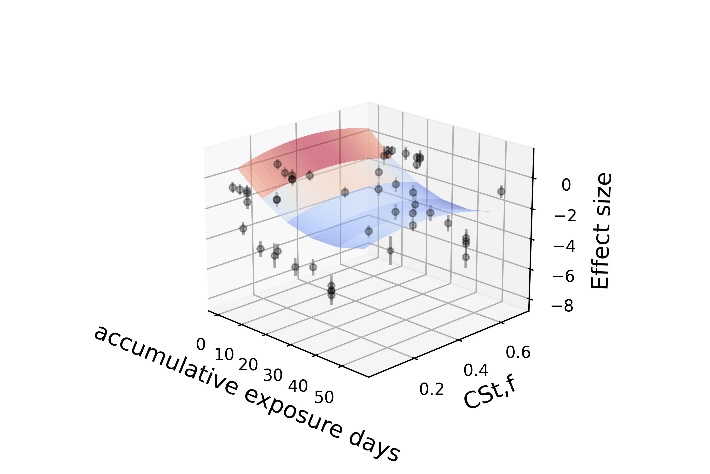 |
| --- | --- |
| poly2D model fitted with accumulative exposure minutes z=-0.408-0.0048x +8.51y+0.00000233x²-12.6y²+0.0011xy (R²=7.5%)  (888.0216224581798, 0.7) | 1. poly2D model fitted with accumulative exposure days   z=-0.214+0.167x+7.07y+0.0024x²-8.75y²+-0.023xy (R²=16.9%)  (37.515245068711785, 0.7) |
| 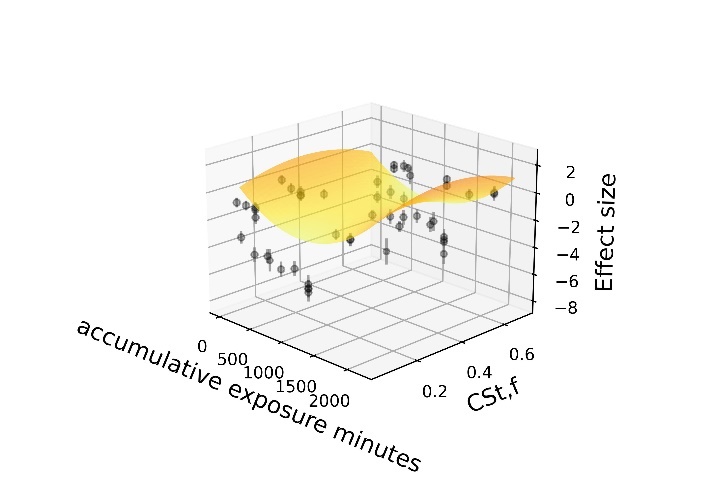 | 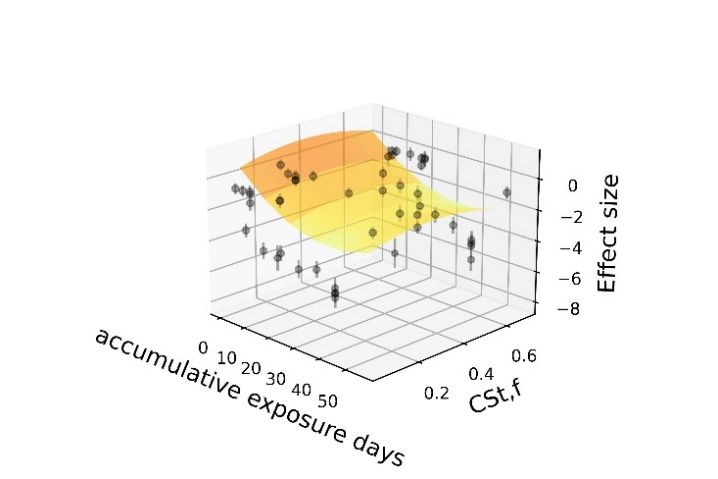 |
| parabola2D model fitted with accumulative exposure minutes z=-0.667-0.0046x+9.43y-0.0000024x2-12.63y2(R²=6.6%)  (842.8098179376984，0.7) | 1. parabola2D model fitted with accumulative exposure days   z=-0.071-0.173x+6.38y+0.00241x²-8.44y² (R²=16.7%)  (35.821033575918456, 0.7) |
| 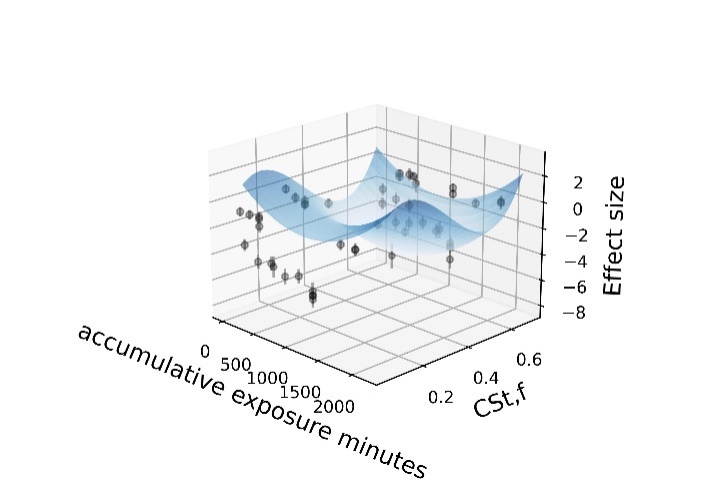 | 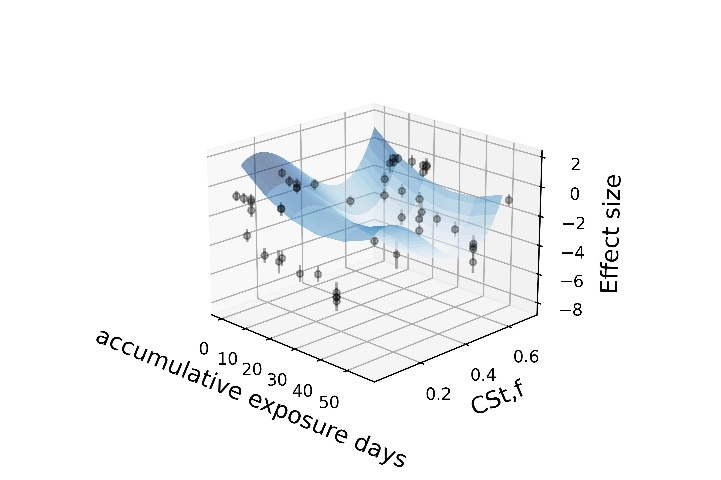 |
| polynormal 2D model fitted with accumulative exposure minutes z=-2.43-0.0048x+0.0000025x²+53.015y-201.2y²+190.4y³ (R²=18.9%)  (974.5061601272515, 0.699) | polynormal 2D model fitted with accumulative exposure days  z=-1.83-0.182x+46.8y+0.0028x²-181.6y²+174.1 y³ (R²=21.6%)  (32.275537140149346, 0.699) |
| 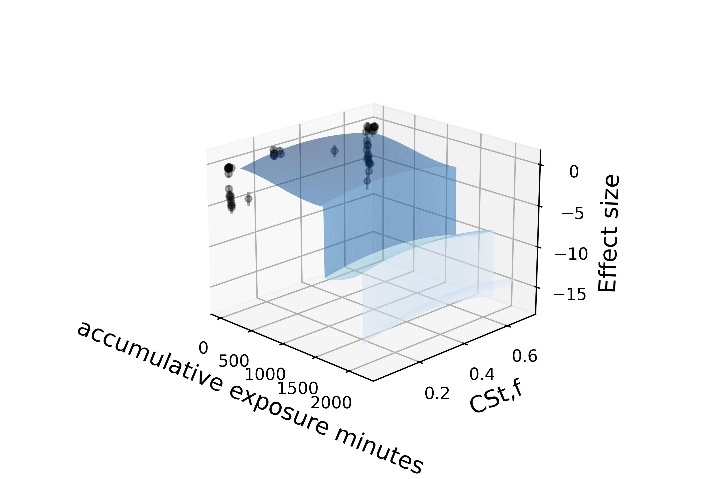 | 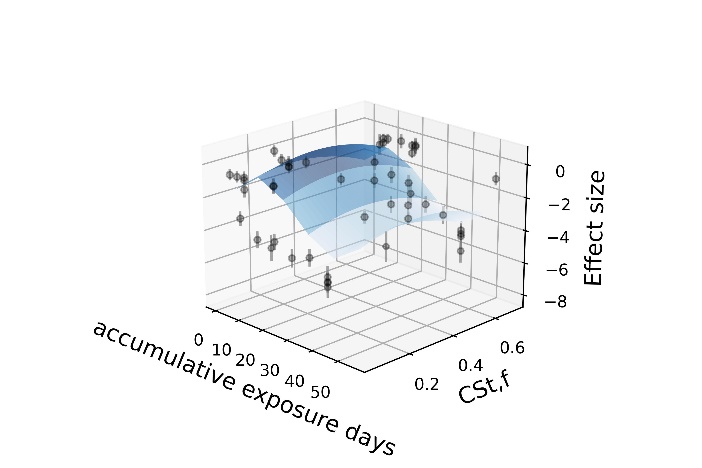 |
| polynormal 2D model fitted with accumulative exposure minutes (-) | polynormal 2D model fitted with accumulative exposure days z=-2.36+0.272x-10.43y²+7.22y+0.00023x³ (R²=31.7%) |

**Supplementary Figure 7 (a).** Fitting for clusters of 13 studies’ data points among moderate to severe depressed, medicated patients (50 items) on various models

| 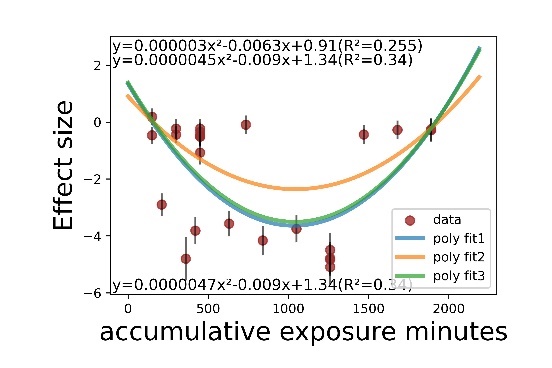 | 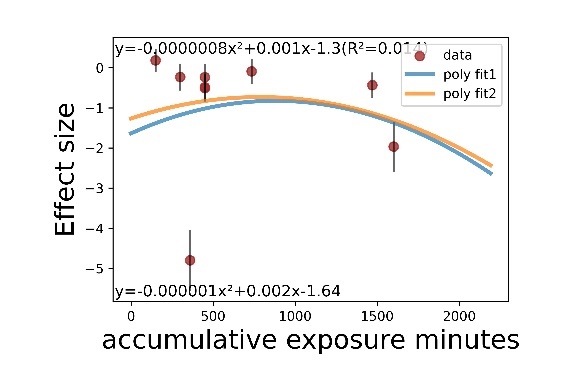 | 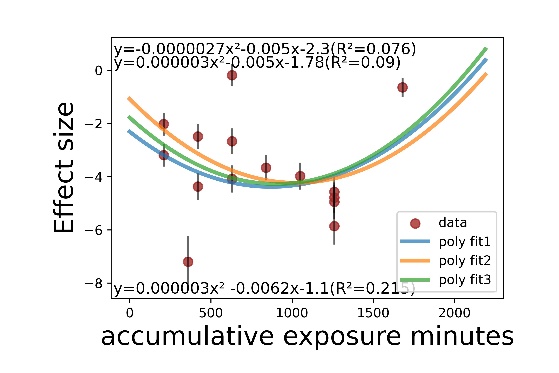 |
| --- | --- | --- |
| CS_t,f_ range ＜0.1-0.4, 1000mins | CS_t,f_ range 0.1-0.6, NA | CS_t,f_ range 0.6-0.665, 870-1000mins |
| 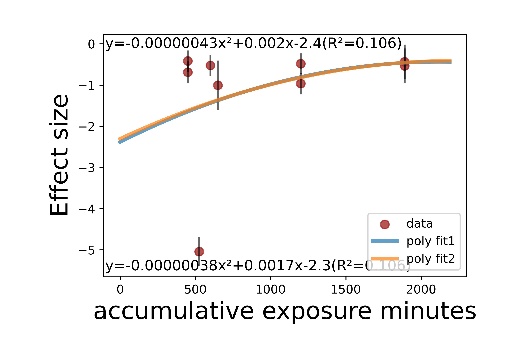 | 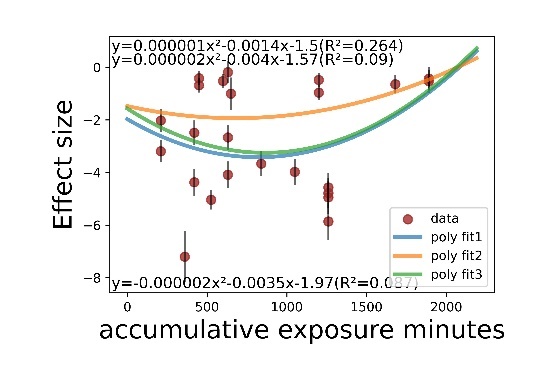 | 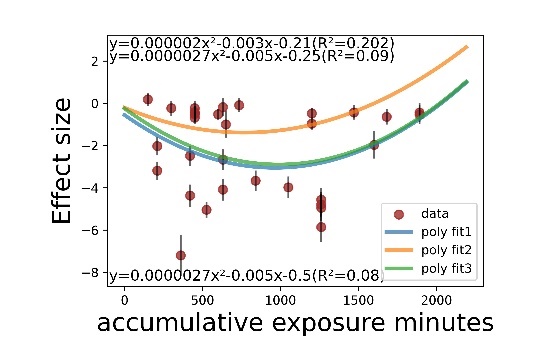 |
| CS_t,f_ range 0.665-0.7, NA | CS_t,f_ range 0.6-0.7, 680-860mins | CS_t,f_ range 0.2-0.7, 770-990mins |
| 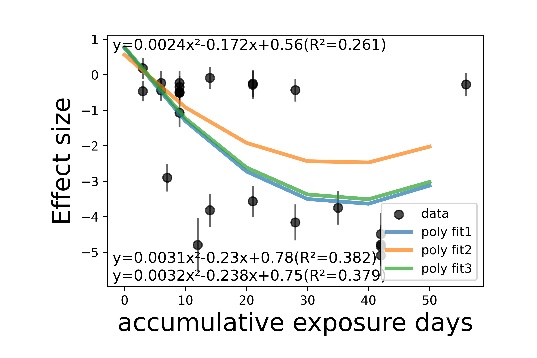 | 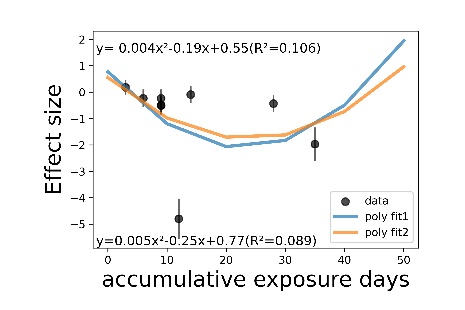 | 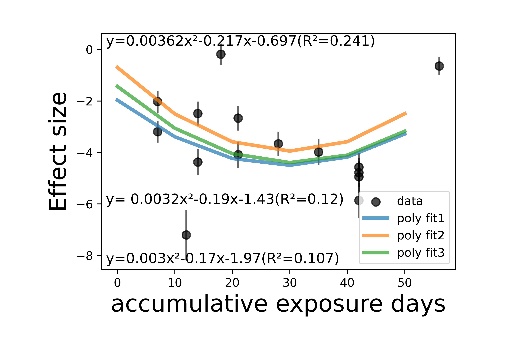 |
| CS_t,f_ range ＜0.1-0.4，36-37 days | CS_t,f_ range 0.1-0.6, 23-24 days | CS_t,f_ range 0.6-0.665, 30 days |
| 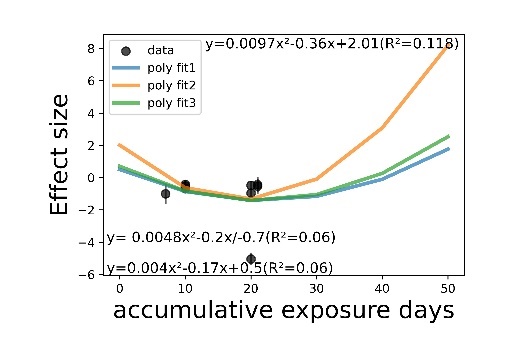 | 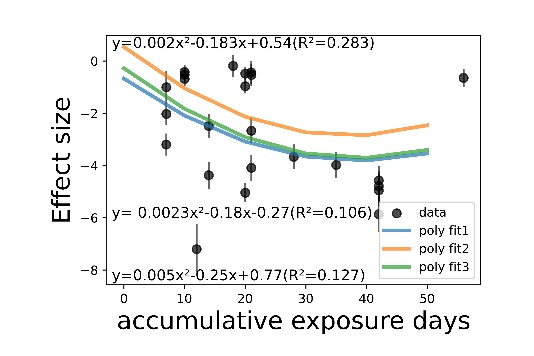 | 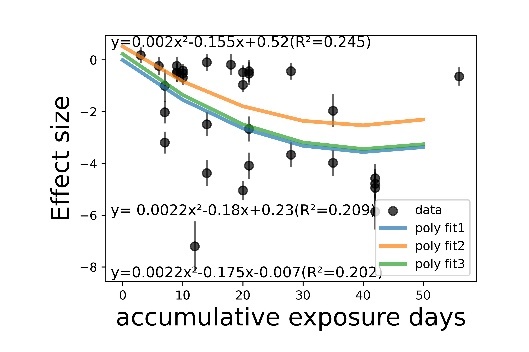 |
| CS_t,f_ range 0.665-0.7, 18-22days | CS_t,f_ range 0.6-0.7, 37-39 days | CS_t,f_ range 0.2-0.7, 40-41 days |

**Supplementary Figure 7 (b).** Temporal fitting (cumulative minutes and days) and saturation among moderate to severe depressed, co-medicated people within various CS_t,f_ ranges

| 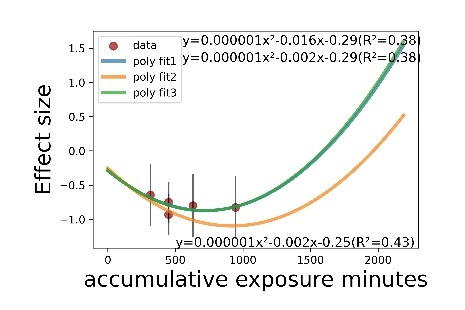 | 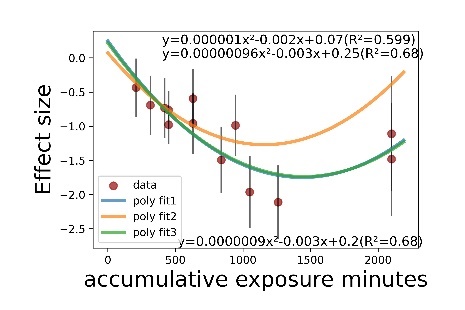 | 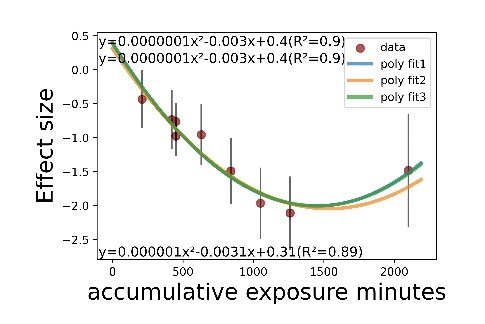 |
| --- | --- | --- |
| CS_t,f_ range ＜0.1-0.4, 720-920 mins | CS_t,f_ range 0.4-0.7, 1150-1450mins | CS_t,f_ range 0.665-0.7, 1450-1535mins |
| 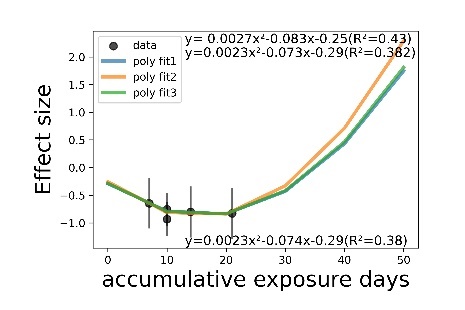 | 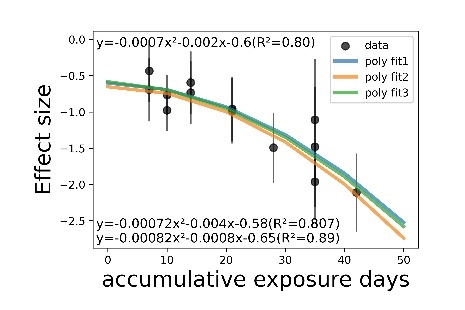 | 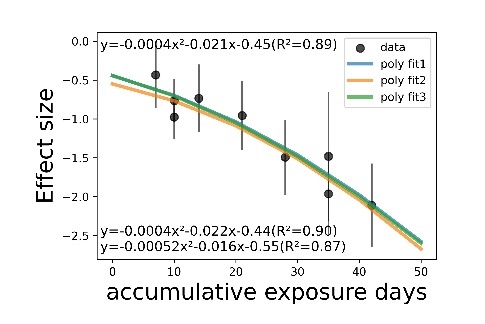 |
| CS_t,f_ range ＜0.1-0.4, 16 days | CS_t,f_ range 0.4-0.7, NA | CS_t,f_ range 0.665-0.7, NA |

**Supplementary Figure 7 (c).** Temporal fitting (cumulative minutes and days) and saturation among moderate to severe depressed, non-medicated people within various CS_t,f_ ranges

| 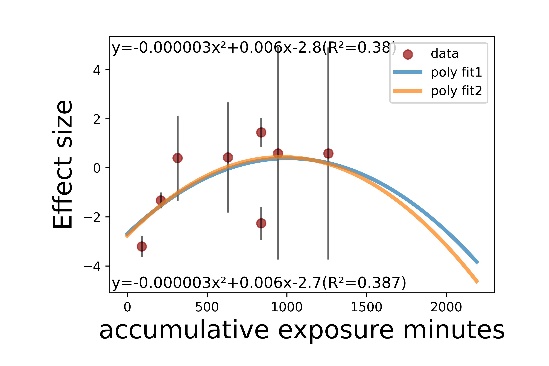 | 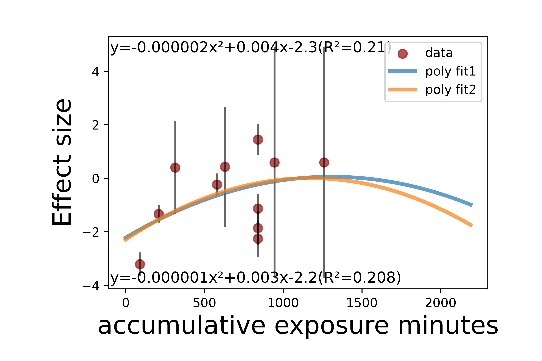 | 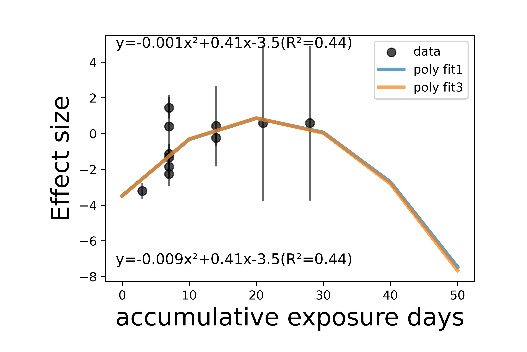 |
| --- | --- | --- |
| CS_t,f_ range 0.6-0.7, NA | CS_t,f_ range 0.2-0.7, NA | CS_t,f_ range 0.2-0.7, NA |

**Supplementary Figure 8 (a).** Temporal fitting (cumulative minutes and days) and saturation among mild to moderate depressed, co-medicated people within various CS_t,f_ ranges

| 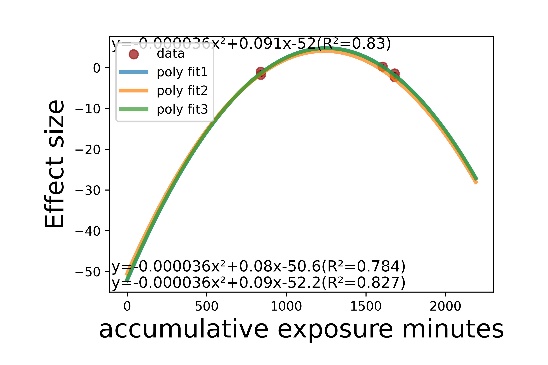 | 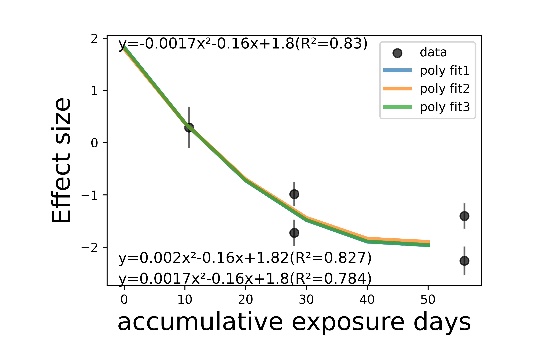 |  |
| --- | --- | --- |
| CS_t,f_ range 0.2-0.7, NA | CS_t,f_ range 0.2-0.7, 47 days |  |

**Supplementary Figure 8 (b).** Temporal fitting (cumulative minutes and days) and saturation among mild to moderate depressed, non-medicated people within various CS_t,f_ ranges

| 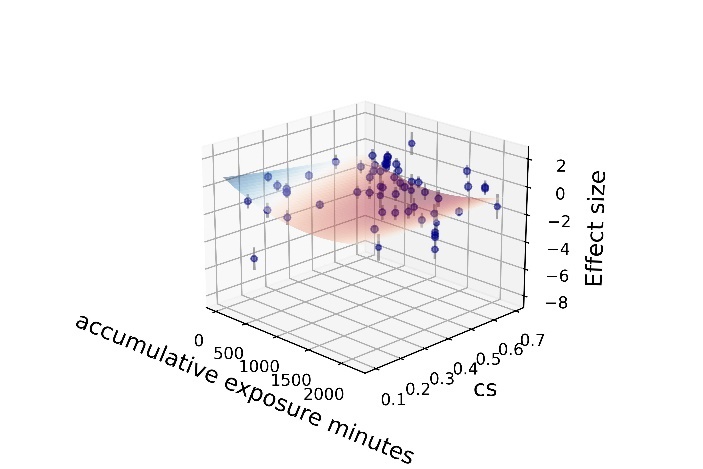 | 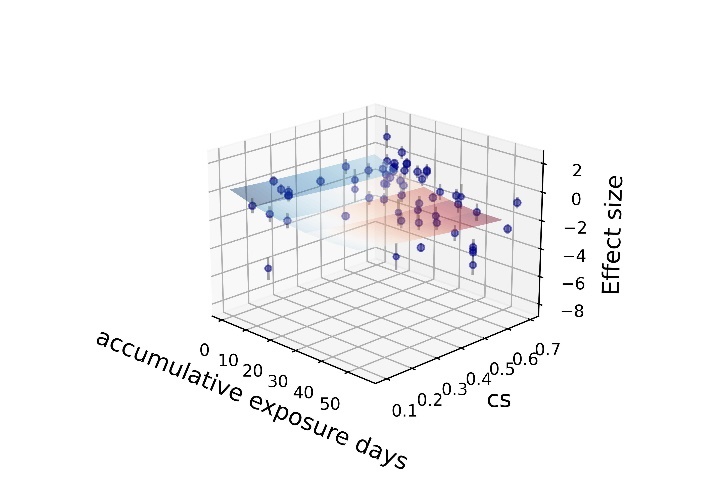 |
| --- | --- |
| 1. poly2D model fitted with accumulative exposure minutes z=1.0233-0.0028x -3.3675y+0.0000009584x²+1.16237y² -0.00107xy (R²=2.3%) | 1. poly2D model fitted with accumulative exposure days   z=0.3715-0.0634x-1.8506y+0.00088x²+1.03179y²-0.0263xy (R²=10.6%) |
| 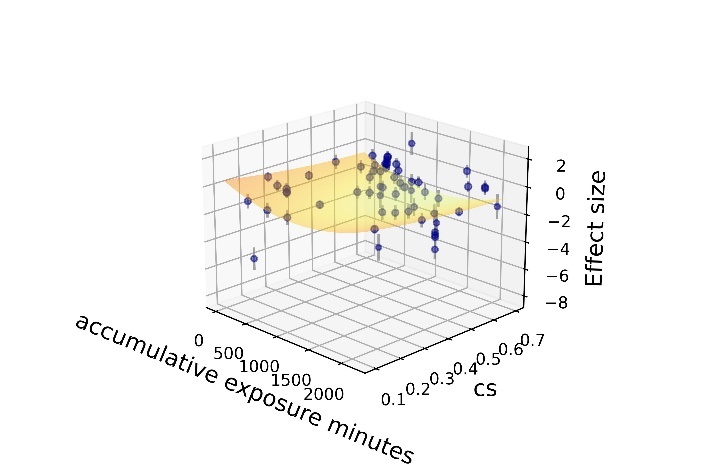 | 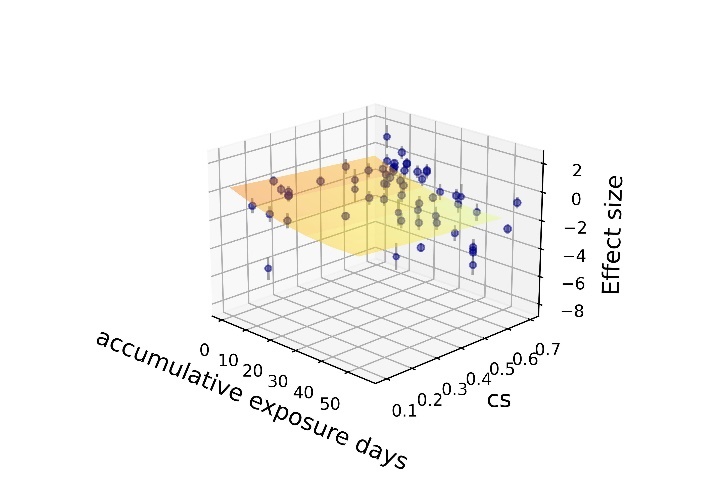 |
| 1. parabola2D model fitted with accumulative exposure minutes z=0.8443-0.00235x-3.63648y+0.00000103x^2^+2.2939y^2^(R²=2.6%) | 1. parabola2D model fitted with accumulative exposure days   z=0.499-0.076x-1.669y+0.000877x²+0.28198y² (R²=9.8%) |
| 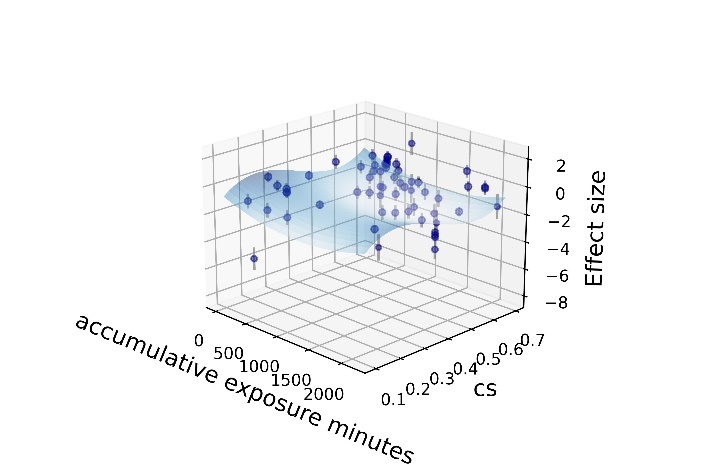 | 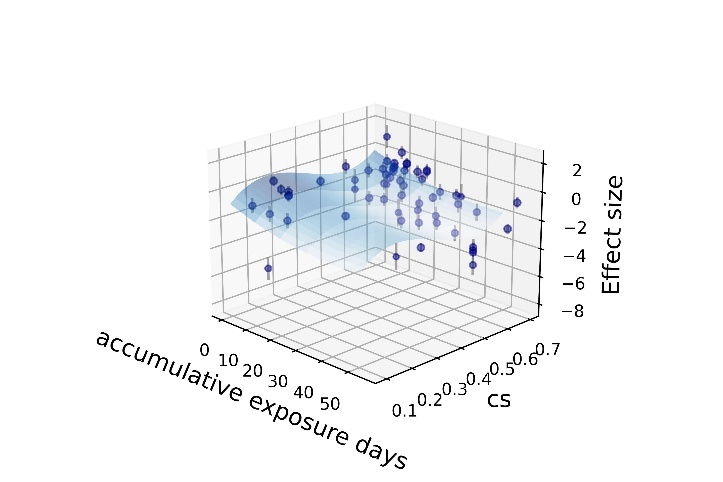 |
| 1. polynormal 2D model fitted with accumulative exposure minutes z=-3.155-0.00209x+0.000000849x²+34.0545y+0.0000002573x²-102.645y²+86.165y³ (R²=7.2%) | polynormal 2D model fitted with accumulative exposure days   1. z=-3.584-0.076x+0.002x²+37.474y+0.000857x²-108.89y²+89.7278y³ (R²=16.2%) |

**Supplementary Figure 9 (a).** Fitting for clusters of data points of circadian, random-controlled trials (22 studies, 61 items)

| 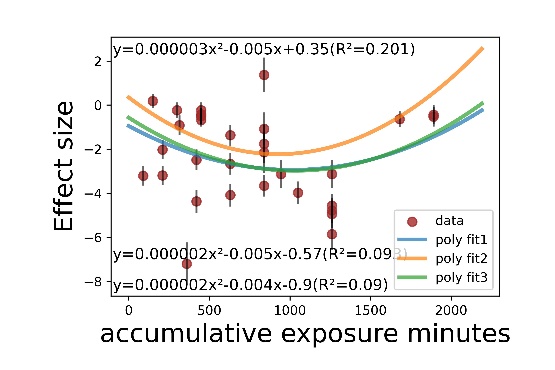 | 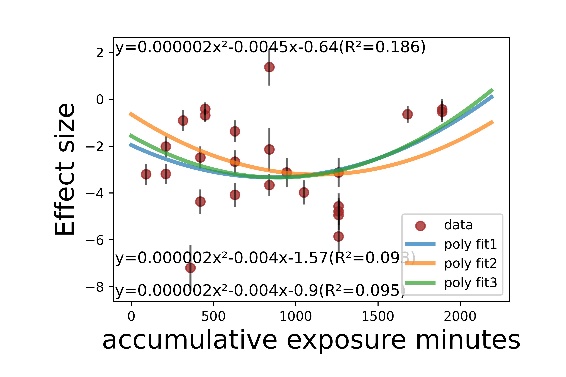 |
| --- | --- |
| CS_t,f_ range 0.2-0.7, 930-1030 mins | CS_t,f_ range 0.6-0.7, 850-1135 mins |
| 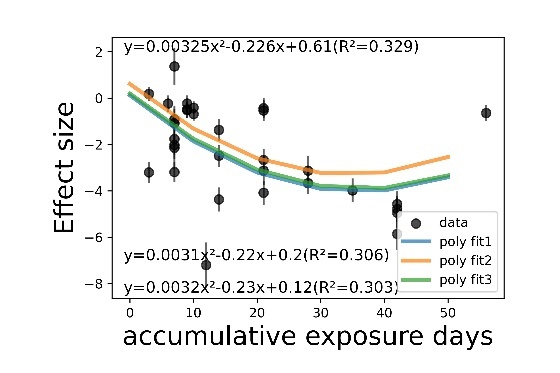 | 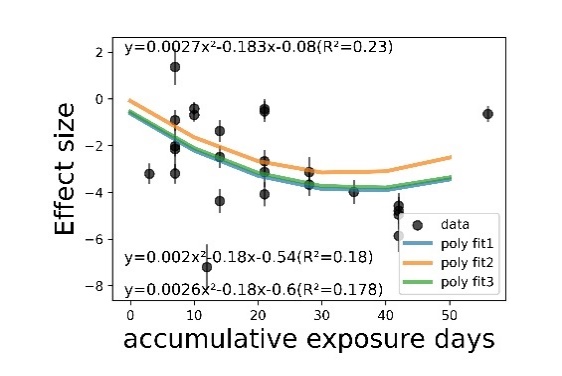 |
| CS_t,f_ range 0.2-0.7, days | CS_t,f_ range 0.6-0.7, 34-36 days |

**Supplementary Figure 9 (b).** Temporal fitting (cumulative minutes and days) and saturation of circadian, RCT trials among depressed, medicated people (studies, 33 items)

| 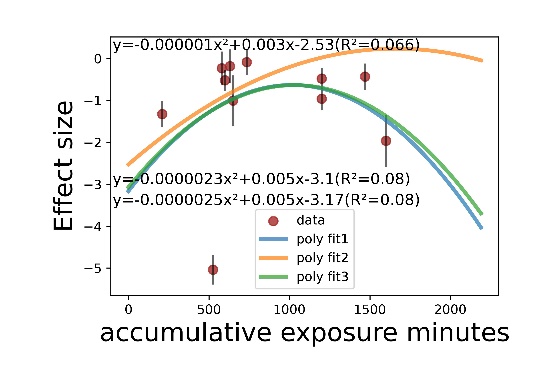 |  |
| --- | --- |
| CS_t,f_ range 0.2-0.7, NA | CS_t,f_ range 0.6-0.7, NA |
|  |  |
| CS_t,f_ range 0.2-0.7, NA | CS_t,f_ range 0.6-0.7, 38-39 days |

**Supplementary Figure 10.** Temporal fitting (cumulative minutes and days) and saturation of circadian, quasi-experimental trials among depressed, medicated people (8 studies, 11 items)

|  |  |
| --- | --- |
| (a) | (b) |
|  |  |
| (c) | (d) |
|  |  |
| (e) |  |

**Supplementary Figure 11.** Funnel Plot and Egger’s test among: (a) 31 articles with all outcomes (97 items); (b) 31 articles with only “circadian” results (73 items); (c) only 24 depression-related articles (85 items); (d) only 18 co-medication studies (61 items); (e) only 22 RCT studies (85 items)

# References

1. Zhou L, Hou D, Wang Y, Zhou S, Lin Y. High circadian stimulus lighting therapy for depression: Meta-analysis of clinical trials. *Front. Neurosci*. (2022) 16. doi:10.3389/fnins.2022.975576.
2. Rea MS. A guide for characterizing and prescribing light therapy devices. Ed. In Auger, *Circadian rhythm sleep-wake disorders: An evidence-based guide for clinicians and investigators.* Springer, Cham (2020) p. 207–219. doi:10.1007/978-3-030-43803-6 14.
3. Do A, Li VW, Huang S, Michalak EE, Tam EM, Chakrabarty T, et al. Blue-Light Therapy for Seasonal and Non-Seasonal Depression: A Systematic Review and Meta-Analysis of Randomized Controlled Trials. *Can J Psychiatry*. (2022) 67(10):745–754. doi:10.1177/07067437221097903.
4. Geoffroy PA, Schroder CM, Reynaud E, Bourgin P. Efficacy of light therapy versus antidepressant drugs, and of the combination versus monotherapy, in major depressive episodes: A systematic review and meta-analysis. *Sleep Med Rev*. (2019) 48:101213. doi:10.1016/j.smrv.2019.101213.
5. Nguyen C, Murray G, Anderson S, Filipowicz A, Ingram KK. In vivo molecular chronotyping, circadian misalignment, and high rates of depression in young adults. *J Affect Disord*. (2019) 250:425–431. doi:10.1016/j.jad.2019.03.050.
6. Haraden DA, Mullin BC, Hankin BL. Internalizing symptoms and chronotype in youth: a longitudinal assessment of anxiety, depression and tripartite model. *Psychiat Res*. (2019) 272:797–805. doi:10.1016/j.psychres.2018.12.117.
7. Faria AD, de Azevedo Cardoso T, Mondin TC, de Mattos Souza LD, da Silva Magalhaes PV, Zeni CP, et al. Biological rhythms in bipolar and depressive disorders: a community study with drug-na ̈ıve young adults. *J Affect Disord*. (2015) 186:145–148. doi:10.1016/j.jad.2015.07.004.
8. Mondin TC, de Azevedo Cardoso T, de Mattos Souza LD, Jansen K, da Silva Magalh ̃aes PV, Kapczinski F, et al. Mood disorders and biological rhythms in young adults: a large population-based study. *J Psychiatr Res*. (2017) 84:98–104. doi:10.1016/j.jpsychires.2016.09.030.
9. Robillard R, Carpenter JS, Rogers NL, Fares S, Grierson AB, Hermens DF, et al. Circadian rhythms and psychiatric profiles in young adults with unipolar depressive disorders. *Ttansl Psychiat.* (2018) 8(1):213. doi:10.1038/s41398-018-0255-y.
10. Sivertsen B, Harvey AG, Pallesen S, Hysing M. Mental health problems in adolescents with delayed sleep phase: results from a large population-based study in Norway. *J Sleep Res*. (2015) 24(1):11–18. doi:10.1111/jsr.12254.
11. Crouse JJ, Carpenter JS, Song YJC, Hockey SJ, Naismith SL, Grunstein RR, et al. Circadian rhythm sleep–wake disturbances and depression in young people: implications for prevention and early intervention. *Lancet Psychiatry*. (2021) 8(9):813–823. doi:10.1016/s2215-0366(21)00034-1.
12. Burish MJ, Chen Z, Yoo SH. Emerging relevance of circadian rhythms in headaches and neuropathic pain. *Acta Physiologica*. (2019) 225(1):e13161. doi:10.1111/apha.13161.
13. Parry BL. Optimal management of perimenopausal depression. *Int J Womens Health.* (2010) p. 143–151. doi:10.2147/ijwh.s7155.
14. Lam RW, Levitan RD. Pathophysiology of seasonal affective disorder: a review. *J Psychiatry Neurosci.* (2000) 25(5):469.
15. Meyerhoff J, Young MA, Rohan KJ. Patterns of depressive symptom remission during the treatment of seasonal affective disorder with cognitive-behavioral therapy or light therapy. *Depress Anxiety*. (2018) 35(5):457–467. doi:10.1002/da.22739.
16. Al-Karawi D, Jubair L. Bright light therapy for nonseasonal depression: meta-analysis of clinical trials. *J Affect Disord*. (2016) 198:64–71. doi:10.1016/j.jad.2016.03.016.
17. Wang S, Zhang Z, Yao L, Ding N, Jiang L, Wu Y. Bright light therapy in the treatment of patients with bipolar disorder: A systematic review and meta-analysis. *PLoS One*. (2020) 15(5):e0232798. doi:10.1371/journal.pone.0232798.
18. Lam RW, Levitt AJ, Levitan RD, Michalak EE, Cheung AH, Morehouse R, et al. Efficacy of bright light treatment, fluoxetine, and the combination in patients with nonseasonal major depressive disorder: a randomized clinical trial. *JAMA psychiatry*. (2016) 73(1):56–63. doi:10.1001/jamapsychiatry.2015.2235.
19. Niederhofer H. Stabilization of circadian rhythm, its augmentation by bright light treatment and its importance for ADHD and depression of adolescents. *Neurosci Med.* (2013) 4(03):150–154. doi:10.4236/nm.2013.43024.
20. Lin F, Su Y, Weng Y, Lin X, Weng H, Cai G, et al. The effects of bright light therapy on depression and sleep disturbances in patients with Parkinson’s disease: a systematic review and meta-analysis of randomized controlled trials. *Sleep Med.* (2021) 83:280–289. doi:10.1016/j.sleep.2021.03.035.
21. Roccaro I, Smirni D. Fiat lux: The light became therapy. An overview on the bright light therapy in Alzheimer’s disease sleep disorders. *J Alzheimer’s Disease.* (2020) 77(1):113–125. doi:10.3233/jad-200478.
22. Epperson CN, Terman M, Terman JS, Hanusa BH, Oren DA, Peindl KS, et al. Randomized clinical trial of bright light therapy for antepartum depression: preliminary findings. *J Clin Psychiatry.* (2004) 65(3):421–425. doi:10.4088/jcp.v65n0319.
23. Auger RR, Burgess HJ, Emens JS, Deriy LV, Thomas SM, Sharkey KM. Clinical practice guideline for the treatment of intrinsic circadian rhythm sleep-wake disorders: advanced sleep-wake phase disorder (ASWPD), delayed sleep-wake phase disorder (DSWPD), non-24-hour sleep-wake rhythm disorder (N24SWD), and irregular sleep-wake rhythm disorder (ISWRD). An update for (2015: an American Academy of Sleep Medicine clinical practice guideline. *J Clin Sleep Med.* (2015) 11(10):1199–1236. doi:10.5664/jcsm.5100.
24. Hurd D, Herrera M, Brant JM, Coombs NC, Arzubi E. Prospective, open trial of adjunctive triple chronotherapy for the acute treatment of depression in adolescent inpatients. *J Child Adolesc Psychopharmacol.* (2019) (29(1):20–27. doi:10.1089/cap.2018.0063.
25. Even C, Schr ̈oder CM, Friedman S, Rouillon F. Efficacy of light therapy in nonseasonal depression: a systematic review. *J Affect Disord*. (2008) 108(1-2):11–23. doi:10.1016/j.jad.2007.09.008.
26. Terman M, Terman JS. Controlled trial of naturalistic dawn simulation and negative air ionization for seasonal affective disorder. *Am J Psychiatry.* (2006) 163(12):2126–2133. doi:10.1176/ajp.2006.163.12.2126.
27. Alotaibi MA, Halaki M, Chow CM. A systematic review of light therapy on mood scores in major depressive disorder: light specification, dose, timing and delivery. *Int J Basic Appl Sci*. (2016) 5(1):30–37. doi: 10.14419/ijbas.v5i1.5456.
28. Geoffroy PA, Maruani J, Etain B, Lejoyeux M, Amad A, Courtet P, et al. Bright light therapy in the morning or at mid-day in the treatment of non-seasonal bipolar depressive episodes (LuBi): study protocol for a dose research phase I/II trial. *Psychiatry Investig.* (2018) 15(12):1188. doi:10.30773/pi.2018.09.27.1.
29. Janas-Kozik M, Krzystanek M, Stachowicz M, Krupka-Matuszczyk I, Janas A, Rybakowski JK. Bright light treatment of depressive symptoms in patients with restrictive type of anorexia nervosa. *J Affect Disord*. (2011) 130(3):462–465. doi:10.1016/j.jad.2010.10.014.
30. Donmez M, Yorguner N, Kora K, Topcuoglu V. Efficacy of bright light therapy in perinatal depression: A randomized, double-blind, placebo-controlled study. *J Psychiatr Res.* (2022) 149:315–322. doi:10.1016/j.jpsychires.2022.02.027.
31. Jiang L, Zhang S, Wang Y, So KF, Ren C, Tao Q. Efficacy of light therapy for a college student sample with non-seasonal subthreshold depression: an RCT study. *J Affect Disord*. (2020) (277:443–449. doi:10.1016/j.jad.2020.08.055.
32. Rea MS, Nagare R, Figueiro MG. Predictions of melatonin suppression during the early biological night and their implications for residential light exposures prior to sleeping. *Sci Rep.* (2020) 10(1):14114. doi:10.1038/s41598-020-70619-5.
33. Daneault V, Dumont M, Masse E, Vandewalle G, Carrier J. Light-sensitive brain pathways and aging. *J Physiol Anthropol.* (2016) 35(1):1–12. doi:10.1186/s40101-016-0091-9.
34. Stephenson KM, Schroder CM, Bertschy G, Bourgin P. Complex interaction of circadian and non-circadian effects of light on mood: shedding new light on an old story. *Sleep Med Rev.* (2012) 16(5):445–454. doi:10.1016/j.smrv.2011.09.002.
35. Boivin DB, Czeisler CA, Dijk DJ, Duffy JF, Folkard S, Minors DS, et al. Complex interaction of the sleep-wake cycle and circadian phase modulates mood in healthy subjects. *Arch Gen Psychiatry.* 1997) 54(2):145–152. doi:10.1001/archpsyc.1997.01830140055010.
36. Oldham MA, Ciraulo DA. Bright light therapy for depression: a review of its effects on chronobiology and the autonomic nervous system. *Chronobiol Int.* (2014) 31(3):305–319. doi:10.3109/07420528.2013.833935.
37. Lucas RJ, Peirson SN, Berson DM, Brown TM, Cooper HM, Czeisler CA, et al. Measuring and using light in the melanopsin age. *Trends Neurosci.* (2014) 37(1):1–9. doi:10.1016/j.tins.2013.10.004.
38. Cie S. 026/E: 2018 CIE System for Metrology of Optical Radiation for ipRGC-Influenced Responses to Light. *Color Res Appl*. (2018) 44:316–316.
39. Brown TM, Thapan K, Arendt J, Revell VL, Skene DJ. S-cone contribution to the acute melatonin suppression response in humans. *J Pineal Res.* (2021) 71(1):e12719. doi:10.1111/jpi.12719.
40. CIE T. *Fundamental chromaticity diagram with physiological axes*. In CIE Technical Report (2006).
41. Anderson JL, Glod CA, Dai J, Cao Y, Lockley SW. Lux vs. wavelength in light treatment of Seasonal Affective Disorder. *Acta Psychiatr Scand.* (2009) 120(3):203–212. doi:10.1111/j.1600-0447.2009.01345.x.
42. Rea MS, Nagare R, Figueiro MG. Modeling circadian phototransduction: Quantitative predictions of psychophysical data. *Front Neurosci.* (2021) 15:615322. doi:10.3389/fnins.2021.615322.
43. Rea MS, Nagare R, Figueiro MG. Modeling circadian phototransduction: retinal neurophysiology and neuroanatomy. *Front Neurosci.* (2021) 14:615305. doi:10.3389/fnins.2020.615305.
44. Tsai JW, Hannibal J, Hagiwara G, Colas D, Ruppert E, Ruby NF, et al. Melanopsin as a sleep modulator: circadian gating of the direct effects of light on sleep and altered sleep homeostasis in Opn4-/- mice. *PLoS Biol*. (2009) 7(6):e1000125. doi:10.1371/journal.pbio.1000125.
45. Maruani J, Geoffroy PA. Multi-Level processes and retina–brain pathways of photic regulation of mood. *J Clin Med.* (2022) 11(2):448. doi:10.3390/jcm11020448.
46. Chen Y, Chen T, Cai X. Light-sensitive circuits related to emotional processing underlie the antidepressant neural targets of light therapy. *Behav Brain Res.* (2021) 396:112862. doi:10.1016/j.bbr.2020.112862.
47. Dong Y, Hu H. Taming the “Black Dog” by light: a retina-habenula circuit mechanism unveiled. *Neuron.* (2019) 102(1):3–5. doi:10.1016/j.neuron.2019.02.033.
48. Huang L, Xi Y, Peng Y, Yang Y, Huang X, Fu Y, et al. A visual circuit related to habenula underlies the antidepressive effects of light therapy. *Neuron.* (2019) 102(1):128–142. doi:10.1016/j.neuron.2019.01.037.
49. Fiori LM, Kos A, Lin R, Th ́eroux JF, Lopez JP, K ̈uhne C, et al. miR-323a regulates ERBB4 and is involved in depression. *Mol Psychiatry.* (2021) (26(8):4191–4204. doi:10.1038/s41380-020-00953-7.
50. Kennard BD, Silva SG, Tonev S, Rohde P, Hughes JL, Vitiello B, et al. Remission and recovery in the Treatment for Adolescents with Depression Study (TADS): acute and long-term outcomes. *J Am Acad Child Adolesc Psychiatry.* (2009) 48(2):186–195. doi:10.1097/chi.0b013e31819176f9.
51. Mayer JS, Hees K, Medda J, Grimm O, Asherson P, Bellina M, et al. Bright light therapy versus physical exercise to prevent co-morbid depression and obesity in adolescents and young adults with attention-deficit/hyperactivity disorder: study protocol for a randomized controlled trial. *Trials.* (2018) 19(1):1–19. doi:10.1186/s13063-017-2426-1.
52. Curry J, Silva S, Rohde P, Ginsburg G, Kratochvil C, Simons A, et al. Recovery and recurrence following treatment for adolescent major depression. *Arch Gen Psychiatry.* (2011) 68(3):263–269. doi:10.1001/archgenpsychiatry.2010.150.
53. Robillard R, Hermens DF, Naismith SL, White D, Rogers NL, Ip TK, et al. Ambulatory sleep-wake patterns and variability in young people with emerging mental disorders. *J Psychiatry Neurosci.* (2015) 40(1):28–37. doi:10.1503/jpn.130247.
54. Ballard R, Parkhurst J, Julian K, Pasetes LN, Fawcett A, Li A, et al. Light Therapy for Adolescent Depression: A Scoping Review. *Curr Psychiatry Rep.* (2023) p. 1–14. doi:10.1007/s11920-023-01437-5.
55. Norren DV, Vos JJ. Spectral transmission of the human ocular media. *Vision Res*. (1974) 14(11):1237–1244. doi:10.1016/0042-6989(74)90222-3.
56. Nagare R, Rea MS, Plitnick B, Figueiro MG. Nocturnal melatonin suppression by adolescents and adults for different levels, spectra, and durations of light exposure. *J Biol Rhythms.* (2019) 34(2):178–194. doi:10.1177/0748730419828056.
57. Eto T, Ohashi M, Nagata K, Shin N, Motomura Y, Higuchi S. Crystalline lens transmittance spectra and pupil sizes as factors affecting light-induced melatonin suppression in children and adults. *Ophthalmic Physiol Opt.* (2021) 41(4):900–910. doi:10.1111/opo.12809.
58. Gibson EM, Williams III WP, Kriegsfeld LJ. Aging in the circadian system: considerations for health, disease prevention and longevity. *Exp Gerontol.* (2009) 44(1-2):51–56. doi:10.1016/j.exger.2008.05.007.
59. Kessler BA, Stanley EM, Frederick-Duus D, Fadel J. Age-related loss of orexin/hypocretin neurons. *Neuroscience.* (2011) 178:82–88. doi:10.1016/j.neuroscience.2011.01.031.
60. Liberati A, Altman DG, Tetzlaff J, Mulrow C, Gøtzsche PC, Ioannidis JP, et al. The PRISMA statement for reporting systematic reviews and meta-analyses of studies that evaluate health care interventions: explanation and elaboration. *Ann Intern Med.* (2009) 151(4):W–65. doi:10.7326/0003-4819-151-4-200908180-00136.
61. Smarr KL, Keefer AL. Measures of depression and depressive symptoms: Beck depression Inventory-II (BDI-II), center for epidemiologic studies depression scale (CES-D), geriatric depression scale (GDS), hospital anxiety and depression scale (HADS), and patient health Questionnaire-9 (PHQ-9). *Arthritis Care Res.* (2011) 63(S11):S454–S466. doi:10.1002/acr.20556.
62. House LA, Walton B. The effectiveness of light therapy for college student depression. *J College Stud Psychother.* (2018) 32(1):42–52. doi:10.1080/87568225.2017.1321975.
63. Huang LB, Tsai MC, Chen CY, Hsu SC. The effectiveness of light/dark exposure to treat insomnia in female nurses undertaking shift work during the evening/night shift. *J Clin Sleep Med.* (2013) 9(7):641–646. doi:10.5664/jcsm.2824.
64. Kirschbaum-Lesch I, Gest S, Legenbauer T, Holtmann M. Feasibility and efficacy of bright light therapy in depressed adolescent inpatients. *Zeitschrift f ̈ur Kinder-und Jugendpsychiatrie und Psychotherapie*. (2018) doi:10.1024/1422-4917/a000603.
65. Danielsson K, Jansson-Fr ̈ojmark M, Broman JE, Markstr ̈om A. Cognitive behavioral therapy as an adjunct treatment to light therapy for delayed sleep phase disorder in young adults: a randomized controlled feasibility study. *Behav Sleep Med.* (2016) 14(2):212–232. doi:10.1080/15402002.2014.981817.
66. Gest S, Holtmann M, Bogen S, Schulz C, Pniewski B, Legenbauer T. Chronotherapeutic treatments for depression in youth. *Eur Child Adolesc Psychiatry*. (2016) 25:151–161. doi:10.1007/s00787-015-0720-6.
67. Kopp BT, Hayes Jr D, Ghera P, Patel A, Kirkby S, Kowatch RA, et al. Pilot trial of light therapy for depression in hospitalized patients with cystic fibrosis. *J Affect Disord*. (2016) 189:164–168. doi:10.1016/j.jad.2015.08.056.
68. LaRosa KN, MacArthur E, Wang F, Zhang H, Pan H, Brigden J, et al. Light Therapy for QoL/Depression in AYA With Cancer: A Randomized Trial. *J Pediatr Psychol.* (2022) 47(3):306–317. doi:10.1093/jpepsy/jsab098.
69. Ricketts EJ, Burgess HJ, Montalbano GE, Coles ME, McGuire JF, Thamrin H, et al. Morning light therapy in adults with Tourette’s disorder. *J Neurol.* (2022) p. 1–12. doi:10.1007/s00415-021-10645-z.
70. Braun DL, Sunday SR, Fornari VM, Halmi KA. Bright light therapy decreases winter binge frequency in women with bulimia nervosa: a double-blind, placebo-controlled study. *Compr Psychiatry*. (1999) 40(6):442–448. doi:10.1016/s0010-440x(99)90088-3.
71. Blouin AG, Blouin JH, Iversen H, Carter J, Goldstein C, Goldfield G, et al. Light therapy in bulimia nervosa: a double-blind, placebo-controlled study. *Psychiatry Res.* (1996) 60(1):1–9. doi:10.1016/0165-1781(95)02532-4.
72. Bromundt V, Wirz-Justice A, Kyburz S, Opwis K, Dammann G, Cajochen C. Circadian sleep-wake cycles, well-being, and light therapy in borderline personality disorder. *J Pers Disord.* (2013) 27(5):680–696. doi:10.1521/pedi201226057.
73. Bais B, Kamperman AM, Bijma HH, Hoogendijk WJ, Souman JL, Knijff E, et al. Effects of bright light therapy for depression during pregnancy: a randomised, double-blind controlled trial. *BMJ open.* (2020) 10(10):e038030. doi:10.1136/bmjopen-2020-038030.
74. Swanson LM, Burgess HJ, Zollars J, Todd Arnedt J. An open-label pilot study of a home wearable light therapy device for postpartum depression. *Arch Womens Ment Health.* (2018) (21:583–586. doi:10.1007/s00737-018-0836-z.
75. Li D, Fang P, Liu H, Chen L, Fu Y, Liu J, et al. The clinical effect of blue light therapy on patients with delayed sleep-wake phase disorder. *Nat Sci Sleep.* (2022) 14:75. doi:10.2147/nss.s344616.
76. Richardson C, Gradisar M. Depressed mood and repetitive negative thinking in delayed sleep–wake phase disorder: treatment effects and a comparison with good sleepers. *J Sleep Res.* (2022) 31(1):e13452. doi:10.1111/jsr.13452.
77. van Kol M. *A MOMENT OF BRIGHTNESS: The effects of brief morning Bright Light Therapy on burnout related*. [master’s thesis]. Eindhoven: Eindhoven University of Technology. (2015).
78. Raikes AC, Dailey NS, Shane BR, Forbeck B, Alkozei A, Killgore WD. Daily morning blue light therapy improves daytime sleepiness, sleep quality, and quality of life following a mild traumatic brain injury. *J Head Trauma Rehabil.* (2020) 35(5):E405–E421. doi:10.1097/htr.0000000000000579.
79. Lee SY, Aycock DM, Moloney MF. Bright light therapy to promote sleep in mothers of low-birth-weight infants: a pilot study. *Biol Res Nurs.* (2013) 15(4):398–406. doi:10.1177/1099800412445612.
80. Sasseville A, Martin JS, Houle J, H ́ebert M. Investigating the contribution of short wavelengths in the alerting effect of bright light. *Physiol Behav.* (2015) 151:81–87. doi:10.1016/j.physbeh.2015.06.028.
81. Goel N, Etwaroo GR. Bright light, negative air ions and auditory stimuli produce rapid mood changes in a student population: a placebo-controlled study. *Psychol Med.* (2006) 36(9):1253–1263. doi:10.1017/s0033291706008002.
82. Grandner MA. *Sleep, mood, and circadian responses to bright green light during sleep* [dissertation/ Doctor’s thesis]. University of California, San Diego and San Diego State University. (2007).
83. Papatheodorou G, Kutcher S. The effect of adjunctive light therapy on ameliorating breakthrough depressive symptoms in adolescent-onset bipolar disorder. *J Psychiatry Neurosci.* (1995) 20(3):226.
84. Figueiro MG, Rea MS. Office lighting and personal light exposures in two seasons: Impact on sleep and mood. *Lighting Res Technol.* (2016) 48(3):352–364. doi:10.1177/1477153514564098.
85. Figueiro MG, Steverson B, Heerwagen J, Kampschroer K, Hunter CM, Gonzales K, et al. The impact of daytime light exposures on sleep and mood in office workers. *Sleep Health.* (2017) 3(3):204–215. doi:10.1016/j.sleh.2017.03.005.
86. Higgins JP, Savovi ́c J, Page MJ, Elbers RG, Sterne JA. Assessing risk of bias in a randomized trial. *Cochrane handbook for systematic reviews of interventions.* (2019) p. 205–228. doi:10.1002/9781119536604.ch8.
87. Bogen S, Legenbauer T, Gest S, Holtmann M. Lighting the mood of depressed youth: Feasibility and efficacy of a (2 week-placebo controlled bright light treatment for juvenile inpatients. *J Affect Disord*. (2016) 190:450–456. doi:10.1016/j.jad.2015.09.026.
88. Spezzano MA (2006). *The efficacy of bright light treatment on the symptoms of seasonal affective disorder on a college-aged population* [doctor’s thesis]. Prescott, Arizona: Northcentral University. (2006).
89. Sterne JA, Savovi ́c J, Page MJ, Elbers RG, Blencowe NS, Boutron I, et al. RoB 2: a revised tool for assessing risk of bias in randomised trials. *BMJ*. (2019) 366. doi:10.1136/bmj.l4898.
90. Youngstedt SD, Kline CE, Ginsberg JP, Zielinski MR, Hardin JW. Bright light treatment for high-anxious young adults: a randomized controlled pilot study. *Depress Anxiety.* (2011) 28(4):324–332. doi:10.1002/da.20784.
91. Smith PF. A note on the advantages of using linear mixed model analysis with maximal likelihood estimation over repeated measures ANOVAs in psychopharmacology: comment on Clark et al (2012). *J Psychopharmacol.* (2012) 26(12):1605–1607. doi:10.1177/0269881112463471.
92. Nixon A, Strike MK, Feilds KL, Glozier N, Thatte S, Hickie IB, et al. Temporal dynamics of subjective sleep profiles predicting mood improvements during adjunctive light therapy combined with sleep rescheduling. *J Affect Disord Rep*. (2021) 4:100106. doi:10.1016/j.jadr.2021.100106.
93. Wyszecki G, Stiles WS. *Color science: concepts and methods, quantitative data and formulae*. John wiley & sons (2000).
94. Smith VC, Pokorny J. Spectral sensitivity of the foveal cone photopigments between 400 and 500 nm. *Vision Res*. (1975) 15(2):161–171. doi:10.1016/0042-6989(75)90203-5.
95. Snodderly D, Brown P, Delori F, Auran J. The macular pigment. I. Absorbance spectra, localization, and discrimination from other yellow pigments in primate retinas. *Invest Ophthalmol Vis Sci.* (1984) 25(6):660–673.
96. TC, C. *Light as a True Visual Quantity: Principles of Measurement*. CIE Publication, 41(1978).
97. Flory R, Ametepe J, Bowers B. A randomized, placebo-controlled trial of bright light and high-density negative air ions for treatment of Seasonal Affective Disorder. *Psychiatry Res.* (2010) 177(1-2):101–108. doi:10.1016/j.psychres.2008.08.011.
98. Madsen HØ, Ba-Ali S, Hageman I, Lund-Andersen H, Martiny K. Light therapy for seasonal affective disorder in visual impairment and blindness–a pilot study. *Acta Neuropsychiatr.* (2021) 33(4):191–199. doi:10.1017/neu.2021.6.
99. Lam RW, Teng MY, Jung YE, Evans VC, Gottlieb JF, Chakrabarty T, et al. Light therapy for patients with bipolar depression: systematic review and meta-analysis of randomized controlled trials. *Can J Psychiatry.* (2020) 65(5):290–300. doi:10.1177/0706743719892471.
100. Naus T, Burger A, Malkoc A, Molendijk M, Haffmans J. Is there a difference in clinical efficacy of bright light therapy for different types of depression? A pilot study. *J Affect Disord*. (2013) 151(3):1135–1137. doi:10.1016/j.jad.2013.07.017.
101. Chan JW, Lam S, Li SX, Chau SW, Chan S, Chan N, et al. Adjunctive bright light treatment with gradual advance in unipolar major depressive disorder with evening chronotype–A randomized controlled trial. *Psychol Med.* (2022) 52(8):1448–1457. doi:10.1017/s0033291720003232.
102. Carpenter JS, Crouse JJ, Scott EM, Naismith SL, Wilson C, Scott J, et al. Circadian depression: a mood disorder phenotype. *Neurosci Biobehav Rev.* (2021) 126:79–101. doi:10.1016/j.neubiorev.2021.02.045.
103. Terman M, Terman JS. Light therapy for seasonal and nonseasonal depression: efficacy, protocol, safety, and side effects. *CNS Spectr.* (2005) 10(8):647–663. doi:10.1017/s1092852900019611.
104. Diakoumis AA, ALP I, Aarts MM, Khademagha PP. *Characterization of light therapy devices* [master’s thesis]. Eindhoven: Eindhoven University of Technology. (2016).
105. Wirz-Justice A. From the basic neuroscience of circadian clock function to light therapy for depression: on the emergence of chronotherapeutics. *J Affect Disord*. (2009) 116(3):159. doi:10.1016/j.jad.2009.04.024.
106. Hou D, Lin C, Lin Y. Diurnal Circadian Lighting Accumulation Model: A Predictor of the Human Circadian Phase Shift Phenotype. *Phenomics.* (2022) 2(1):50–63. doi:10.1007/s43657-021-00039-6.
107. Kuwano N, Kato TA, Mitsuhashi M, Sato-Kasai M, Shimokawa N, Hayakawa K, et al. Neuron-related blood inflammatory markers as an objective evaluation tool for major depressive disorder: An exploratory pilot case-control study. *J Affect Disord*. (2018) 240:88–98. doi:10.1016/j.jad.2018.07.040.
108. Gadad BS, Jha MK, Czysz A, Furman JL, Mayes TL, Emslie MP, et al. Peripheral biomarkers of major depression and antidepressant treatment response: current knowledge and future outlooks. *J Affect Disord*. (2018) 233:3–14. doi:10.1016/j.jad.2017.07.001.
109. Berger C, Duck A, Gest S, Jonas L, Kolch M, Martin F, et al. Possible effects of bright light therapy on EEG-vigilance in the treatment of depression in adolescents–A pilot study. *Front Psychiatry.* (2022) p. 901. doi:10.3389/fpsyt.2022.820090.
